# Exercise modalities for adults with type 2 diabetes mellitus and obesity: comparative effects on visceral fat, metabolism, and inflammatory markers from a network meta-analysis

**DOI:** 10.3389/fimmu.2026.1825473

**Published:** 2026-08-25

**Authors:** Jinpeng Xu, Shuyue Luo, Wei Gao

**Affiliations:** 1 Shenyang Sport University, Shenyang, Liaoning, China; 2 Shenyang Sport University School of Marxism, Shenyang, Liaoning, China

**Keywords:** abdominal fat, exercise intervention, glucose and lipid metabolism, inflammatory factors, network meta-analysis, obesity, type diabetes mellitus

## Abstract

**Background:**

Obesity correlates closely with type 2 diabetes mellitus (T2DM), with central adiposity, dyslipidemia and chronic low-grade inflammation as typical pathological features. Physical exercise is recommended as a safe core non-pharmacological intervention, but the relative efficacy of different exercise modes for comprehensive metabolic improvement remains controversial and unclear.

**Objective:**

This network meta-analysis comprehensively compared the effects of diverse exercise interventions on abdominal fat distribution, glycolipid metabolism and inflammatory biomarkers in obese T2DM patients, aiming to determine the optimal exercise regimen for clinical individualized exercise prescription.

**Methods:**

We systematically searched five mainstream electronic databases for eligible RCTs published up to October 9, 2025. The Cochrane Risk of Bias Tool and GRADE system were used to assess study methodological quality and outcome evidence certainty. All statistical analyses were performed via Stata 16.0 and RevMan 5.4.

**Results:**

In total, 32 qualified RCTs involving 1519 obese T2DM participants were enrolled. No significant inconsistency was detected across all outcome indicators, so a consistency model was adopted for pooled analysis. Notably, exercise produced no significant change in total dietary energy intake. It significantly reduced BMI (MD = −1.78, 95% CI [−2.70, −0.85]), with combined aerobic-resistance training being optimal (SUCRA = 93%). Exercise also lowered TC (MD = −0.36, 95% CI [−0.59, −0.14], SUCRA = 94.2%), TG (MD = −0.28, 95% CI [−0.38, −0.19], SUCRA = 95%) and Homeostatic Model Assessment of Insulin Resistance (HOMA-IR) (MD = −0.92, 95% CI [−1.73, −0.10], SUCRA = 92.6%). LDL-C showed non-significant declining trend (SUCRA = 81.3%), while HDL-C increased markedly (MD = 0.12, 95% CI [0.05, 0.18], SUCRA = 97.5%). Aerobic exercise plus auxiliary treatments ranked first in reducing IL-6 (SUCRA = 99.9%). Walking and jogging worked best for reducing insulin level (SUCRA = 90.9%), and mind–body exercise maximized lean body mass (SUCRA = 93.7%).

**Conclusion:**

Regular exercise effectively ameliorates glycolipid metabolic disorders and chronic inflammatory status in obese T2DM patients. Combined aerobic-resistance training yields the most comprehensive metabolic benefits among all interventions. Importantly, these favorable metabolic improvements are independent of dietary energy restriction, which supports targeted individualized exercise prescriptions as an independent clinical intervention for this vulnerable population.

**Systematic review registration:**

https://www.crd.york.ac.uk/prospero/CRD420251272031, identifier CRD420251272031.

## 1 Introduction

In 2024, the global adult diabetes population reached 589 million, accounting for 11.1% of the global population in this age group. By 2045, this figure is projected to rise to 783 million (1). Type 2 diabetes mellitus (T2DM) is the predominant form of diabetes worldwide, accounting for over 90% of cases. Based on the 2024 global prevalence of 589 million people with diabetes, the number of T2DM patients is estimated at approximately 530 million (2). There are many factors contributing to the onset of T2DM, including obesity (3), prolonged sitting (4), Dietary habits (5) and unhealthy lifestyles (6) including factors such as Dietary adjustments and regular exercise are effective strategies for the prevention and management of T2DM (7, 8).

There exists a close bidirectional relationship between obesity and T2DM. Long-term excessive fat accumulation, particularly abdominal and visceral fat deposition, can promote the onset and progression of T2DM by exacerbating HOMA-IR and chronic low-grade inflammation (9). Diabetes itself can further exacerbate lipid metabolism disorders, creating a vicious metabolic cycle. Patients with T2DM and obesity typically exhibit abnormal abdominal fat accumulation, dyslipidemia, and elevated inflammatory factor levels. These alterations collectively contribute to the pathogenesis of glucose and lipid metabolism disorders (10). Patients with type 2 diabetes mellitus (T2DM) and obesity often require long-term energy restriction to improve glycemic and metabolic control, and their complications also significantly impact quality of life (11, 12). This adds to the psychological burden of patients with T2DM and obesity to some extent, subsequently affecting their dietary quality and health status (13).

Current diabetes interventions include lifestyle modifications, medication, and combined exercise and dietary interventions. International organizations typically recommend exercise as one of the primary management strategies for newly diagnosed T2DM patients. Exercise is a fundamental and primary element of diabetes and obesity prevention and management, underpinning lifestyle interventions alongside dietary and behavioral strategies (14, 15). Currently, the International Diabetes Federation recommends moderate-intensity aerobic training and high-intensity resistance training for patients with type 2 diabetes mellitus (T2DM) who are also obese (16). However, different regional guidelines recommend different types of exercise, and there is yet to be a unified consensus on implementation methods and standardization (17–23). Previous studies have shown that aerobic exercise of varying intensities improves glycemic control, lipid levels, and functional activity scores in patients with type 2 diabetes mellitus (24). Resistance training exerts a significant impact on cardiovascular and metabolic health indicators in patients with type 2 diabetes mellitus (T2DM) and obesity (25). Aerobic combined with resistance training exercise under dietary control improved BMI, cardiovascular risk factors, and muscle strength in patients with type 2 diabetes mellitus and obesity (26, 27). There is a relative lack of studies simultaneously measuring the effects of different exercise interventions on abdominal adipose tissue, obesity-induced lipid metabolism abnormalities, and chronic inflammation in populations with T2DM and obesity. Concurrently, constrained by study design limitations and the scarcity of direct comparisons, individual randomized controlled trials and traditional pairwise meta-analyses struggle to systematically evaluate the relative advantages of different physical activity modalities. Network meta-analysis integrates direct and indirect evidence to quantitatively compare the relative effectiveness of multiple interventions and rank their efficacy.

Therefore, by examining the effects of different intervention approaches on patients with T2DM and obesity, we compare the efficacy and therapeutic outcomes of various exercise training regimens in improving lipid metabolism and reducing inflammatory markers within abdominal adipose tissue in this patient population.

## 2 Methods

This study adheres to the recommendations of the PRISMA-NMA (see **Supplementary Materials**, **Supplementary Table 1**) and the Cochrane Handbook for Systematic Reviews of Interventions. It has been registered in the PROSPERO database (Registration ID: CRD420251272031).

### 2.1 Search strategy

This study employed a three-stage literature retrieval strategy. First, an initial search was conducted in the PubMed database using a combination of MeSH terms and free-text keywords to establish a systematic retrieval vocabulary. Second, based on the established retrieval strategy, a comprehensive search was performed across PubMed, Embase, Web of Science, The Cochrane Library, and Scopus, systematically including randomized controlled trials published from the inception of our database through October 9, 2025. Finally, we supplemented the search by tracing the reference lists of all included studies to identify potentially overlooked relevant research. The keyword combinations used were: (“obesity” or “overweight”) AND (“exercise” or “training” or “physical activity”) AND (“type 2 diabetes” or “diabetes mellitus, type 2” or “non-insulin-dependent diabetes mellitus”). For specific search strategies, please refer to the attached document see (**Supplementary Materials**, **Supplementary Table 2**).

### 2.2 Inclusion and exclusion criteria

Inclusion criteria are as follows: ① Population: Adult patients (≥18 years old) with clinically confirmed type 2 diabetes mellitus (T2DM) and overweight or obesity, regardless of gender, disease duration, or severity. ② Intervention Measures: Included interventions must incorporate at least one exercise-based component, including but not limited to: Aerobic exercise alone, Resistance training alone, Combined aerobic and resistance training, Aerobic or resistance training combined with other effective interventions (e.g., standard care or health management), Structured exercise formats such as mind-body exercise, walking, or jogging. Specific details regarding the content, format, and combination of various exercise interventions are outlined in the Intervention Classification Description see **Table 1**. ③ Comparison: The control group receives no exercise intervention, including routine care, health education, placebo intervention, or maintains their original lifestyle. ④ Outcomes: Studies must report at least one of the following outcome measures with extractable data. Outcomes were classified into primary and secondary outcomes based on reporting frequency across included studies and clinical evidence grade, consistent with standards for network meta-analysis.

**Table 1:** Definitions of included interventions.

Interventions	Definition
Blank Control Group	no exercise intervention, usual care, diet-only intervention, or nutritional supplementation.
Aerobic Exercise Only Group	moderate-intensity aerobic exercise beach walking, MICT.
Resistance Training Only Group	This category comprised resistance training and high-intensity interval training (HIIT) interventions in which high-intensity muscular contractions constituted the dominant training stimulus.
Aerobic Plus Resistance Training Group	This intervention category included exercise programs that integrated aerobic exercise and resistance training within the same intervention period, either performed in the same session or on separate days. Aerobic exercise components consisted of activities such as walking, jogging, cycling, or other moderate-intensity continuous aerobic exercises, while resistance training comprised machine-based, free-weight, or body-weight resistance exercises.
Aerobic Exercise Combined with Other Effective Treatments	This category included aerobic exercise interventions combined with dietary modification or nutritional supplementation, such as aerobic exercise plus dietary intervention, aerobic exercise plus saffron supplementation, aerobic exercise plus green tea, and aerobic exercise plus zucchini supplementation.
Resistance Training Combined with Other Effective Treatments	This category included interventions in which resistance training was the core exercise modality, combined with specific nutritional or metabolic adjunctive treatments. Resistance training consisted of machine-based, free-weight, or body-weight exercises, while the co-interventions involved dietary carbohydrate modulation, protein supplementation, or other structured nutritional strategies (e.g., resistance training plus carbohydrate intake regulation or protein supplementation).
Mind-Body Exercise Group	This category included interventions characterized by integrated physical movement, breathing regulation, and mental focus, with Tai Chi as the primary modality.
Walking and Jogging Group	This category included structured walking-based interventions, such as continuous walking, intermittent walking, interval walking, and light jogging, performed at low-to-moderate intensity.
Pharmacological or Psychological Intervention Group	This category included non-exercise adjunctive interventions aimed at improving metabolic or psychological outcomes, such as nutritional supplementation (e.g., saffron, zucchini, green tea), macronutrient manipulation (e.g., carbohydrate-modified diets, high-protein diets), and structured dietary interventions.
Aerobic Plus Resistance Training Combined with Other Effective Treatments	This group integrates aerobic exercise, resistance training, and one or more additional effective therapeutic measures (e.g., dietary modification, nutritional supplementation, etc.) for patients with type 2 diabetes mellitus combined with obesity.

Primary outcomes were defined as indicators with high reporting frequency (reported in ≥12 RCTs) and high clinical relevance to the core research question, reflecting key pathological characteristics in patients with type 2 diabetes and obesity. These included: BMI, total cholesterol (TC), triglycerides (TG), low-density lipoprotein cholesterol (LDL-C), high-density lipoprotein cholesterol (HDL-C), HOMA-IR (Homeostatic Model Assessment of Insulin Resistanceis a widely validated mathematical index used to quantitatively evaluate peripheral insulin resistance, calculated from fasting plasma glucose and fasting insulin concentrations via the formula: HOMA-IR=Fasting glucose (mmol/L)×Fasting insulin (mIU/L)/22.5). Secondary outcomes were defined as indicators with lower reporting frequency (<12 RCTs) and moderate evidence grade, which were indirectly related to core metabolic and inflammatory improvements but provided supplementary descriptive information. These included inflammatory markers interleukin-6, Total intake, Insulin, Lean body mass. ⑤ Study Design: Include randomized controlled trials (RCTs). Exclusion criteria include: ① Literature where full-text access is unavailable (e.g., only titles or abstracts provided, with unsuccessful attempts to contact authors or corresponding authors); ② Studies published in languages other than Chinese or English; ③ Animal studies; ④ Books, conference proceedings, or registration protocols; ⑤ Studies focusing on patients with obesity and other chronic diseases, or on children, adolescents, or pregnant women; ⑥ Reviews or meta-analyses; ⑦ Studies with irrelevant research content. For specific PICOS inclusion criteria, please refer to the attached document see **Supplementary Materials** (**Supplementary Materials**, **Supplementary Table 3**).

### 2.3 Data selection and extraction

Duplicate studies were removed using Endnote X9 software. Two researchers (Jinpeng Xu and Shuyue Luo) conducted an initial independent screening of titles and abstracts. Following this, full-text articles were read and re-screened against the inclusion criteria. Any discrepancies were resolved through discussion or by involving a third researcher (Wei Gao). Data extraction was performed from the included studies, encompassing authors, publication year, study origin (country), sample size, age, intervention type, intervention duration, and outcomes.

### 2.4 Quality assessment

This study systematically evaluated the methodological quality of the included studies using the Cochrane Collaboration’s recommended tool for assessing the risk of bias in randomized controlled trials (28). Two researchers (Jinpeng Xu and Shuyue Luo) independently assessed the risk of bias for each randomized controlled trial across seven domains: random sequence generation (selection bias), allocation concealment (selection bias), blinding of participants and personnel (performance bias), blinding of outcome assessment (detection bias), incomplete outcome data (attrition bias), selective reporting (reporting bias), and other biases. Each domain was assessed using standardized signal questions and categorized as “low risk of bias,” “unclear,” or “high risk of bias.” Any discrepancies were resolved through discussion or by involving a third researcher (Wei Gao).

### 2.5 Statistical analysis

All statistical analyses were performed using the network command suite in Stata software (version 16.0), with RevMan 5.4 used for pairwise meta-analysis and effect size conversion. Continuous outcome measures were expressed as either mean differences (MD) or standardized mean differences (SMD) with 95% confidence intervals (CI), depending on the consistency of measurement tools and units across included studies:

MD was applied when all studies used the same measurement method and unit for a given outcome (e.g., visceral fat measured by CT in cm², fasting glucose in mmol/L), as it preserves the original clinical scale and facilitates direct interpretation of intervention effects.

SMD was used when studies adopted different measurement tools, units, or scales for the same construct (e.g., insulin resistance assessed by HOMA-IR vs. QUICKI, inflammatory markers measured by ELISA vs. chemiluminescence), to standardize effect sizes across heterogeneous measurement methods. SMD was calculated as the difference in mean values between intervention and control groups divided by the pooled standard deviation, eliminating unit-related biases and enabling synthesis of results from diverse measurement approaches. Conversion between MD and SMD was performed automatically using the built-in function in RevMan 5.4, ensuring consistency in effect size calculation.

When the original studies reported the standard error (SE) of the mean, the standard deviation (SD) was calculated using the formula SD = SE × When SD or other measures of variability were not directly reported, SD was derived from confidence intervals, t-values, quartiles, ranges, or p-values, following the methodology outlined in Section 7.7 of the Cochrane Handbook for Systematic Reviews of Interventions (28).

Initially, we conducted a meta-analysis with exercise as the experimental group and the placebo group as the control group to first validate the effects of exercise on patients with type 2 diabetes and obesity. The I² statistic was used to assess heterogeneity among studies (29), if I² > 50, use random-effects models; if ≤ 50, use fixed-effects models. To further validate the robustness of the research conclusions, sensitivity analyses were conducted. During the meta-analysis process, randomized controlled trials with higher risk of bias were sequentially excluded to assess the stability of the results (30). Subsequently, a network meta-analysis was conducted. A network geometry diagram was used to visualize the evidence structure of different exercise interventions in patients with type 2 diabetes and obesity. In the network diagram, each node represents a distinct exercise intervention, with node area proportional to the number of participants receiving that intervention. Connections between nodes indicate direct comparisons between interventions, with line width reflecting the number of associated studies. Closed loops were evaluated for consistency between direct and indirect evidence using global inconsistency tests and node splitting analysis, with P values > 0.05 indicating no inconsistency. Finally, ranked order analysis was performed on the included exercise interventions, calculating their ranking probabilities and estimating the cumulative area under the ranking curve (SUCRA). SUCRA values range from 0 to 1, with higher values indicating greater likelihood of an exercise intervention achieving optimal outcomes. To assess potential small-sample effects and publication bias, a comparison-adjusted network funnel plot was used for visual inspection of result symmetry (31). All statistical analyses were performed using Stata 16.0 software and RevMan 5.4 software.

### 2.6 Evidence quality assessment

The certainty of evidence for each outcome was assessed using the Grading of Recommendations, Assessment, Development, and Evaluations (GRADE) approach, following the GRADE 2024 guidelines for network meta-analysis. Randomized controlled trials (RCTs) were initially rated as high-certainty evidence, and downgraded based on five core domains: risk of bias (study limitations), inconsistency (heterogeneity), indirectness, imprecision, and publication bias. The final certainty of evidence was classified into four levels: high, moderate, low, and very low. A detailed GRADE evidence summary table is presented in **Supplementary Table** (**Supplementary Materials**; **Appendix 4**).

## 3 Result

### 3.1 Study selection, inclusion, and characteristics

**Figure 1:**
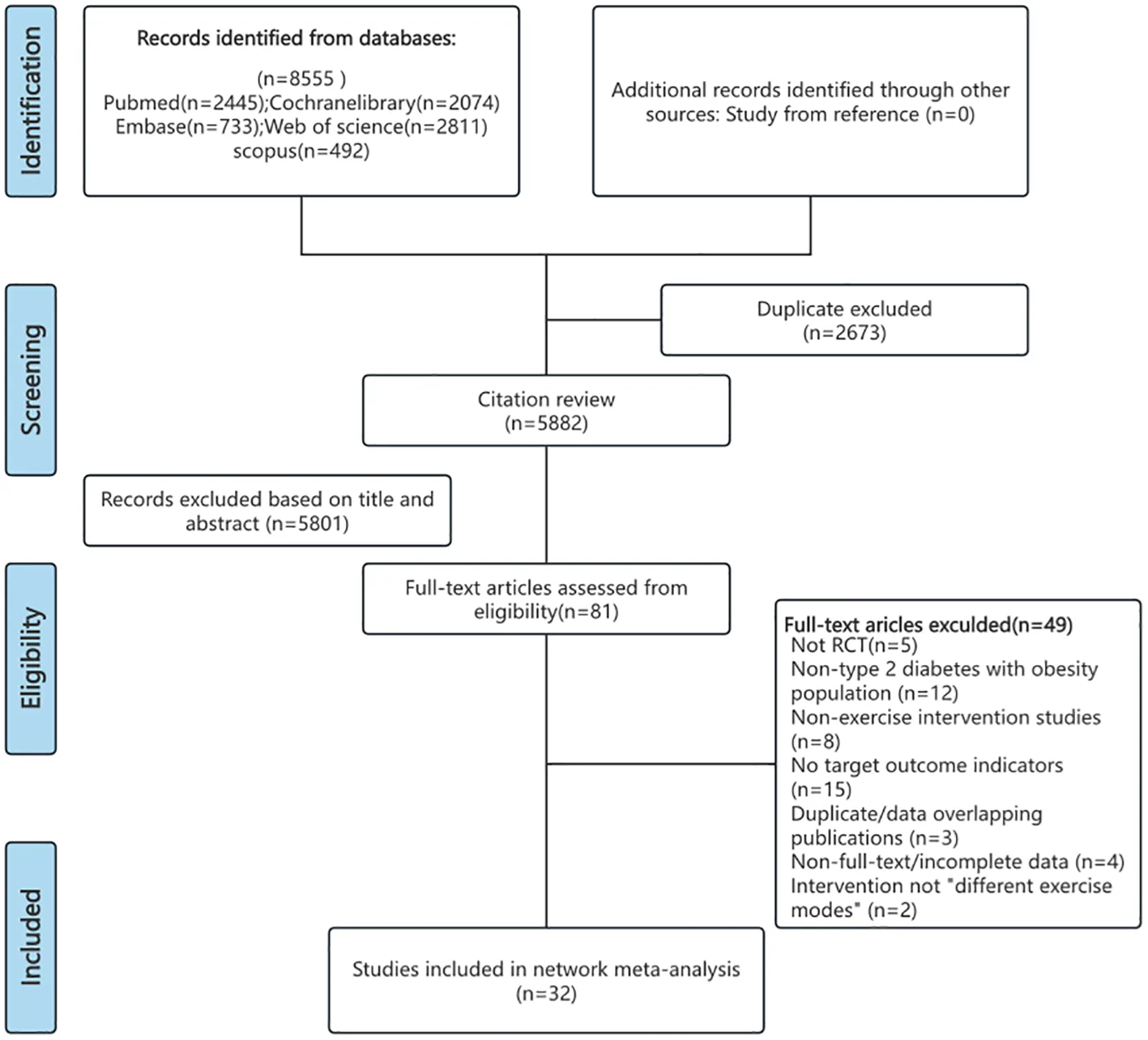
Filtering process diagram.

A total of 8,555 articles were identified. After removing 2,673 duplicates, two researchers (Jinpeng Xu and Shuyue Luo) independently screened titles and abstracts, eliminating 5,801 articles. Ultimately, 81 full-text articles were reviewed. Following further eligibility assessment, 49 articles were excluded for non-compliance with inclusion criteria. Ultimately, 32 RCTs were included in the network meta-analysis (See **Table 2** for details.). The detailed screening process is illustrated in **Figure 1**.

**Table 2:** Information related to 32 randomized controlled trials included in the network meta-analysis.

Author	Country	Age	Study period	SampleN/F/M	Intervention measures	Outcome indicators
Walid Kamal Abdelbasset, 2020 (32)	Egypt	40-60	8 weeks	16/6/10	High-intensity interval training, 3 sessions/week, 40 min/session	BMI, TC, TG, LDL, HDL
		40-60	8 weeks	16/7/9	No structured exercise intervention	BMI, TC, TG, LDL, HDL
Mohamed Seyam, 2020 (33)	Saudi Arabia	52.8	4 months	33/13/20	Sand Walking Group Group, 3 sessions/week, 30 min/session	BMI
		52.1	4 months	33/15/18	Flat Ground Walking No structured exercise intervention, 3 sessions/week, 30 min/session	BMI
Shehab M Abd El-Kader, 2018 (34)	Egypt	48.73 ± 6.26	12 weeks	40/17/23	Aerobic + Resistance Group, 3 sessions/week, 30 min/session	Insulin, HOMA-IR
		50.12 ± 5.91	12 weeks	40/15/25	No structured exercise intervention	Insulin, HOMA-IR
Sampath Kumar Amaravadi, 2024 (35)	India	56.05 ± 8.77	12 weeks	75/17/58	Yoga Group Group, 3–5 sessions/week, 45–65 min/session	FFM, Insulin, HOMA-IR
		53.90 ± 10.2	12 weeks	71/32/39	No structured exercise intervention	FFM, Insulin, HOMA-IR
AminiLari, 2017 (36)	Iran	45–60	12 weeks	12/12/0	Resistance Group, 3 sessions/week, 45 min/session	BMI, FFM
		45–60	12 weeks	12/12/0	Aerobic Group, 3 sessions/week, 45 min/session	BMI, FFM
		45–60	12 weeks	13/13/0	Aerobic + Resistance Group, 3 sessions/week, 45 min/session	BMI, FFM
		45–60	12 weeks	15/15/0	No structured exercise intervention	BMI, FFM
Dunstan, 2002 (37)	Australia	67.6 ± 5.2	6 months	16/6/10	Resistance + Weight Loss Group, 3 sessions/week, 45 min/session	BMI, TC, TG, LDL, HDL, Insulin, HOMA-IR, Total Intake
		66.9 ± 5.3	6 months	13/7/6	Weight Loss No structured exercise intervention	BMI, TC, TG, LDL, HDL, Insulin, HOMA-IR, Total Intake
Golpasandi, 2025 (38)	Iran	46.37 ± 4.27	8 weeks	8/0/8	Green tea+HIIT Group, 3 sessions/week, 40 min/session	BMI, Insulin, HOMA-IR
		46.75 ± 5.28	8 weeks	8/0/8	Placebo+HIIT Group, 3 sessions/week, 40 min/session	BMI, Insulin, HOMA-IR
		47.75 ± 5.89	8 weeks	8/0/8	Green tea, 3 sessions/week	BMI, Insulin, HOMA-IR
		48.50 ± 5.92	8 weeks	8/0/8	No structured exercise intervention	BMI, Insulin, HOMA-IR
Hooshmand Moghadam, 2022 (39)	Iran	39 ± 5	12 weeks	13/0/13	Training + Saffron Group, 7 sessions/week, 60–90 min/session	BMI, Insulin, HOMA-IR, Total Intake
		38 ± 5	12 weeks	13/0/13	Training + Placebo Group, 7 sessions/week, 60–90 min/session	BMI, Insulin, HOMA-IR, Total Intake
		40 ± 5	12 weeks	13/0/13	Saffron No structured exercise intervention	BMI, Insulin, HOMA-IR, Total Intake
		39 ± 5	12 weeks	13/0/13	Placebo No structured exercise intervention	BMI, Insulin, HOMA-IR, Total Intake
Jennings, 2009 (40)	Canada	55 ± 7	26 weeks	25/6/19	Resistance Group, 3 sessions/week, 30–45 min/session	FFM
		55 ± 7	26 weeks	20/7/13	Aerobic Group, 3 sessions/week, 30–45 min/session	FFM
		53 ± 8	26 weeks	25/6/19	Aerobic + Resistance Group, 3 sessions/week, 30–45 min/session	FFM
		56 ± 7	26 weeks	33/11/22	No structured exercise intervention	FFM
Karstoft, 2013 (41)	Denmark	59.5 ± 2.3	4 months	12/5/7	Aerobic Training Group, 5 sessions/week, 60 min/session	BMI, TC, TG, LDL, HDL, Insulin, HOMA-IR
		57.1 ± 3	4 months	8/3/5	No structured exercise intervention	BMI, TC, TG, LDL, HDL, Insulin, HOMA-IR
Magalhães, 2019 (42)	Portugal	59.7 ± 6.5	12 months	25/10/15	HIIT Group, 3 sessions/week, 50 min/session	FFM
		56.7 ± 8.3	12 months	28/15/13	MICT Group, 3 sessions/week, 50 min/session	FFM
		59 ± 8.1	12 months	27/13/14	No structured exercise intervention	FFM
Miller, 2021 (27)	Australia	61.1 ± 6.2	24 weeks	98/36/62	Aerobic + Dietary Group, 3 sessions/week, 45–60 min/session	BMI, Insulin, HOMA-IR, IL-6
		62 ± 6.2	24 weeks	100/36/64	No structured exercise intervention	BMI, Insulin, HOMA-IR, IL-6
Rajabi, 2022 (43)	Iran	51.5 ± 6.16	8 weeks	8/8/0	Aerobic + saffron, 3 sessions/week, 35–65 min/session,	BMI, Insulin, HOMA-IR
		57.62 ± 6.81	8 weeks	8/8/0	Aerobic+ placebo, 3 sessions/week, 35–65 min/session	BMI, Insulin, HOMA-IR
		57.62 ± 6.81	8 weeks	8/8/0	saffron	BMI, Insulin, HOMA-IR
		56.87 ± 5.11	8 weeks	8/8/0	No structured exercise intervention	BMI, Insulin, HOMA-IR
Rezaeeshirazi, 2022 (44)	Iran	21.07 ± 2.4	8 weeks	13/0/13	Resistance Group, 4 sessions/week, 30 min/session	BMI, FFM
		21.29 ± 1.9	10 weeks	14/0/14	Aerobic Group, 4 sessions/week, 40 min/session	BMI, FFM
		22.47 ± 2.03	10 weeks	15/0/15	No structured exercise intervention	BMI, FFM
Sabag, 2020 (45)	Australia	54.8 ± 2.4	12 weeks	10/6/4	Medium intensity, 3 sessions/week, 40–55 min/session	BMI, TC, TG, LDL, HDL, HOMA-IR
		56.9 ± 2.1	12 weeks	13/6/7	High strength, 3 sessions/week, 40–55 min/session	BMI, TC, TG, LDL, HDL, HOMA-IR
		51.9 ± 1.4	12 weeks	10/3/7	No structured exercise intervention	BMI, TC, TG, LDL, HDL, HOMA-IR
Sokolovska, 2020 (46)	Latvia	60.64 ± 7.4	Four months	14/8/6	Moderate Aerobic Group, 3 sessions/week, 40 min/session	BMI, TC, TG, LDL, HDL, FFM
		60.69 ± 8.8	4 months	26/19/7	No structured exercise intervention	BMI, TC, TG, LDL, HDL, FFM
Wycherley, 2010 (47)	Australia	56.1 ± 7.5	16 weeks	17/-/-	Exercise + Diet Group, 3 sessions/week, 45 min/session	BMI, TC, TG, LDL, HDL, Insulin, HOMA-IR
		56.1 ± 7.5	16 weeks	16/-/-	No structured exercise intervention	BMI, TC, TG, LDL, HDL, Insulin, HOMA-IR
Yamaguchi, 2011 (48)	Japan	not report	4 weeks	11/-/-	Walking Group Group, 7 sessions/week, 30 min/session	FFM, Insulin
		not report	4 weeks	8/-/-	No structured exercise intervention	FFM, Insulin
Daly, 2005 (25)	Australia	67.6 ± 5.2	12 months	16/6/10	Low-Intensity Exercise Group, 3 sessions/week, 45–55 min/session	BMI, TC, TG, LDL, HDL, Insulin, HOMA-IR
		66.9 ± 5.3	12 months	13/6/7	No structured exercise intervention	BMI, TC, TG, LDL, HDL, Insulin, HOMA-IR
Otten, 2017 (49)	Sweden	58–66	12 weeks	14/5/9	Aerobic Training Group, 3 sessions/week, 60 min/session	TC, TG, LDL, HDL, FFM, Insulin, HOMA-IR
		53–64	12 weeks	15/5/10	No structured exercise intervention	TC, TG, LDL, HDL, FFM, Insulin, HOMA-IR
Otten, 2018 (50)	Sweden	58.67	12 weeks	13/5/8	Resistance Training Group, 3 sessions/week, 60 min/session	BMI, TC, TG, LDL, HDL, HOMA-IR
		54–64	12 weeks	13/4/9	No structured exercise intervention	BMI, TC, TG, LDL, HDL, HOMA-IR
Rajabi, 2024 (51)	Iran	54.1 ± 5.6	12 weeks	11/11/0	Saffron + training, 3 sessions/week, 20–25 min/session	TC, TG, LDL, HDL, Insulin, HOMA-IR, Total Intake
		53.8 ± 5.9	12 weeks	11/11/0	Placebo + training, 3 sessions/week, 20–25 min/session	TC, TG, LDL, HDL, Insulin, HOMA-IR, Total Intake
		54.5 ± 5.4	12 weeks	11/11/0	Saffron, 3 sessions/week, No structured exercise intervention	TC, TG, LDL, HDL, Insulin, HOMA-IR, Total Intake
		54.2 ± 5.7	12 weeks	11/11/0	No structured exercise intervention	TC, TG, LDL, HDL, Insulin, HOMA-IR, Total Intake
Saeidi, 2021 (52)	Iran	54.1 ± 5.6	Twelve weeks	11/11/0	Training + Broccoli, 3 sessions/week, 75 min/session	BMI, TC, TG, LDL, HDL, Insulin, HOMA-IR, IL-6, Total Intake
		53.8 ± 5.9	12 weeks	11/11/0	Training + Supplements, 3 sessions/week, 75 min/session	BMI, TC, TG, LDL, HDL, Insulin, HOMA-IR, IL-6, Total Intake
		54.5 ± 5.4	12 weeks	11/11/0	Broccoli, 3 sessions/week, No structured exercise intervention	BMI, TC, TG, LDL, HDL, Insulin, HOMA-IR, IL-6, Total Intake
		54.2 ± 5.7	12 weeks	11/11/0	No structured exercise intervention	BMI, TC, TG, LDL, HDL, Insulin, HOMA-IR, IL-6, Total Intake
Sanudo, 2013	Spain	72 ± 8	12 weeks	20/9/11	Cycling Group, 3 sessions/week, 12–20 min/session	BMI, IL-6
		67 ± 11	12 weeks	20/10/10	No structured exercise intervention	BMI, IL-6
Winding, 2018 (53)	Denmark	54 ± 6	11 weeks	12/5/7	Endurance training Group, 3 sessions/week, 45 min/session	BMI, Total Intake
		58 ± 8	11 weeks	13/6/7	High-intensity training group, 3 sessions/week, 20 min/session	BMI, Total Intake
		57 ± 7	11 weeks	7/2/5	No structured exercise intervention	BMI, Total Intake
Wycherley, 2008 (54)	Australia	51.2 ± 2.4	12 weeks	13/7/6	Resistance Group, 4–5 sessions/week, 55–60 min/session	BMI, FFM, Insulin
		53 ± 1.8	12 weeks	16/6/10	No structured exercise intervention	BMI, FFM, Insulin
Chen, 2010 (55)	China	59.1 ± 6.2	12 weeks	56/31/25	Tai Chi Group, 3 sessions/week, 60 min/session	BMI, TC, TG, LDL, HDL
		57.4 ± 5.8	12 weeks	48/28/20	No structured exercise intervention	BMI, TC, TG, LDL, HDL
Banitalebi, 2019 (56)	Iran	53.36 ± 5.94	10 weeks	14/14/0	Aerobic + Resistance Group, 3 sessions/week, 50 min/session	BMI, Insulin, HOMA-IR, IL-6
		54.14 ± 5.43	10 weeks	14/14/0	Sprint Interval, 3 sessions/week, 50 min/session	BMI, Insulin, HOMA-IR, IL-6
		55.71 ± 6.4	10 weeks	14/14/0	No structured exercise intervention	BMI, Insulin, HOMA-IR, IL-6
Bonfante, 2022 (57)	Brazil	51.1 ± 3.94	8 weeks	17/`0/7	Aerobic + Resistance Group, 3 sessions/week, 140 min/session	FFM, Insulin, Total Intake
		52.41 ± 4.44	8 weeks	17/8/9	No structured exercise intervention	FFM, Insulin, Total Intake
Bacchi, 2013 (58)	Italy	55.6 ± 2	16 weeks	13/3/10	Aerobic Group, 3 sessions/week, 60 min/session	BMI, TC, TG, LDL, HDL, Insulin, Total Intake
		56 ± 1.9	16 weeks	17/5/12	Resistance Group, 3 sessions/week, 60 min/session,	BMI, TC, TG, LDL, HDL, Insulin, Total Intake
Gholaman, 2018 (59)	Iran	44.2 ± 2.5	8 weeks	10/10/0	Zucchini + Exercise Group, 3 sessions/week, 60 min/session,	BMI, TC, TG, HOMA-IR
		44.2 ± 2.5	8 weeks	10/10/0	Exercise Group, 3 sessions/week, 60 min/session,	BMI, TC, TG, HOMA-IR
		44.2 ± 2.5	8 weeks	10/10/0	Zucchini, 3 sessions/week, 60 min/session,	BMI, TC, TG, HOMA-IR
		44.2 ± 2.5	8 weeks	10/10/0	No structured exercise intervention	BMI, TC, TG, HOMA-IR
Sabine Lambers, 2008 (60)	Belgium	55.8 ± 9.66	12 months	17/7/10	Aerobic + saffron, 3 sessions/week, 50 min/session	BMI, Insulin, HOMA-IR
		52.2 ± 8.26	12 months	18/2/16	Aerobic + Resistance Group, 3 sessions/week, 50 min/session,	BMI, Insulin, HOMA-IR
		57.5±	12 months	11/5/6	No structured exercise intervention	BMI, Insulin, HOMA-IR

BMI, Body Mass Index; FFM, Fat-Free Mass; TC, Total Cholesterol; TG, Triglycerides; LDL-C, Low-Density Lipoprotein Cholesterol; HDL-C, High-Density Lipoprotein Cholesterol; HOMA-IR, Homeostasis Model Assessment of Insulin Resistance; IL-6, Interleukin-6; Total Intake, Total Energy Intake.

This study included 1,519 participants who received 10 different intervention approaches. Details of the interventions are as follows: (1) Control group (n=396), (2) Aerobic exercise only (n=258), (3) Resistance training only (n=141), (4) Aerobic plus resistance training (n=134), (5) Aerobic combined with other effective therapies (n=86), (6) Resistance combined with other effective therapies (n=136), (7) Mind-body exercise (n=50), (8) Walking and jogging (n=49), (9) Pharmacological or psychological treatment (n=156), (10) Aerobic plus resistance combined with other effective therapies (n=113).

### 3.2 Methodological quality of the research

**Figure 2:**
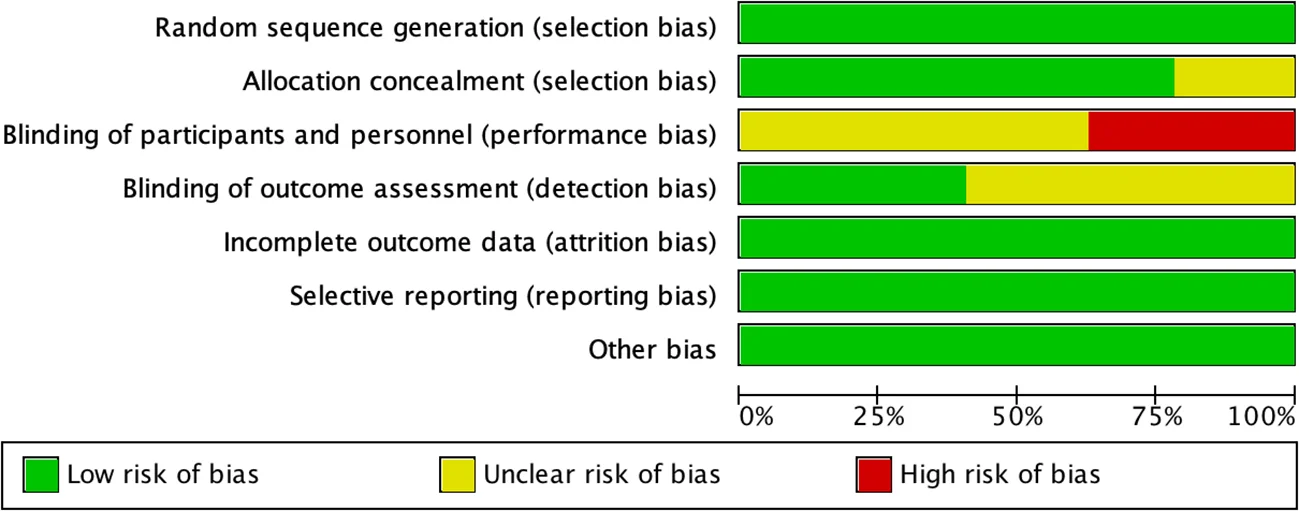
Risk of bias graph.

**Figure 3:**
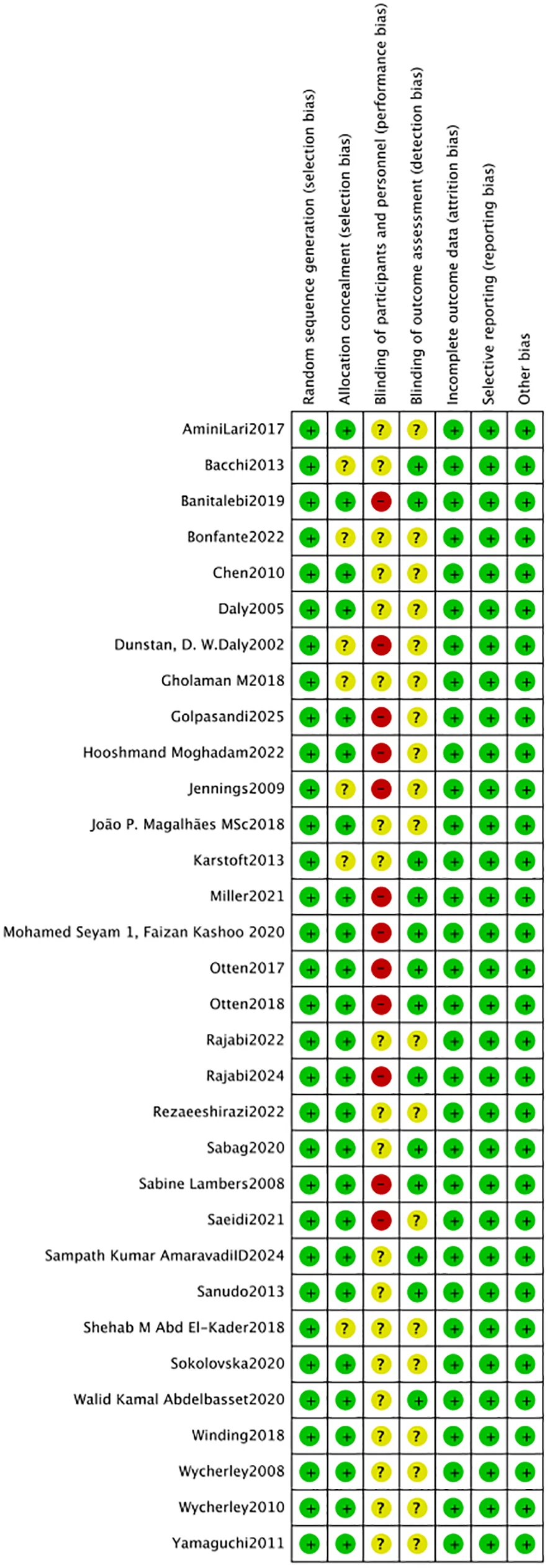
Risk of bias summary.

Using the Revman 5.4 tool, the risk of bias across included studies showed that all studies had a low risk of bias for random sequence generation. Twenty-five studies were at low risk of bias for allocation concealment, while seven studies had unclear risk of bias. Regarding blinding of participants and personnel (performance bias), 20 studies had unclear risk of bias, with 12 studies at high risk of bias. This may be due to the inherent difficulty of blinding in exercise interventions. Regarding outcome assessor blinding (detection bias), 13 studies had low risk of bias, while 19 studies had unclear risk of bias. Additionally, all studies had low risk of bias for incomplete outcome data (attrition bias), selective reporting (reporting bias), and other biases. (**Figures 2**, **3**).

### 3.3 Network meta-analysis

#### 3.3.1 Inconsistency assessment

BMI, total cholesterol, triglycerides, LDL cholesterol, HDL cholesterol, HOMA-IR, interleukin, total intake, lean body mass, and insulin form a closed loop, thus inconsistency tests were conducted. Results indicate that all inconsistency test P-values are >0.05, with no significant inconsistencies detected. This demonstrates good network consistency, warranting the use of the consistency model for subsequent analysis.

#### 3.3.2 Primary outcome measures

Primary outcomes included indicators closely related to the core research objectives (improving metabolic disorders and chronic inflammation in obese T2DM patients), with high reporting frequency (≥12 RCTs) and high evidence grade, including BMI, TC, TG, LDL-C, HDL-C, and insulin resistance (HOMA-IR). These indicators are widely recognized as key markers for evaluating the efficacy of exercise interventions in obese T2DM patients, with consistent clinical measurement standards and strong relevance to the research hypothesis.

##### 3.3.2.1 BMI

Of the 32 randomized controlled trials included, 25 reported BMI as an outcome measure (32–36, 38, 41–48, 50–56, 58–61). The specific network diagram is shown in Figure (**Supplementary Materials**; **Appendix 1 F1**). The relative effects of different interventions on BMI in patients with T2DM and obesity are presented in Table (Supplementary Materials, Appendix 2 F1). Meta-analysis revealed that compared with the control group, exercise significantly improved total energy intake in patients with type 2 diabetes and obesity (MD = -1.78, 95% CI [-2.70, -0.85], I² = 92). The certainty of evidence for this outcome was rated as low, downgraded due to very serious inconsistency (high heterogeneity) and not serious risk of bias (Supplementary Materials, Appendix 4 F1). Network meta-analysis results indicate that the aerobic plus resistance training group (SMD = -3.67, 95% CI [-4.87, -2.47]), the mind-body exercise group (SMD = -3.32, 95% CI [-6.39, -0.25]), the aerobic-only group (SMD = -1.82, 95% CI [-2.68, -0.96]), aerobic plus resistance combined with other effective treatments (SMD = -1.85, 95% CI [-3.50, -0.20]), resistance training alone (SMD = -1.78, 95% CI [-3.27, -0.29]), and aerobic plus resistance training (SMD = -1.43, 95% CI [-2.63, -0.22]) were more effective than the control group. Aerobic plus resistance training had the highest probability of being the optimal treatment (93%), followed by mind-body exercise (82.1%) and aerobic exercise alone (58.1%) (**Table 3**).

**Table 3:** SUCRA rankings of different exercise interventions by outcome indicators.

Intervention methods indicator	BMI	TC	TG	LDL-C	HDL-C	HOMA-IR	IL-6	Insulin	Lean body mass
CON	7.6	15	9.1	17.1	49.9	8.5	6.4	23.8	51.6
AER	58.1	43.3	38.9	9.6	33.7	44.4	48.8	35.8	27.5
RES	55.8	62.3	16.2	–	77.8	31.8	–	7.8	26.3
AZO	45.2	64.5	82.6	63.9	22.1	57.1	58.4	64.4	54.3
AOT	93.0	94.2	49.1	–	0.1	92.6	99.9	69.6	–
ROT	45.8	67.3	55.1	81.3	97.5	47.9	–	–	61.1
MBO	82.1	37.9	67.2	–	21.0	–	–	–	–
WJO	21.9	26.3	63.5	47.1	61.7	63.0	–	90.9	40.2
PSY	33.1	23.9	24.2	75.3	90.5	59.4	14.1	1.7	93.7
AZOT	57.4	66.3	95.0	55.7	45.6	–	72.4	–	45.4

Blank control group (CON), Aerobic exercise only (AER), Resistance exercise only (RES), Aerobic plus resistance exercise (AZO), Aerobic exercise combined with other effective treatments (AOT), Resistance exercise combined with other effective treatments (ROT), Mind-body exercise group (MBO), Walking and jogging group (WJO), Pharmacological or psychological group (PSY), Aerobic exercise plus resistance exercise combined with other effective treatments (AZTO).

##### 3.3.2.2 Total cholesterol

Of the 32 randomized controlled trials included, 15 reported total cholesterol as an outcome measure (32, 41, 45–49, 52–55, 57–60). The specific network diagram is shown in Figure (**Supplementary Materials, Appendix 1 F2**). The relative effects of different interventions on total cholesterol in patients with T2DM and obesity are presented in Figure (**Supplementary Materials, Appendix 2 F2**). Meta-analysis revealed that exercise significantly improved total intake in patients with type 2 diabetes and obesity compared to the control group (MD = -0.36, 95% CI [-0.59, -0.14], I² = 89). The certainty of evidence was low, downgraded due to very serious inconsistency and not serious risk of bias (Supplementary Materials, Appendix 4 F2). Network meta-analysis results indicate that aerobic exercise combined with other effective treatments (SMD = -1.24, 95% CI [-1.95, -0.53]) and aerobic exercise plus resistance training (SMD = -0.56, 95% CI [-1.05, -0.06]) were more effective than the control group. Aerobic exercise combined with other effective treatments had the highest probability (94.2%) of being the optimal treatment, followed by resistance training combined with other effective treatments (67.3%) and aerobic exercise plus resistance training combined with other effective treatments (66.3%) (**Table 3**).

##### 3.3.2.3 Triglycerides

Of the 32 randomized controlled trials included, 16 reported triglycerides as an outcome measure (32, 37, 41, 45–49, 52–55, 57–60). The specific network diagram is shown in Figure (**Supplementary Materials, Appendix 1 F3**). The relative effects of different interventions on triglycerides in patients with T2DM and obesity are shown in Figure (**Supplementary Materials, Appendix 2 F3**). Meta-analysis revealed that exercise significantly improved total caloric intake in obese T2DM patients compared to controls (MD = -0.28, 95% CI [-3.80, -0.19], I² = 58). The certainty of evidence was moderate, downgraded due to serious inconsistency, with no other significant limitations (**Supplementary Materials, Appendix 4 F3**). Network meta-analysis results indicated that the aerobic plus resistance training group combined with other effective treatments (SMD = -0.66, 95% CI [-0.96, -0.36]), the aerobic plus resistance training group (SMD = -0.51, 95% CI [-0.76, -0.26]), psychophysical exercise group (SMD = -0.38, 95% CI [-0.74, -0.02]), walking and jogging group (SMD = -0.34, 95% CI [-0.61, -0.08]), and aerobic exercise alone group (SMD = -0.18, 95% CI [-0.35, -0.01]) were more effective than the control group. Aerobic plus resistance training combined with other effective therapies had the highest probability (95%) of being the optimal treatment, followed by aerobic plus resistance training (82.6%) and mind-body exercise (67.2%) (**Table 3**).

##### 3.3.2.4 Low-density lipoprotein cholesterol

Of the 32 randomized controlled trials included, 12 reported low-density lipoprotein cholesterol as an outcome measure (32, 37, 41, 45–48, 52–54, 57, 58). The specific network diagram is shown in Figure (**Supplementary Materials, Appendix 1 F4**). The relative effects of different interventions on low-density lipoprotein cholesterol in patients with T2DM and obesity are shown in Figure (**Supplementary Materials, Appendix 2 F4**). Meta-analysis revealed that exercise significantly improved total energy intake in obese T2DM patients compared to controls (MD = -0.19, 95% CI [-0.35, 0.04], I² = 81). The certainty of evidence was very low, downgraded due to serious risk of bias and very serious inconsistency (Supplementary Materials, Appendix 4 F4). Aerobic exercise combined with other effective treatments had the highest probability of being the optimal therapy (81.3%), followed by pharmacological or psychological interventions (75.3%) and aerobic exercise plus resistance training (63.9%) (**Table 3**).

##### 3.3.2.5 High-density lipoprotein cholesterol

Of the 32 randomized controlled trials included, 14 reported high-density lipoprotein cholesterol as an outcome measure (32, 41, 45–49, 53–55, 57–60). The specific network diagram is shown in Figure (**Supplementary Materials, Appendix 1 F5**). The relative effects of different interventions on HDL-C in obese patients with T2DM are presented in (**Supplementary Materials, Appendix 2 F5**). Meta-analysis revealed that exercise significantly improved total intake compared to the control group (MD = 0.12, 95% CI [0.05, 0.18], I²=80). The certainty of evidence was low, downgraded due to serious risk of bias and very serious inconsistency (Supplementary Materials, Appendix 4 F5). Network meta-analysis results indicate that resistance training combined with other effective therapies (SMD = -0.33, 95% CI [-0.56, -0.11]), pharmacological or psychological interventions (SMD = -0.23, 95% CI [-0.31, -0.15]), aerobic exercise combined with other effective treatments (SMD = -0.38, 95% CI [-0.47, -0.28]), and resistance training alone (SMD = -0.13, 95% CI [-0.20, -0.06]) were more effective than the control group. The probability of resistance training combined with other effective treatments being the optimal treatment group was highest (97.5%), followed by the pharmacological or psychological treatment group (90.5%) and the resistance training alone group (77.8%) (**Table 3**).

##### 3.3.2.6 HOMA-IR

Of the 32 randomized controlled trials included, 15 reported HOMA-IR as an outcome measure (27, 32, 34, 35, 38, 42, 44–46, 48, 49, 53, 54, 56, 59). The specific network diagram is shown in Figure (**Supplementary Materials, Appendix 1 F6**). The relative effects of different interventions on HOMA-IR in obese patients with T2DM are shown in (**Supplementary Materials, Appendix 2 F6**). Meta-analysis revealed that, compared to the control group, exercise significantly improved total energy intake in obese patients with type 2 diabetes (MD = -0.92, 95% CI [-1.73, -0.10], I² = 98). The certainty of evidence was low, downgraded due to very serious inconsistency and not serious risk of bias (Supplementary Materials, Appendix 4 F6). Network meta-analysis results indicated that aerobic exercise combined with other effective treatments (SMD = -1.91, 95% CI [-2.82, -1.00]), aerobic plus resistance training (SMD = -1.02, 95% CI [-1.90, -0.14]), pharmacological or psychological therapy (SMD = -1.06, 95% CI [-2.10, -0.01]), and aerobic exercise alone (SMD = -0.78, 95% CI [-1.40, -0.16]) were more effective than the control group. Aerobic exercise combined with other effective treatments had the highest probability of being the optimal treatment (92.6%), followed by the walking or jogging group (63.0%) and the pharmacological or psychological therapy group (59.4%) (**Table 3**).

#### 3.3.3 Secondary outcome measures

Secondary outcomes included indicators with lower reporting frequency (<10 RCTs) and moderate evidence grade, which are indirectly related to the core research objectives or have relatively heterogeneous measurement methods, including interleukin IL-6, total energy intake, insulin level, and lean body mass. These indicators supplement the main outcomes to comprehensively evaluate the effects of exercise interventions, but their statistical power and evidence strength are lower than those of the main outcomes.

##### 3.3.3.1 Interleukin IL-6

Of the 32 randomized controlled trials included, 3 reported interleukin IL-6 as an outcome measure (43, 52, 56). The specific network diagram is shown in Figure (**Supplementary Materials, Appendix 1 F7**). The relative effects of different interventions on interleukin-6 (IL-6) in obese patients with T2DM are shown in (**Supplementary Materials, Appendix 2 F7**). Meta-analysis revealed that exercise significantly improved total energy intake in obese patients with T2DM compared to the control group (MD = -3.12, 95% CI [-6.22, -0.01], I² = 91). The certainty of evidence was very low, downgraded due to serious risk of bias and very serious inconsistency (**Supplementary Materials, Appendix 4 F7**). The network meta-analysis results showed that aerobic exercise combined with other effective treatments (SMD = -4.36, 95% CI [-5.84, -2.88]), aerobic exercise plus resistance training combined with other effective treatments (SMD = -2.05, 95% CI [-3.37, -0.72]), aerobic plus resistance combined with other effective treatments (SMD = -2.05, 95% CI [-3.37, -0.72]), aerobic plus resistance alone (SMD = -1.54, 95% CI [-2.25, -0.83]), and aerobic alone (SMD = -1.32, 95% CI [-2.22, -0.42]) were more effective than the control group. Aerobic exercise combined with other effective treatments had the highest probability (99.9%) of being the optimal treatment, followed by aerobic exercise plus resistance training combined with other effective treatments (72.4%) and aerobic exercise plus resistance training alone (58.4%) (**Table 3**).

##### 3.3.3.2 Total intake

Of the 32 randomized controlled trials included, 8 reported total intake as an outcome measure (37, 39, 43, 49, 52, 53, 58, 62). The specific network diagram is shown in Figure (**Supplementary Materials, Appendix 1 F8**). Meta-analysis revealed that compared with the control group, exercise intervention did not significantly improve total energy intake in patients with T2DM and obesity (MD = 7.91, 95% CI [−128.43, 144.26], I² = 89). The certainty of evidence was very low, downgraded due to serious risk of bias and very serious inconsistency (**Table 3**).

##### 3.3.3.3 Insulin

Of the 32 randomized controlled trials included, 8 reported insulin as an outcome measure (36, 38, 44, 45, 49, 54, 56, 59). The specific network diagram is shown in Figure (**Supplementary Materials, Appendix 1 F9**). The relative effects of different interventions on insulin levels in obese patients with T2DM are shown in (**Supplementary Materials, Appendix 2 F8**). Meta-analysis revealed that, compared to the control group, exercise significantly improved total caloric intake in obese patients with type 2 diabetes (MD = -1.16, 95% CI [-2.14, -0.18], I² = 58). The certainty of evidence was low, downgraded due to serious risk of bias and serious inconsistency (**Supplementary Materials, Appendix 4 F9**). Walking and jogging had the highest probability of being optimal treatments (90.9%), followed by aerobic combined with other effective treatments (69.6%) and aerobic plus resistance training (64.4%) (**Table 3**).

##### 3.3.3.4 Lean body mass

Of the 32 randomized controlled trials included, 8 reported lean body mass as an outcome measure (27, 40, 41, 47, 49, 56, 57, 62). The specific network diagram is shown in Figure (**Supplementary Materials, Appendix 1 F10**). The relative effects of different interventions on fat-free mass in patients with T2DM and obesity are presented in Table (**Supplementary Materials, Appendix 2 F9**). Meta-analysis revealed that exercise significantly improved total energy intake in patients with type 2 diabetes and obesity compared to the control group (MD = 0.24, 95% CI [0.04, 0.44], I²=0). The certainty of evidence was moderate, with no downgrades required, as there was no serious risk of bias, inconsistency, indirectness, or imprecision (**Supplementary Materials, Appendix 4 F10**). Mind-body exercise had the highest probability of being the optimal treatment (93.7%), followed by resistance training combined with other effective treatments (61.1%) and aerobic plus resistance training (54.3%) (**Table 3**).

### 3.4 Publication offset

Visual inspection of the comparison-adjusted funnel plots revealed no apparent asymmetry, suggesting no significant publication bias (**Supplementary Materials, Appendix 5 F1–10**). Visual examination of these plots showed that most data points were relatively symmetrically distributed around the vertical midline (centered on the pooled effect for a specific comparison), with no marked asymmetry observed across different intervention comparisons. Although minor dispersion of data points was observed in individual plots (e.g., extreme effect sizes in some comparisons), no consistent asymmetric trend indicative of substantial publication bias was detected.

## 4 Discussion

### 4.1 Conclusions, summary, and interpretation

Obesity is closely associated with the high prevalence of type 2 diabetes, with unhealthy diets and a sedentary lifestyle being the primary risk factors. The pathophysiological process of obesity complicated by type 2 diabetes is characterized by chronic low-grade inflammation, impaired pancreatic β-cell function, and insulin resistance, which in turn lead to disorders of glucose and lipid metabolism (63). Excessive accumulation of visceral fat disrupts the normal physiological functions of adipose tissue, stimulating the secretion of pro-inflammatory factors such as interleukin-6 (IL-6) and tumor necrosis factor-α (TNF-α), which trigger a systemic inflammatory response and impair insulin signaling pathways (64, 65). Persistent insulin resistance further exacerbates hyperglycemia and dyslipidemia, manifesting as elevated triglycerides and dysregulation of lipoprotein metabolism (66, 67). A sedentary lifestyle and an unbalanced diet continuously exacerbate this pathological vicious cycle, whereas exercise can interrupt this pathological pathway at multiple stages.

Different exercise modalities act on distinct targets in adipose tissue, skeletal muscle, and inflammatory regulation, resulting in varying metabolic improvements. Combining aerobic and resistance training simultaneously activates both lipolysis and skeletal muscle synthesis pathways, offering the most optimal overall regulatory effect. Some existing meta-analyses suggest that exercise has only a minimal effect on improving blood lipid levels in obese individuals with type 2 diabetes, which contradicts the conclusions of this study (68, 69). The differing effects of various exercise types on blood lipids, insulin resistance, and inflammation essentially stem from differences in their mechanisms of action: aerobic exercise combined with adjunctive interventions tends to focus more on regulating cholesterol levels; resistance training excels at increasing HDL-C; and combined intervention protocols demonstrate a more pronounced effect on regulating triglycerides; aerobic exercise combined with adjunctive measures possesses unique advantages in alleviating systemic inflammation and improving insulin resistance. Existing research has confirmed that aerobic or combined training can effectively suppress chronic low-grade inflammation, whereas high-intensity interval training alone struggles to reduce key inflammatory markers (70).

The key reason for the remarkable effects of aerobic and resistance training is the physiological synergistic effects between the two in terms of energy metabolism and body composition regulation (71). Aerobic exercise accelerates whole-body fat oxidation and increases total energy expenditure, while resistance training promotes skeletal muscle anabolism; the combination of these two types of training can synergistically improve physical performance and metabolic homeostasis. At the same time, exercise helps maintain and increase lean body mass by regulating neuroendocrine functions and enhancing exercise adherence (72). The differential responses of various lipoproteins to exercise stem from distinct adaptive mechanisms: adequate moderate-to-high-intensity aerobic exercise can increase lipoprotein lipase activity and energy expenditure, thereby lowering levels of low-density lipoprotein cholesterol (LDL-C) and triglycerides (71); resistance training promotes skeletal muscle mass growth and induces specific changes in lipoprotein metabolic flexibility, resulting in a more pronounced effect on raising HDL-C levels.

The role of exercise in improving insulin resistance is closely linked to its anti-inflammatory mechanisms. Moderate-intensity aerobic exercise can suppress IL-6 secretion from visceral fat and alleviate chronic, low-grade systemic inflammation, while avoiding the metabolic stress associated with high-intensity exercise and preventing transient elevations in TNF-α and IL-6 (73). Reduced inflammation levels can resolve inflammation-mediated blockages in insulin signaling and accelerate the efficiency of glucose uptake and utilization by skeletal muscle (74). In addition, combining aerobic and resistance training can further enhance the oxidative capacity of skeletal muscle mitochondria, thereby maintaining basal metabolic rate and overall metabolic stability over the long term (75).

This study incorporated visceral fat distribution, lipid metabolism levels, and inflammatory markers into a unified analytical framework and found that the various metabolic improvements brought about by exercise do not occur independently but rather constitute a continuous, synergistic set of physiological regulatory pathways. Exercise reduces visceral fat accumulation, attenuates adipose tissue-mediated systemic inflammation, restores impaired insulin signaling, and enhances the body’s efficiency in utilizing glucose and fatty acids; this series of cascading physiological changes simultaneously optimizes body weight, lipid profile, and systemic inflammation levels, and further improves insulin sensitivity, lipoprotein lipase activity, and fatty acid oxidation capacity (76, 77). It is worth noting that the metabolic benefits of exercise are independent of changes in total energy intake; exercise can improve skeletal muscle mitochondrial function and increase lean body mass without the need to restrict caloric intake, indicating that the metabolic benefits of exercise extend far beyond a simple increase in caloric expenditure.

In summary, exercise can intervene in the pathological processes of inflammation and insulin resistance induced by visceral fat accumulation, achieving holistic regulation of systemic metabolism, and providing a physiological theoretical basis for the comprehensive clinical management of patients with obesity and type 2 diabetes. Combined aerobic and resistance training can simultaneously activate both the fat breakdown and skeletal muscle synthesis pathways, comprehensively improving metabolic disorders in the body, and is therefore suitable as the preferred exercise intervention strategy for this high-risk population.

Meanwhile differences in the evidence from various clinical studies can be distinguished at the research design level: Longitudinal intervention cohort studies have shown that maintaining a consistent exercise routine over the long term can gradually regulate lipid metabolism and systemic chronic inflammation in patients with obesity and type 2 diabetes. However, such observational studies find it difficult to strictly control for confounding variables such as diet, antidiabetic medications, and baseline body weight, making it impossible to isolate the independent metabolic effects of exercise; Cross-sectional studies rely solely on data collected at a single time point; their results can only reflect trends in the association between daily physical activity and glucose and lipid markers, but cannot establish a clear causal relationship between the two. This study conducted a network meta-analysis incorporating all randomized controlled trials (RCTs) that met the inclusion criteria. All included studies were trials with intervention and control groups; observational studies, such as cross-sectional and longitudinal cohort studies, were excluded throughout the process. The pooled results, based on standardized data from controlled interventions, confirm that standardized exercise programs can independently improve metabolic disorders and suppress low-grade inflammation in patients.

### 4.2 Strengths and limitations of this study

This study possesses the following strengths: (1) Focusing on patients with T2DM and obesity, it systematically integrates the effects of different exercise interventions on abdominal fat, lipid metabolism, and inflammatory factors. Through network meta-analysis, it compares and ranks various interventions, providing more comprehensive evidence-based support for developing clinical exercise prescriptions. (2) This study exclusively included randomized controlled trials, featuring a rigorous overall design that enhances the reliability and scientific validity of its conclusions.

This study also has certain limitations: (1) The number of included studies reporting consistent subgroup-level data (e.g., by obesity severity, diabetes duration, or baseline metabolic status) was insufficient to perform meaningful subgroup analyses. This prevented us from exploring whether the effects of exercise interventions varied across these patient characteristics. (2) No subgroup analysis was performed in the network meta-analysis, and potential biases may be induced by differences in patient characteristics such as gender, which may affect the accuracy and generalizability of the results. (3) Effect sizes for some outcome measures required conversion from raw data or indirect estimation, potentially introducing estimation bias. (4) Literature search was limited to studies published in Chinese and English, potentially introducing language bias. (5) Most included studies inadequately reported potential confounders (e.g., medication use, disease severity, and intervention adherence), which may affect the precision of pooled effect sizes and interpretation of results.(6) Only randomized controlled trials were included, with cross-sectional and longitudinal observational studies excluded, which may reduce the external generalizability of our research findings.

### 4.3 Implications for clinical practice and future directions

The comprehensive evidence from this study suggests that multiple exercise intervention strategies can improve abdominal fat distribution, lipid metabolism status, and inflammatory factor levels in obese patients with T2DM, relatively independent of changes in total energy intake. Among these, aerobic combined with resistance training, especially when integrated into comprehensive programs alongside other effective interventions, demonstrated more consistent and superior outcomes. In clinical practice, healthcare professionals should prioritize structured, comprehensive exercise intervention strategies tailored to patients’ individual characteristics and metabolic profiles to maximize exercise’s role in metabolic management. While this study confirms the efficacy of various exercise interventions in improving relevant metabolic indicators, future research requires larger-scale, rigorously designed multicenter randomized controlled trials to further clarify optimal exercise combinations and their long-term intervention effects.

## 5 Conclusion

Notably, exercise interventions can improve fat accumulation, lipid metabolism, insulin resistance, and inflammatory markers in patients with T2DM and obesity without the need for strict caloric intake control. Our findings confirmed no significant changes in total energy intake among participants following exercise interventions, yet metabolic and inflammatory improvements remained substantial. Combining aerobic exercise with resistance training yields the most favorable comprehensive intervention effects. Therefore, healthcare providers should be encouraged to incorporate these interventions as part of routine clinical management for this population, as they offer a feasible and sustainable approach independent of rigorous dietary restriction. Given the heterogeneity in metabolic dysfunction and treatment response among obese T2DM patients, future high-quality, large-scale randomized controlled trials should incorporate phenotype-specific and stratified analyses. Such targeted research is crucial for validating existing findings and developing more personalized, effective exercise intervention protocols for long-term metabolic health.

## Data Availability

The original contributions presented in the study are included in the article/**Supplementary Material**. Further inquiries can be directed to the corresponding author.
