# Supplementary material for: Exercise modalities for adults with type 2 diabetes mellitus and obesity: comparative effects on visceral fat, metabolism, and inflammatory markers from a network meta-analysis
Source: Front Immunol. 2026 Aug 25;17:1825473. doi: 10.3389/fimmu.2026.1825473 (PMC13549836; doi:10.3389/fimmu.2026.1825473)
Supplement: Supplementary file 1 [file DataSheet1.pdf]

Directory

Table S1 PRISMA 2020 Checklist 2

Table S2 specific search strategies 6

Table S3 PICOS inclusion criteria 10

Appendix 1 12

Appendix 2 16

Appendix 3 20

Appendix 4 25

Appendix 5 29

Appendix 6 34

Appendix 7 40

Appendix 8 45

Table S1 PRISMA 2020 Checklist

Section and Topic	Item #	Checklist item	Location where item is reported
**TITLE**			
Title	1	Identify the report as a systematic review.	1
**ABSTRACT**			
Abstract	2	See the PRISMA 2020 for Abstracts checklist.	1-2
**INTRODUCTION**			
Rationale	3	Describe the rationale for the review in the context of existing knowledge.	2-3
Objectives	4	Provide an explicit statement of the objective(s) or question(s) the review addresses.	3-4
**METHODS**			
Eligibility criteria	5	Specify the inclusion and exclusion criteria for the review and how studies were grouped for the syntheses.	4-5
Information sources	6	Specify all databases, registers, websites, organisations, reference lists and other sources searched or consulted to identify studies. Specify the date when each source was last searched or consulted.	4-5
Search strategy	7	Present the full search strategies for all databases, registers and websites, including any filters and limits used.	4,S1
Selection process	8	Specify the methods used to decide whether a study met the inclusion criteria of the review, including how many reviewers screened each record and each report retrieved, whether they worked independently, and if applicable, details of automation tools used in the process.	5
Data collection process	9	Specify the methods used to collect data from reports, including how many reviewers collected data from each report, whether they worked independently, any processes for obtaining or confirming data from study investigators, and if applicable, details of automation tools used in the process.	5
Data items	10a	List and define all outcomes for which data were sought. Specify whether all results that were compatible with each outcome domain in each study were sought (e.g. for all measures, time points, analyses), and if not, the methods used to decide which results to collect.	4-5
	10b	List and define all other variables for which data were sought (e.g. participant and intervention characteristics, funding sources). Describe any assumptions made about any missing or unclear information.	6-7
Study risk of bias	11	Specify the methods used to assess risk of bias in the included studies, including details of the tool(s) used, how many reviewers assessed each	5-6

Section and Topic	Item #	Checklist item	Location where item is reported
assessment		study and whether they worked independently, and if applicable, details of automation tools used in the process.	
Effect measures	12	Specify for each outcome the effect measure(s) (e.g. risk ratio, mean difference) used in the synthesis or presentation of results.	5-6
Synthesis methods	13a	Describe the processes used to decide which studies were eligible for each synthesis (e.g. tabulating the study intervention characteristics and comparing against the planned groups for each synthesis (item #5)).	4-5,8-12
	13b	Describe any methods required to prepare the data for presentation or synthesis, such as handling of missing summary statistics, or data conversions.	5-7
	13c	Describe any methods used to tabulate or visually display results of individual studies and syntheses.	5-7
	13d	Describe any methods used to synthesize results and provide a rationale for the choice(s). If meta-analysis was performed, describe the model(s), method(s) to identify the presence and extent of statistical heterogeneity, and software package(s) used.	5-7
	13e	Describe any methods used to explore possible causes of heterogeneity among study results (e.g. subgroup analysis, meta-regression).	6-7
	13f	Describe any sensitivity analyses conducted to assess robustness of the synthesized results.	6-7,8-9
Reporting bias assessment	14	Describe any methods used to assess risk of bias due to missing results in a synthesis (arising from reporting biases).	6-7,12
Certainty assessment	15	Describe any methods used to assess certainty (or confidence) in the body of evidence for an outcome.	6-7,12
**RESULTS**			
Study selection	16a	Describe the results of the search and selection process, from the number of records identified in the search to the number of studies included in the review, ideally using a flow diagram.	7-8
	16b	Cite studies that might appear to meet the inclusion criteria, but which were excluded, and explain why they were excluded.	8
Study characteristics	17	Cite each included study and present its characteristics.	4-5
Risk of bias in studies	18	Present assessments of risk of bias for each included study.	8

Section and Topic	Item #	Checklist item	Location where item is reported
Results of individual studies	19	For all outcomes, present, for each study: (a) summary statistics for each group (where appropriate) and (b) an effect estimate and its precision (e.g. confidence/credible interval), ideally using structured tables or plots.	8-12
Results of syntheses	20a	For each synthesis, briefly summarise the characteristics and risk of bias among contributing studies.	8-9
	20b	Present results of all statistical syntheses conducted. If meta-analysis was done, present for each the summary estimate and its precision (e.g. confidence/credible interval) and measures of statistical heterogeneity. If comparing groups, describe the direction of the effect.	8-12
	20c	Present results of all investigations of possible causes of heterogeneity among study results.	12
	20d	Present results of all sensitivity analyses conducted to assess the robustness of the synthesized results.	8-12
Reporting biases	21	Present assessments of risk of bias due to missing results (arising from reporting biases) for each synthesis assessed.	12
Certainty of evidence	22	Present assessments of certainty (or confidence) in the body of evidence for each outcome assessed.	12-18
**DISCUSSION**			
Discussion	23a	Provide a general interpretation of the results in the context of other evidence.	12-15
	23b	Discuss any limitations of the evidence included in the review.	15
	23c	Discuss any limitations of the review processes used.	15
	23d	Discuss implications of the results for practice, policy, and future research.	15-16
**OTHER INFORMATION**			
Registration and protocol	24a	Provide registration information for the review, including register name and registration number, or state that the review was not registered.	4
	24b	Indicate where the review protocol can be accessed, or state that a protocol was not prepared.	4
	24c	Describe and explain any amendments to information provided at registration or in the protocol.	4
Support	25	Describe sources of financial or non-financial support for the review, and the role of the funders or sponsors in the review.	Appendix
Competing interests	26	Declare any competing interests of review authors.	Appendix

Section and Topic	Item #	Checklist item	Location where item is reported
Availability of data, code and other materials	27	Report which of the following are publicly available and where they can be found: template data collection forms; data extracted from included studies; data used for all analyses; analytic code; any other materials used in the review.	Appendix

From: Page MJ, McKenzie JE, Bossuyt PM, Boutron I, Hoffmann TC, Mulrow CD, et al. The PRISMA 2020 statement: an updated guideline for reporting systematic reviews. BMJ 2021;372:n71. doi: 10.1136/bmj.n71

For more information, visit: http://www.prisma-statement.org/

Table S2 specific search strategies

**pubmed**:<br>(((((("Exercise"[Mesh]) OR ((((((((((((((((((((((((Exercises[Title/Abstract]) OR (Exercise, Physical[Title/Abstract])) OR (Exercises, Physical[Title/Abstract])) OR (Physical Exercise[Title/Abstract])) OR (Physical Exercises[Title/Abstract])) OR (Exercise, Aerobic[Title/Abstract])) OR (Aerobic Exercise[Title/Abstract])) OR (Aerobic Exercises[Title/Abstract])) OR (Exercises, Aerobic[Title/Abstract])) OR (Exercise, Isometric[Title/Abstract])) OR (Exercises, Isometric[Title/Abstract])) OR (Isometric Exercises[Title/Abstract])) OR (Isometric Exercise[Title/Abstract])) OR (Acute Exercise[Title/Abstract])) OR (Acute Exercises[Title/Abstract])) OR (Exercise, Acute[Title/Abstract])) OR (Exercises, Acute[Title/Abstract])) OR (Exercise Training[Title/Abstract])) OR (Exercise Trainings[Title/Abstract])) OR (Training, Exercise[Title/Abstract])) OR (Trainings, Exercise[Title/Abstract])) OR (Physical Activity[Title/Abstract])) OR (Activities, Physical[Title/Abstract])) OR (Activity, Physical[Title/Abstract])) OR (Physical Activities[Title/Abstract]))) OR (("Sports"[Mesh]) OR (((Sport[Title/Abstract]) OR (Athletics[Title/Abstract])) OR (Athletic[Title/Abstract])))) AND (("Obesity"[Mesh]) OR ((obese[Title/Abstract]) OR (overweight[Title/Abstract])))) AND (("Diabetes Mellitus, Type 2"[Mesh]) OR ((((((((((((((((((((((((((((((Diabetes Mellitus, Adult-Onset[Title/Abstract]) OR (Adult-Onset Diabetes Mellitus[Title/Abstract])) OR (Diabetes Mellitus, Adult Onset[Title/Abstract])) OR (Diabetes Mellitus, Ketosis-Resistant[Title/Abstract])) OR (Diabetes Mellitus, Ketosis Resistant[Title/Abstract])) OR (Ketosis-Resistant Diabetes Mellitus[Title/Abstract])) OR (NIDDM[Title/Abstract])) OR (Diabetes Mellitus, Maturity-Onset[Title/Abstract])) OR (Diabetes Mellitus, Maturity Onset[Title/Abstract])) OR (Diabetes Mellitus, Non Insulin Dependent[Title/Abstract])) OR (Diabetes Mellitus, Non-Insulin-Dependent[Title/Abstract])) OR (Non-Insulin-Dependent Diabetes Mellitus[Title/Abstract])) OR (Diabetes Mellitus, Noninsulin Dependent[Title/Abstract])) OR (Diabetes Mellitus, Noninsulin-Dependent[Title/Abstract])) OR (Diabetes Mellitus, Slow-Onset[Title/Abstract])) OR (Diabetes Mellitus, Slow Onset[Title/Abstract])) OR (Slow-Onset Diabetes Mellitus[Title/Abstract])) OR (Diabetes Mellitus, Stable[Title/Abstract])) OR (Stable Diabetes Mellitus[Title/Abstract])) OR (Diabetes Mellitus, Type II[Title/Abstract])) OR (Maturity-Onset Diabetes[Title/Abstract])) OR (Diabetes, Maturity-Onset[Title/Abstract])) OR (Maturity Onset Diabetes[Title/Abstract])) OR (Maturity-Onset Diabetes Mellitus[Title/Abstract])) OR (Maturity Onset Diabetes Mellitus[Title/Abstract])) OR (MODY (Maturity-onset diabetes of the young[Title/Abstract]))) OR (Noninsulin-Dependent Diabetes Mellitus[Title/Abstract])) OR (Noninsulin Dependent Diabetes Mellitus[Title/Abstract])) OR (Type 2 Diabetes[Title/Abstract])) OR (Diabetes, Type 2[Title/Abstract])) OR (Type 2 Diabetes Mellitus[Title/Abstract]))))) AND (("Adult"[Mesh]) OR (Adults[Title/Abstract])
**WOS**:<br>TS=(exercise OR Exercises OR Exercise, Physical OR Exercises, Physical OR Physical Exercise OR Physical Exercises OR Exercise, Aerobic OR Aerobic Exercise OR Aerobic Exercises OR Exercises, Aerobic OR Exercise, Isometric OR Exercises, Isometric OR Isometric Exercises OR Isometric Exercise OR Acute Exercise OR Acute Exercises OR Exercise, Acute OR Exercises, Acute OR Exercise Training OR Exercise Trainings OR Training, Exercise OR Trainings, Exercise OR Physical Activity OR Activities, Physical OR Activity, Physical OR Physical Activities)<br>TS=(sports OR Sport OR Athletics OR Athletic)<br>TS=(obesity OR obese OR overweight)<br>TS=(Diabetes Mellitus, Type 2 OR Diabetes Mellitus, Adult-Onset OR Adult-Onset Diabetes Mellitus OR Diabetes Mellitus, Adult Onset OR Diabetes Mellitus, Ketosis-Resistant OR Diabetes Mellitus, Ketosis Resistant

OR Ketosis-Resistant Diabetes Mellitus OR NIDDM OR Diabetes Mellitus, Maturity-Onset OR Diabetes Mellitus, Maturity Onset OR Diabetes Mellitus, Non Insulin Dependent OR Diabetes Mellitus, Non-Insulin-Dependent OR Non-Insulin-Dependent Diabetes Mellitus OR Diabetes Mellitus, Noninsulin Dependent OR Diabetes Mellitus, Noninsulin-Dependent OR Diabetes Mellitus, Slow-Onset OR Diabetes Mellitus, Slow Onset OR Slow-Onset Diabetes Mellitus OR Diabetes Mellitus, Stable OR Stable Diabetes Mellitus OR Diabetes Mellitus, Type II OR Maturity-Onset Diabetes OR Diabetes, Maturity-Onset OR Maturity Onset Diabetes OR Maturity-Onset Diabetes Mellitus OR Maturity Onset Diabetes Mellitus OR MODY (Maturity-onset diabetes of the young) OR Noninsulin-Dependent Diabetes Mellitus OR Noninsulin Dependent Diabetes Mellitus OR Type 2 Diabetes OR Diabetes, Type 2 OR Type 2 Diabetes Mellitus)

TS=(adults OR adult)

cochrane:

exercise

(Exercises):ab,ti,kw OR (Exercise, Physical):ab,ti,kw OR (Exercises, Physical):ab,ti,kw OR (Physical Exercise):ab,ti,kw OR (Physical Exercises):ab,ti,kw OR (Exercise, Aerobic):ab,ti,kw OR (Aerobic Exercise):ab,ti,kw OR (Aerobic Exercises):ab,ti,kw OR (Exercises, Aerobic):ab,ti,kw OR (Exercise, Isometric):ab,ti,kw OR (Exercises, Isometric):ab,ti,kw OR (Isometric Exercises):ab,ti,kw OR (Isometric Exercise):ab,ti,kw OR (Acute Exercise):ab,ti,kw OR (Acute Exercises):ab,ti,kw OR (Exercise, Acute):ab,ti,kw OR (Exercises, Acute):ab,ti,kw OR (Exercise Training):ab,ti,kw OR (Exercise Trainings):ab,ti,kw OR (Training, Exercise):ab,ti,kw OR (Trainings, Exercise):ab,ti,kw OR (Physical Activity):ab,ti,kw OR (Activities, Physical):ab,ti,kw OR (Activity, Physical):ab,ti,kw OR (Physical Activities):ab,ti,kw

sports

(Sport):ab,ti,kw OR (Athletics):ab,ti,kw OR (Athletic):ab,ti,kw

obesity

(obese):ab,ti,kw OR (overweight):ab,ti,kw

Diabetes Mellitus, Type 2

(Diabetes Mellitus, Adult-Onset):ab,ti,kw OR (Adult-Onset Diabetes Mellitus):ab,ti,kw OR (Diabetes Mellitus, Adult Onset):ab,ti,kw OR (Diabetes Mellitus, Ketosis-Resistant):ab,ti,kw OR (Diabetes Mellitus, Ketosis Resistant):ab,ti,kw OR (Ketosis-Resistant Diabetes Mellitus):ab,ti,kw OR (NIDDM):ab,ti,kw OR (Diabetes Mellitus, Maturity-Onset):ab,ti,kw OR (Diabetes Mellitus, Maturity Onset):ab,ti,kw OR (Diabetes Mellitus, Non Insulin Dependent):ab,ti,kw OR (Diabetes Mellitus, Non-Insulin-Dependent):ab,ti,kw OR (Non-Insulin-Dependent Diabetes Mellitus):ab,ti,kw OR (Diabetes Mellitus, Noninsulin Dependent):ab,ti,kw OR (Diabetes Mellitus, Noninsulin-Dependent):ab,ti,kw OR (Diabetes Mellitus, Slow-Onset):ab,ti,kw OR (Diabetes Mellitus, Slow Onset):ab,ti,kw OR (Slow-Onset Diabetes Mellitus):ab,ti,kw OR (Diabetes Mellitus, Stable):ab,ti,kw OR (Stable Diabetes Mellitus):ab,ti,kw OR (Diabetes Mellitus, Type II):ab,ti,kw OR (Maturity-Onset Diabetes):ab,ti,kw OR (Diabetes, Maturity-Onset):ab,ti,kw OR (Maturity Onset Diabetes):ab,ti,kw OR (Maturity-Onset Diabetes Mellitus):ab,ti,kw OR (Maturity Onset Diabetes Mellitus):ab,ti,kw OR (MODY (Maturity-onset diabetes of the young)):ab,ti,kw OR (Noninsulin-Dependent Diabetes Mellitus):ab,ti,kw OR (Noninsulin Dependent Diabetes

Mellitus):ab,ti,kw OR (Type 2 Diabetes):ab,ti,kw OR (Diabetes, Type 2):ab,ti,kw OR (Type 2 Diabetes Mellitus):ab,ti,kw<br>adult<br>(adults):ab,ti,kw
**embase:**<br>Exercises-'exercise'/exp OR 'exercise'<br>'Exercises':ab,ti OR 'Exercise, Physical':ab,ti OR 'Exercises, Physical':ab,ti OR 'Physical Exercise':ab,ti OR 'Physical Exercises':ab,ti OR 'Exercise, Aerobic':ab,ti OR 'Aerobic Exercise':ab,ti OR 'Aerobic Exercises':ab,ti OR 'Exercises, Aerobic':ab,ti OR 'Exercise, Isometric':ab,ti OR 'Exercises, Isometric':ab,ti OR 'Isometric Exercises':ab,ti OR 'Isometric Exercise':ab,ti OR 'Acute Exercise':ab,ti OR 'Acute Exercises':ab,ti OR 'Exercise, Acute':ab,ti OR 'Exercises, Acute':ab,ti OR 'Exercise Training':ab,ti OR 'Exercise Trainings':ab,ti OR 'Training, Exercise':ab,ti OR 'Trainings, Exercise':ab,ti OR 'Physical Activity':ab,ti OR 'Activities, Physical':ab,ti OR 'Activity, Physical':ab,ti OR 'Physical Activities':ab,ti<br>sport-'sport'/exp OR 'sport'<br>'Sports':ab,ti OR 'Athletics':ab,ti OR 'Athletic':ab,ti<br>obesity-'obesity'/exp OR 'obesity'<br>'obese':ab,ti OR 'overweight':ab,ti<br>Diabetes Mellitus, Type 2-('diabetes'/exp OR diabetes) AND mellitus, AND type AND ('2'/exp OR 2)<br>'Diabetes Mellitus, Adult-Onset':ab,ti OR 'Adult-Onset Diabetes Mellitus':ab,ti OR 'Diabetes Mellitus, Adult Onset':ab,ti OR 'Diabetes Mellitus, Ketosis-Resistant':ab,ti OR 'Diabetes Mellitus, Ketosis Resistant':ab,ti OR 'Ketosis-Resistant Diabetes Mellitus':ab,ti OR 'NIDDM':ab,ti OR 'Diabetes Mellitus, Maturity-Onset':ab,ti OR 'Diabetes Mellitus, Maturity Onset':ab,ti OR 'Diabetes Mellitus, Non Insulin Dependent':ab,ti OR 'Diabetes Mellitus, Non-Insulin-Dependent':ab,ti OR 'Non-Insulin-Dependent Diabetes Mellitus':ab,ti OR 'Diabetes Mellitus, Noninsulin Dependent':ab,ti OR 'Diabetes Mellitus, Noninsulin-Dependent':ab,ti OR 'Diabetes Mellitus, Slow-Onset':ab,ti OR 'Diabetes Mellitus, Slow Onset':ab,ti OR 'Slow-Onset Diabetes Mellitus':ab,ti OR 'Diabetes Mellitus, Stable':ab,ti OR 'Stable Diabetes Mellitus':ab,ti OR 'Diabetes Mellitus, Type II':ab,ti OR 'Maturity-Onset Diabetes':ab,ti OR 'Diabetes, Maturity-Onset':ab,ti OR 'Maturity Onset Diabetes':ab,ti OR 'Maturity-Onset Diabetes Mellitus':ab,ti OR 'Maturity Onset Diabetes Mellitus':ab,ti OR 'MODY (Maturity-onset diabetes of the young)':ab,ti OR 'Noninsulin-Dependent Diabetes Mellitus':ab,ti OR 'Noninsulin Dependent Diabetes Mellitus':ab,ti OR 'Type 2 Diabetes':ab,ti OR 'Diabetes, Type 2':ab,ti OR 'Type 2 Diabetes Mellitus':ab,ti<br>adult-'adult'/exp OR 'adult'<br>'adults':ab,ti

andomized AND controlled AND ('trial'/exp OR trial)<br>'andomized controlled trial':ab,ti OR 'andomized':ab,ti OR 'placebo':ab,ti OR 'RCT':ab,ti
scopus<br>"exercise" OR "Exercises" OR "Exercise, Physical" OR "Exercises, Physical" OR "Physical Exercise" OR "Physical Exercises" OR "Exercise, Aerobic" OR "Aerobic Exercise" OR "Aerobic Exercises" OR "Exercises, Aerobic" OR "Exercise, Isometric" OR "Exercises, Isometric" OR "Isometric Exercises" OR "Isometric Exercise" OR "Acute Exercise" OR "Acute Exercises" OR "Exercise, Acute" OR "Exercises, Acute" OR "Exercise Training" OR "Exercise Trainings" OR "Training, Exercise" OR "Trainings, Exercise" OR "Physical Activity" OR "Activities, Physical" OR "Activity, Physical" OR "Physical Activities" OR "sports" OR "Sport" OR "Athletics" OR "Athletic" AND "obesity" OR "obese" OR "overweight" AND "Diabetes Mellitus, Type 2" OR "Diabetes Mellitus, Adult-Onset" OR "Adult-Onset Diabetes Mellitus" OR "Diabetes Mellitus, Adult Onset" OR "Diabetes Mellitus, Ketosis-Resistant" OR "Diabetes Mellitus, Ketosis Resistant" OR "Ketosis-Resistant Diabetes Mellitus" OR "NIDDM" OR "Diabetes Mellitus, Maturity-Onset" OR "Diabetes Mellitus, Maturity Onset" OR "Diabetes Mellitus, Non Insulin Dependent" OR "Diabetes Mellitus, Non-Insulin-Dependent" OR "Non-Insulin-Dependent Diabetes Mellitus" OR "Diabetes Mellitus, Noninsulin Dependent" OR "Diabetes Mellitus, Noninsulin-Dependent" OR "Diabetes Mellitus, Slow-Onset" OR "Diabetes Mellitus, Slow Onset" OR "Slow-Onset Diabetes Mellitus" OR "Diabetes Mellitus, Stable" OR "Stable Diabetes Mellitus" OR "Diabetes Mellitus, Type II" OR "Maturity-Onset Diabetes" OR "Diabetes, Maturity-Onset" OR "Maturity Onset Diabetes" OR "Maturity-Onset Diabetes Mellitus" OR "Maturity Onset Diabetes Mellitus" OR "MODY (Maturity-onset diabetes of the young)" OR "Noninsulin-Dependent Diabetes Mellitus" OR "Noninsulin Dependent Diabetes Mellitus" OR "Type 2 Diabetes" OR "Diabetes, Type 2" OR "Type 2 Diabetes Mellitus" AND "adult" OR "adults" AND "andomized controlled trial" OR "andomized" OR "placebo" OR "RCT"

Table S3 PICOS inclusion criteria

Population:	Adult patients (≥18 years old) with clinically confirmed type 2 diabetes mellitus (T2DM) and overweight or obesity, regardless of gender, disease duration, or severity.
Intervention Measures:	Included interventions must incorporate at least one exercise-based component, including but not limited to: Aerobic exercise alone, Resistance training alone, Combined aerobic and resistance training, Aerobic or resistance training combined with other effective interventions (e.g., standard care or health management), Structured exercise formats such as mind-body exercise, walking, or jogging. Specific details regarding the content, format, and combination of various exercise interventions are outlined in the Intervention Classification Description see table 1.
Comparison:	The control group receives no exercise intervention, including routine care, health education, placebo intervention, or maintains their original lifestyle.
Outcomes:	Studies must report at least one of the following outcome measures with extractable data. Outcomes were classified into primary and secondary outcomes based on reporting frequency across included studies and clinical evidence grade, consistent with standards for network meta-analysis.<br>Primary outcomes were defined as indicators with high reporting frequency (reported in ≥12 RCTs) and high clinical relevance to the core research question, reflecting key pathological characteristics in patients with type 2 diabetes and obesity. These included:BMI, total cholesterol (TC), triglycerides (TG), low-density lipoprotein cholesterol (LDL-C), high-density lipoprotein cholesterol (HDL-C), HOMA-IR. Secondary outcomes were defined as indicators with lower reporting frequency (<12 RCTs) and moderate evidence grade, which were indirectly related to core metabolic and inflammatory improvements but provided supplementary descriptive information. These included inflammatory markers interleukin-6， Total intake， Insulin， Lean body mass.
Study Design:	Include randomized controlled trials (RCTs).

	Exclusion criteria include: ① Literature where full-text access is unavailable (e.g., only titles or abstracts provided, with unsuccessful attempts to contact authors or corresponding authors); ② Studies published in languages other than Chinese or English; ③ Animal studies; ④ Books, conference proceedings, or registration protocols; ⑤ Studies focusing on patients with obesity and other chronic diseases, or on children, adolescents, or pregnant women; ⑥ Reviews or meta-analyses; ⑦ Studies with irrelevant research content.

Appendix 1

Primary Outcome

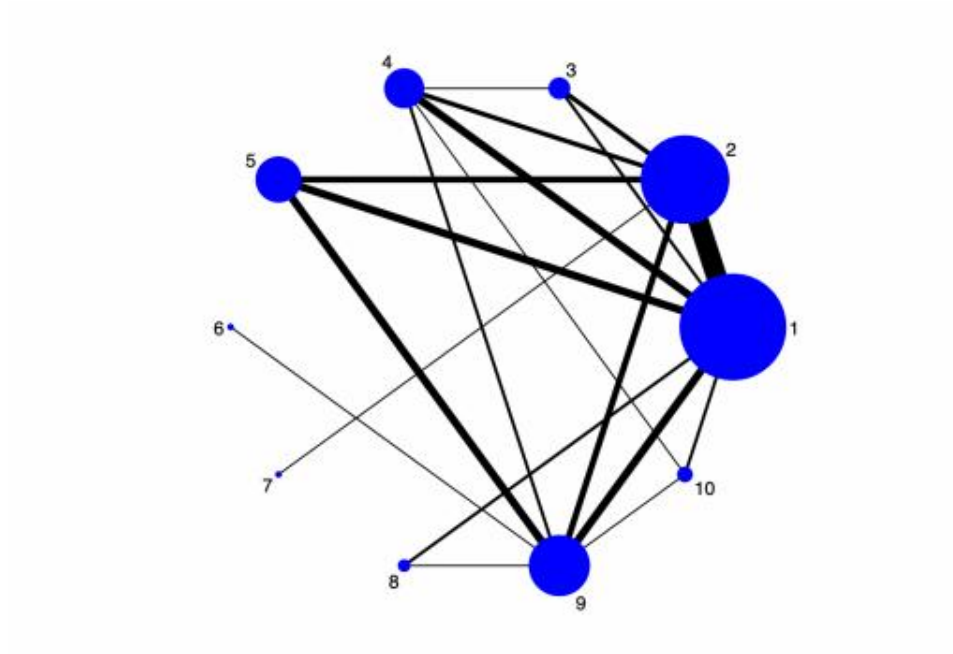

Note: Blank control group (1), Aerobic exercise only (2), Resistance exercise only(3), Aerobic plus resistance exercise (4), Aerobic exercise combined with other effective treatments (5), Resistance exercise combined with other effective treatments (6), Mind-body exercise group (7), Walking and jogging group (8), Pharmacological or psychological group(9), Aerobic exercise plus resistance exercise combined with other effective treatments(10).

F 1 Interventions in different ways for BMI network meta

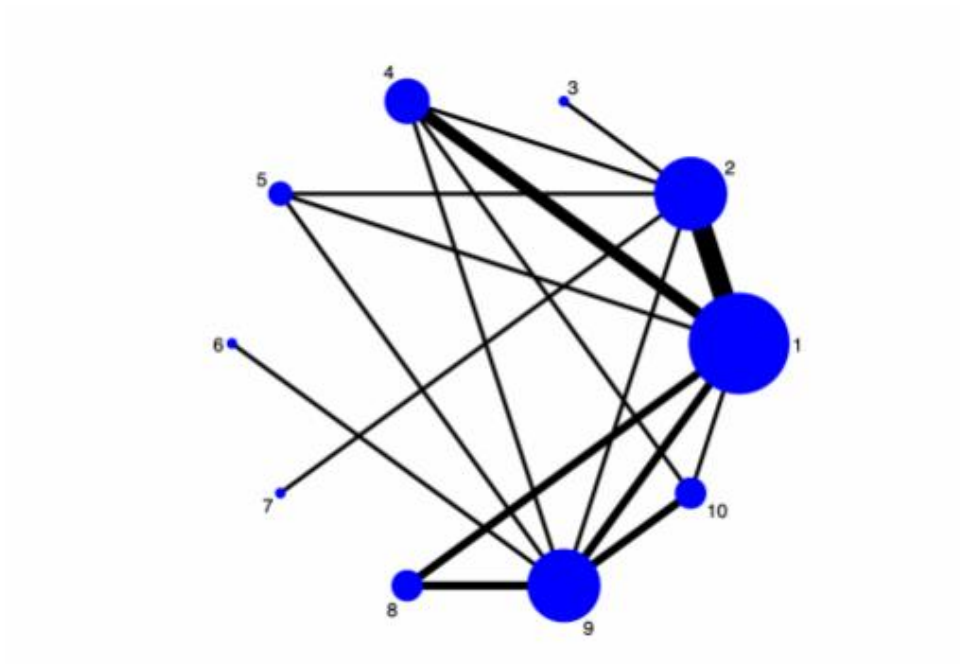

Note: Blank control group (1), Aerobic exercise only (2), Resistance exercise only(3), Aerobic plus resistance exercise (4), Aerobic exercise combined with other effective treatments (5), Resistance exercise combined with other effective treatments (6), Mind-body exercise group (7), Walking and jogging group (8), Pharmacological or psychological group(9), Aerobic exercise plus resistance exercise combined with other effective treatments(10).

F 2 Interventions in different ways for Total choleterol network meta

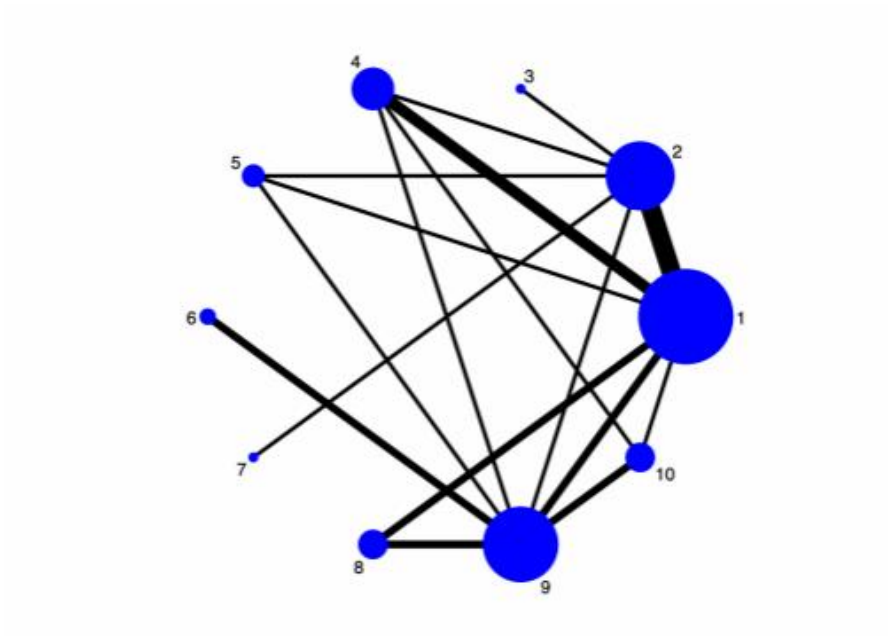

Note: Blank control group (1), Aerobic exercise only (2), Resistance exercise only(3), Aerobic plus resistance exercise (4), Aerobic exercise combined with other effective treatments (5), Resistance exercise combined with other effective treatments (6), Mind-body exercise group (7), Walking and jogging group (8), Pharmacological or psychological group(9), Aerobic exercise plus resistance exercise combined with other effective treatments(10).

F 3 Interventions in different ways for Triglycerides network meta

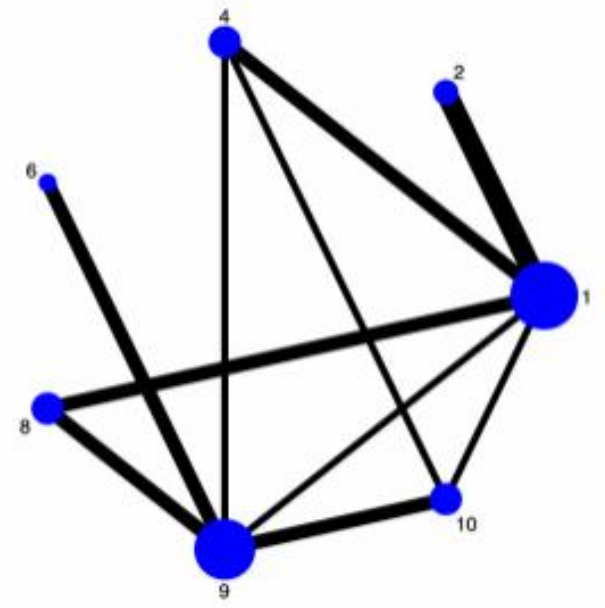

Note: Blank control group (1), Aerobic exercise only (2), Aerobic plus resistance exercise (4), Resistance exercise combined with other effective treatments (6), Walking and jogging group (8), Pharmacological or psychological group(9), Aerobic exercise plus resistance exercise combined with other effective treatments(10).

F 4 Interventions in different ways for LDL-C network meta

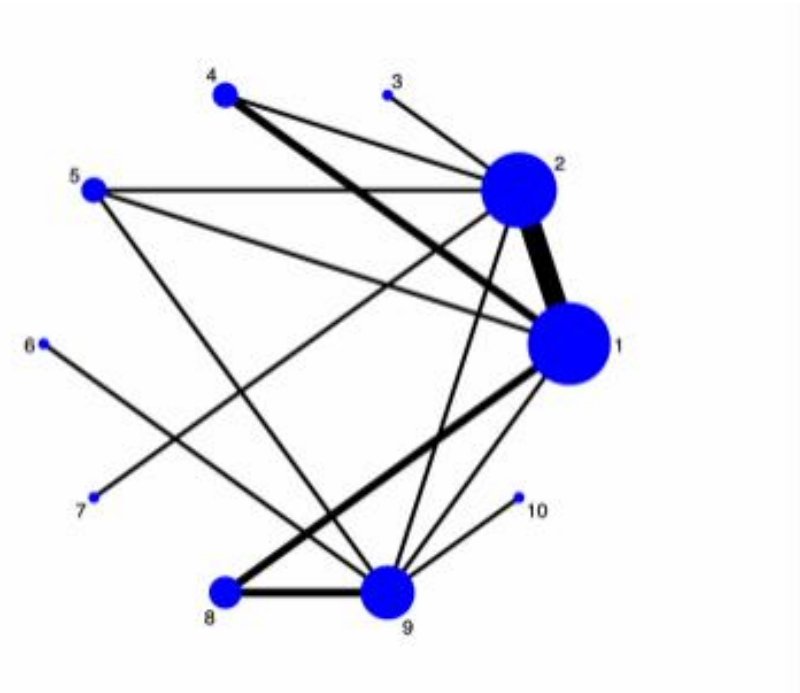

Note: Blank control group (1), Aerobic exercise only (2), Resistance exercise only(3), Aerobic plus resistance exercise (4), Aerobic exercise combined with other effective treatments (5), Resistance exercise combined with other effective treatments (6), Mind–body exercise group (7), Walking and jogging group (8), Pharmacological or psychological group(9), Aerobic exercise plus resistance exercise combined with other effective treatments(10).

F 5 Interventions in different ways for HDL-C network meta

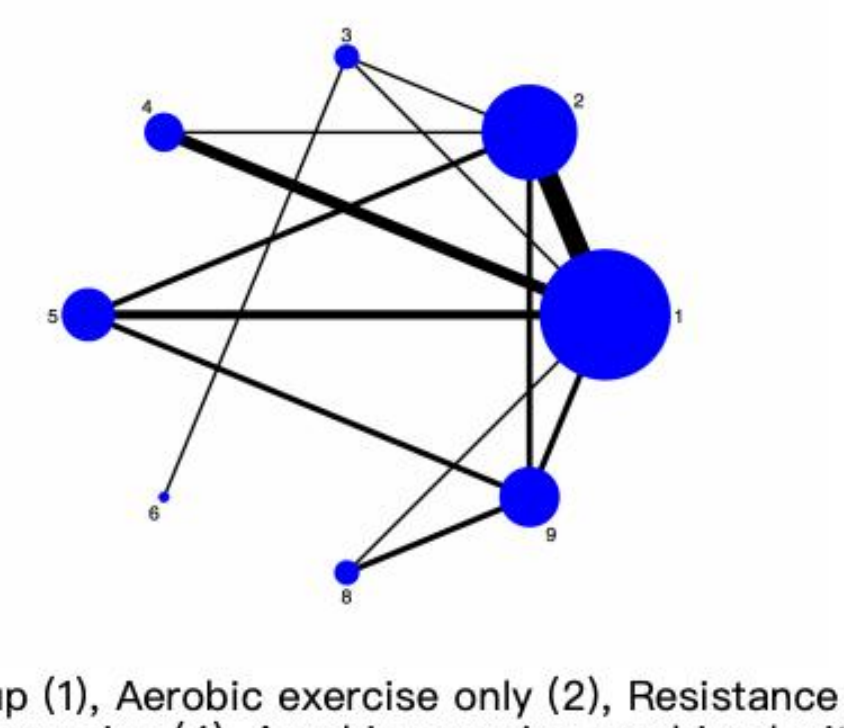

Note: Blank control group (1), Aerobic exercise only (2), Resistance exercise only(3), Aerobic plus resistance exercise (4), Aerobic exercise combined with other effective treatments (5), Resistance exercise combined with other effective treatments (6), Walking and jogging group (8), Pharmacological or psychological group(9).

F 6 Interventions in different ways for HOMA-IR network meta

Secondary Outcome

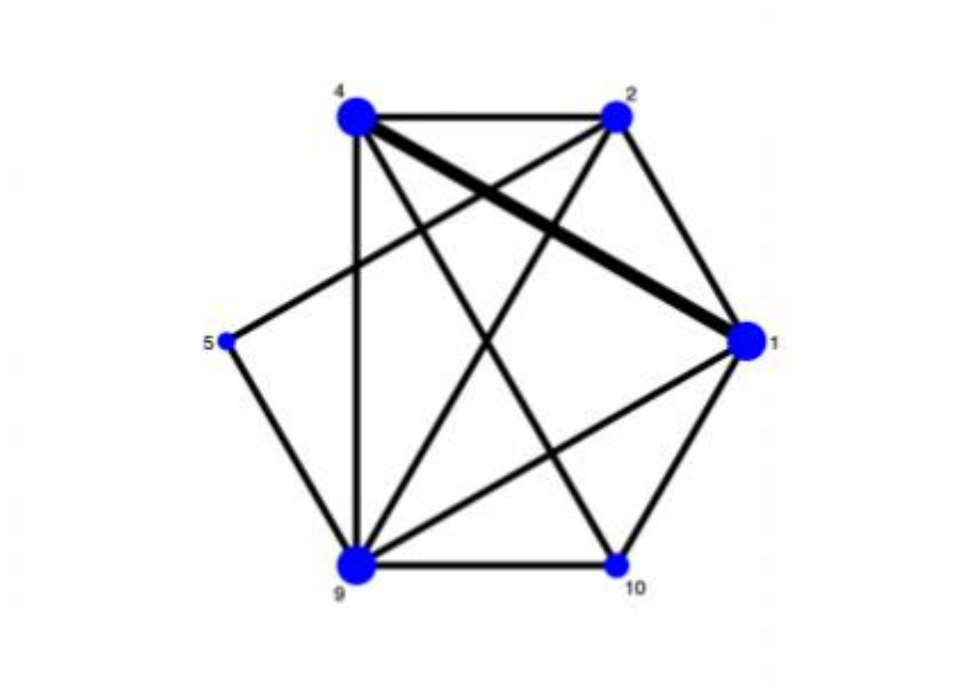

Note: Blank control group (1), Aerobic exercise only (2), Aerobic plus resistance exercise (4), Aerobic exercise combined with other effective treatments (5), Pharmacological or psychological group(9), Aerobic exercise plus resistance exercise combined with other effective treatments(10).

F 7 Interventions in different ways for IL-6 network meta

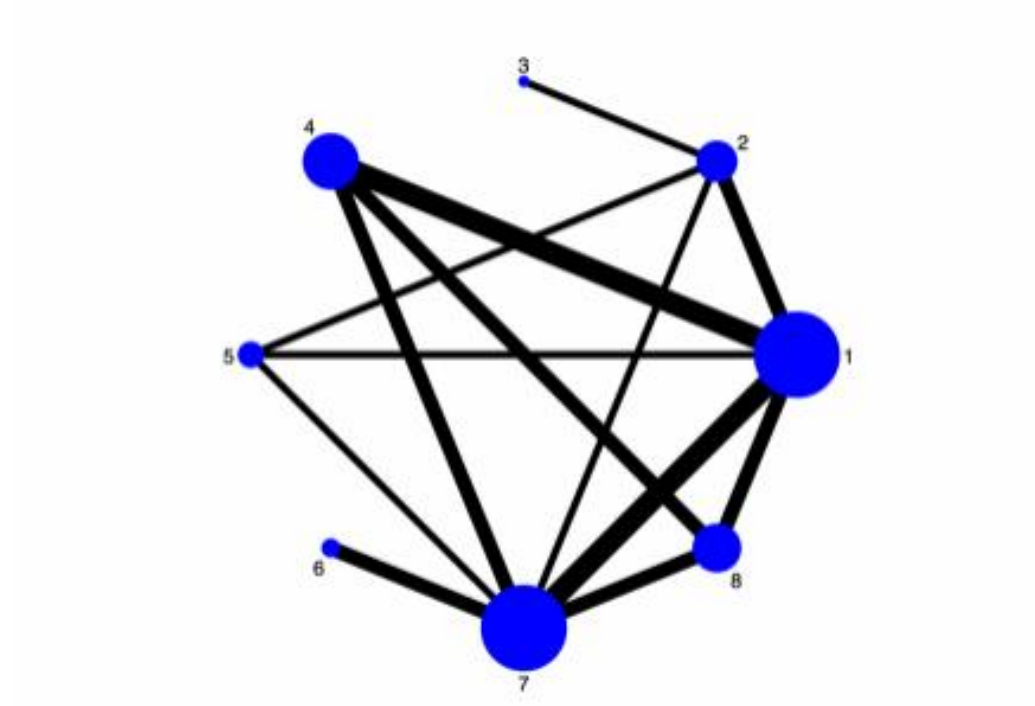

Note: Blank control group (1), Aerobic exercise only (2), Resistance exercise only(3), Aerobic plus resistance exercise (4), Aerobic exercise combined with other effective treatments (5), Resistance exercise combined with other effective treatments (6), Pharmacological or psychological group(9), Aerobic exercise plus resistance exercise combined with other effective treatments(10).

F8 Interventions in different ways for Total intake network meta

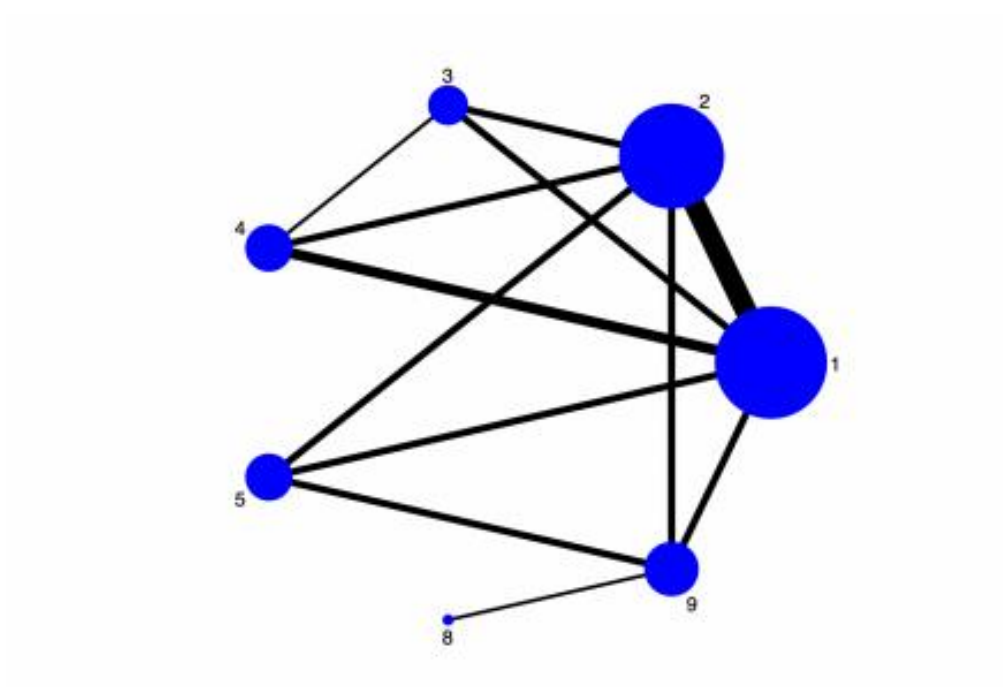

Note: Blank control group (1), Aerobic exercise only (2), Resistance exercise only(3), Aerobic plus resistance exercise (4), Aerobic exercise combined with other effective treatments (5), Walking and jogging group (8), Pharmacological or psychological group(9).

F 9 Interventions in different ways for Insulin network meta

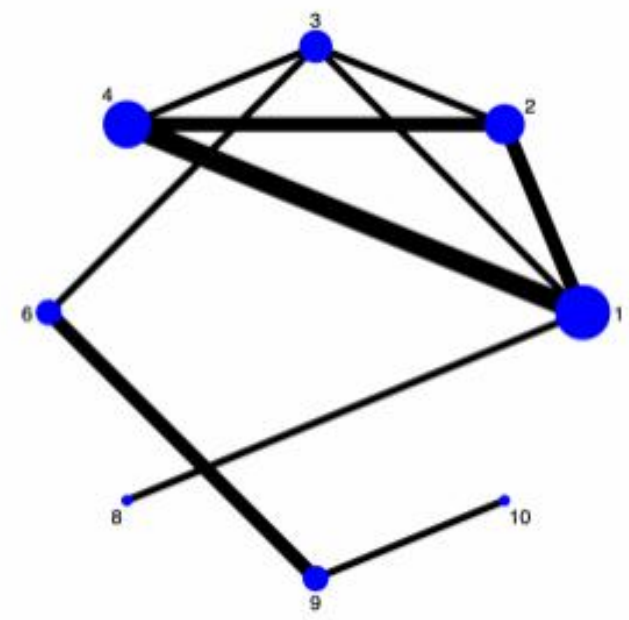

Note: Blank control group (1), Aerobic exercise only (2), Resistance exercise only(3), Aerobic plus resistance exercise (4), Resistance exercise combined with other effective treatments (6), Walking and jogging group (8), Pharmacological or psychological group(9), Aerobic exercise plus resistance exercise combined with other effective treatments(10).

F 10 Interventions in different ways for Lean body mass network meta

Appendix 2

Primary Outcome

BMI

AZO									
-0.35 (-3.56,2.86)	MBO								
-1.85 (-3.11,-0.59)	-1.50 (-4.45,1.45)	AER							
-1.82 (-3.78,0.15)	-1.47 (-4.93,1.99)	0.03 (-1.77,1.83)	AZOT						
-1.89 (-3.70,-0.08)	-1.54 (-4.82,1.74)	-0.04 (-1.47,1.38)	-0.07 (-2.24,2.09)	RES					
-2.30 (-6.50,1.89)	-1.95 (-7.07,3.16)	-0.45 (-4.63,3.73)	-0.49 (-4.89,3.91)	-0.41 (-4.78,3.95)	ROT				
-2.24 (-3.80,-0.69)	-1.89 (-5.12,1.33)	-0.39 (-1.69,0.90)	-0.43 (-2.27,1.41)	-0.35 (-2.08,1.37)	0.06 (-4.17,4.29)	AZO			
-2.60 (-3.84,-1.37)	-2.25 (-5.43,0.92)	-0.75 (-1.92,0.41)	-0.79 (-2.59,1.02)	-0.71 (-2.44,1.01)	-0.30 (-4.31,3.71)	-0.36 (-1.70,0.98)	PSY		
-3.24 (-5.70,-0.79)	-2.89 (-6.65,0.87)	-1.39 (-3.72,0.93)	-1.43 (-4.14,1.29)	-1.35 (-3.98,1.28)	-0.94 (-5.59,3.71)	-1.00 (-3.46,1.47)	-0.64 (-2.99,1.71)	WJO	
-3.67 (-4.87,-2.47)	-3.32 (-6.39,-0.25)	-1.82 (-2.68,-0.96)	-1.85 (-3.50,-0.20)	-1.78 (-3.27,-0.29)	-1.37 (-5.52,2.79)	-1.43 (-2.63,-0.22)	-1.07 (-2.15,0.02)	-0.43 (-2.61,1.76)	CON

F 1 Interventions in different ways for BMI League table

Total cholesterol

AOT									
-0.54 (-1.82,0.74)	ROT								
-0.65 (-1.52,0.23)	-0.10 (-1.28,1.07)	AZOT							
-0.68 (-1.51,0.15)	-0.14 (-1.33,1.05)	-0.03 (-0.69,0.62)	AZO						
-0.66 (-1.75,0.42)	-0.12 (-1.54,1.30)	-0.02 (-1.06,1.03)	0.02 (-0.96,1.00)	RES					
-0.94 (-1.68,-0.20)	-0.40 (-1.57,0.78)	-0.29 (-0.97,0.38)	-0.26 (-0.83,0.31)	-0.28 (-1.07,0.52)	AER				
-1.01 (-2.15,0.13)	-0.47 (-1.93,0.99)	-0.36 (-1.47,0.74)	-0.33 (-1.37,0.71)	-0.35 (-1.53,0.83)	-0.07 (-0.94,0.80)	MBO			
-1.13 (-1.98,-0.29)	-0.59 (-1.76,0.58)	-0.49 (-1.20,0.23)	-0.45 (-1.13,0.23)	-0.47 (-1.49,0.55)	-0.19 (-0.83,0.44)	-0.12 (-1.20,0.95)	WJO		
-1.14 (-1.89,-0.40)	-0.60 (-1.64,0.44)	-0.50 (-1.04,0.05)	-0.46 (-1.03,0.11)	-0.48 (-1.44,0.48)	-0.20 (-0.74,0.34)	-0.13 (-1.16,0.89)	-0.01 (-0.54,0.52)	PSY	
-1.24 (-1.95,-0.53)	-0.70 (-1.83,0.44)	-0.59 (-1.19,0.01)	-0.56 (-1.05,-0.06)	-0.58 (-1.46,0.31)	-0.30 (-0.69,0.09)	-0.23 (-1.18,0.73)	-0.10 (-0.63,0.42)	-0.10 (-0.55,0.36)	CON

F 2 Interventions in different ways for Total cholesterol League table

Triglycerides

AZOT									
-0.15 (-0.49,0.19)	AZO								
-0.28 (-0.74,0.18)	-0.13 (-0.56,0.30)	MBO							
-0.31 (-0.68,0.06)	-0.16 (-0.51,0.18)	-0.03 (-0.47,0.40)	WJO						
-0.36 (-0.89,0.17)	-0.21 (-0.73,0.31)	-0.08 (-0.67,0.51)	-0.05 (-0.55,0.46)	ROT					
-0.41 (-0.79,-0.03)	-0.26 (-0.62,0.09)	-0.13 (-0.56,0.29)	-0.10 (-0.45,0.25)	-0.05 (-0.58,0.47)	AOT				
-0.48 (-0.81,-0.15)	-0.33 (-0.62,-0.04)	-0.20 (-0.52,0.12)	-0.17 (-0.46,0.13)	-0.12 (-0.62,0.38)	-0.07 (-0.34,0.21)	AER			
-0.56 (-0.85,-0.27)	-0.41 (-0.69,-0.13)	-0.28 (-0.67,0.11)	-0.25 (-0.49,-0.00)	-0.20 (-0.64,0.24)	-0.15 (-0.43,0.13)	-0.08 (-0.30,0.14)	PSY		
-0.65 (-1.10,-0.20)	-0.50 (-0.92,-0.08)	-0.37 (-0.82,0.08)	-0.34 (-0.77,0.09)	-0.29 (-0.87,0.30)	-0.24 (-0.65,0.18)	-0.17 (-0.48,0.14)	-0.09 (-0.47,0.29)	RES	
-0.66 (-0.96,-0.36)	-0.51 (-0.76,-0.26)	-0.38 (-0.74,-0.02)	-0.34 (-0.61,-0.08)	-0.30 (-0.78,0.18)	-0.25 (-0.51,0.02)	-0.18 (-0.35,-0.01)	-0.10 (-0.29,0.09)	-0.01 (-0.36,0.34)	CON

F 3 Interventions in different ways for Triglycerides League table

LDL

AOT							
-0.09 (-0.70,0.53)	PSY						
-0.17 (-0.83,0.48)	-0.09 (-0.57,0.40)	AZO					
-0.23 (-0.69,0.24)	-0.14 (-0.54,0.26)	-0.05 (-0.51,0.41)	WJO				
-0.30 (-0.91,0.31)	-0.21 (-0.73,0.30)	-0.13 (-0.65,0.40)	-0.07 (-0.48,0.33)	ROT			
-0.53 (-1.14,0.08)	-0.44 (-0.90,0.01)	-0.36 (-0.77,0.06)	-0.30 (-0.70,0.10)	-0.23 (-0.63,0.17)	CON		
-0.64 (-1.36,0.08)	-0.56 (-1.14,0.03)	-0.47 (-1.03,0.09)	-0.42 (-0.96,0.13)	-0.34 (-0.89,0.20)	-0.11 (-0.48,0.26)	AER	

F 4 Interventions in different ways for LDL-C League table

HDL

ROT									
-0.10 (-0.31,0.11)	PSY								
-0.20 (-0.43,0.03)	-0.10 (-0.20,-0.00)	RES							
-0.29 (-0.51,-0.07)	-0.19 (-0.26,-0.12)	-0.09 (-0.20,0.02)	WJO						
-0.33 (-0.56,-0.11)	-0.23 (-0.31,-0.15)	-0.13 (-0.20,-0.06)	-0.04 (-0.13,0.05)	CON					
-0.34 (-0.56,-0.12)	-0.24 (-0.31,-0.17)	-0.14 (-0.26,-0.02)	-0.05 (-0.14,0.05)	-0.01 (-0.11,0.10)	AZOT				
-0.37 (-0.60,-0.15)	-0.27 (-0.36,-0.19)	-0.17 (-0.22,-0.12)	-0.08 (-0.18,0.02)	-0.04 (-0.09,0.01)	-0.03 (-0.14,0.08)	AER			
-0.44 (-0.71,-0.18)	-0.34 (-0.51,-0.18)	-0.24 (-0.40,-0.08)	-0.15 (-0.32,0.02)	-0.11 (-0.26,0.04)	-0.10 (-0.28,0.08)	-0.07 (-0.22,0.08)	AZO		
-0.46 (-0.76,-0.17)	-0.36 (-0.57,-0.15)	-0.26 (-0.46,-0.06)	-0.17 (-0.38,0.04)	-0.13 (-0.33,0.07)	-0.12 (-0.34,0.10)	-0.09 (-0.28,0.10)	-0.02 (-0.26,0.22)	MBO	
-0.71 (-0.93,-0.48)	-0.61 (-0.70,-0.52)	-0.51 (-0.62,-0.40)	-0.42 (-0.52,-0.31)	-0.38 (-0.47,-0.28)	-0.37 (-0.48,-0.26)	-0.34 (-0.43,-0.24)	-0.27 (-0.44,-0.09)	-0.25 (-0.46,-0.03)	AOT

F 5 Interventions in different ways for HDL-C League table

HOMA-IR

AOT							
-0.75 (-2.35,0.85)	WJO						
-0.85 (-1.99,0.29)	-0.10 (-1.32,1.12)	PSY					
-0.89 (-2.13,0.36)	-0.13 (-1.86,1.59)	-0.03 (-1.38,1.31)	AZO				
-1.10 (-3.39,1.18)	-0.35 (-2.93,2.23)	-0.25 (-2.59,2.09)	-0.22 (-2.51,2.07)	ROT			
-1.12 (-2.09,-0.16)	-0.37 (-1.91,1.17)	-0.27 (-1.35,0.80)	-0.24 (-1.24,0.76)	-0.02 (-2.15,2.11)	AER		
-1.42 (-3.06,0.21)	-0.67 (-2.70,1.35)	-0.57 (-2.28,1.14)	-0.54 (-2.18,1.10)	-0.32 (-1.92,1.28)	-0.30 (-1.71,1.11)	RES	
-1.91 (-2.82,-1.00)	-1.15 (-2.65,0.34)	-1.06 (-2.10,-0.01)	-1.02 (-1.90,-0.14)	-0.80 (-2.93,1.33)	-0.78 (-1.40,-0.16)	-0.48 (-1.89,0.93)	CON

F 6 Interventions in different ways for HOMA-IR League table

Secondary Outcome Measures

IL-6

AOT					
-2.31 (-4.15,-0.48)	AZOT				
-2.82 (-4.26,-1.38)	-0.50 (-1.80,0.79)	AZO			
-3.04 (-4.47,-1.61)	-0.73 (-2.18,0.73)	-0.22 (-1.01,0.57)	AER		
-4.13 (-5.43,-2.82)	-1.81 (-3.20,-0.43)	-1.31 (-2.16,-0.46)	-1.09 (-2.09,-0.08)	PSY	
-4.36 (-5.84,-2.88)	-2.05 (-3.37,-0.72)	-1.54 (-2.25,-0.83)	-1.32 (-2.22,-0.42)	-0.23 (-1.13,0.67)	CON

F 7 Interventions in different ways for IL-6 League table

Insulin

WJO						
-1.88 (-5.78,2.02)	AOT					
-2.05 (-6.60,2.51)	-0.16 (-3.41,3.08)	AZO				
-2.35 (-5.57,0.87)	-0.47 (-2.67,1.74)	-0.30 (-3.53,2.92)	PSY			
-3.22 (-7.05,0.62)	-1.33 (-3.47,0.80)	-1.17 (-3.87,1.53)	-0.87 (-2.95,1.22)	AER		
-3.63 (-7.49,0.22)	-1.75 (-3.91,0.41)	-1.58 (-4.12,0.95)	-1.28 (-3.41,0.84)	-0.41 (-1.86,1.03)	CON	
-4.74 (-9.22,-0.25)	-2.85 (-5.96,0.25)	-2.69 (-5.85,0.47)	-2.39 (-5.51,0.74)	-1.52 (-3.93,0.89)	-1.11 (-3.45,1.24)	RES

F 8 Interventions in different ways for Insulin League table

Lean body mass

PSY							
-3.98 (-9.53,1.58)	ROT						
-4.66 (-11.23,1.92)	-0.68 (-4.19,2.83)	AZO					
-4.74 (-11.30,1.81)	-0.77 (-4.24,2.71)	-0.09 (-1.26,1.09)	CON				
-4.70 (-8.49,-0.91)	-0.72 (-7.45,6.00)	-0.04 (-7.64,7.55)	0.04 (-7.53,7.62)	AZOT			
-5.14 (-12.71,2.43)	-1.17 (-6.31,3.97)	-0.49 (-4.45,3.48)	-0.40 (-4.19,3.39)	-0.44 (-8.91,8.03)	WJO		
-5.44 (-12.05,1.17)	-1.46 (-5.05,2.12)	-0.78 (-2.16,0.59)	-0.70 (-2.03,0.63)	-0.74 (-8.36,6.88)	-0.30 (-4.31,3.72)	AER	
-5.45 (-11.88,0.99)	-1.47 (-4.71,1.77)	-0.79 (-2.16,0.58)	-0.70 (-1.98,0.57)	-0.75 (-8.21,6.72)	-0.30 (-4.30,3.69)	-0.01 (-1.55,1.53)	RES

F 9 Interventions in different ways for Lean body mass League table

Note: Blank control group (CON), Aerobic exercise only (AER), Resistance exercise only(RES), Aerobic

plus resistance exercise (AZO), Aerobic exercise combined with other effective treatments (AOT), Resistance exercise combined with other effective treatments (ROT), Mind-body exercise group (MBO), Walking and jogging group (WJO), Pharmacological or psychological group(PSY), Aerobic exercise plus resistance exercise combined with other effective treatments(AZTO).

Appendix 3

Primary Outcome

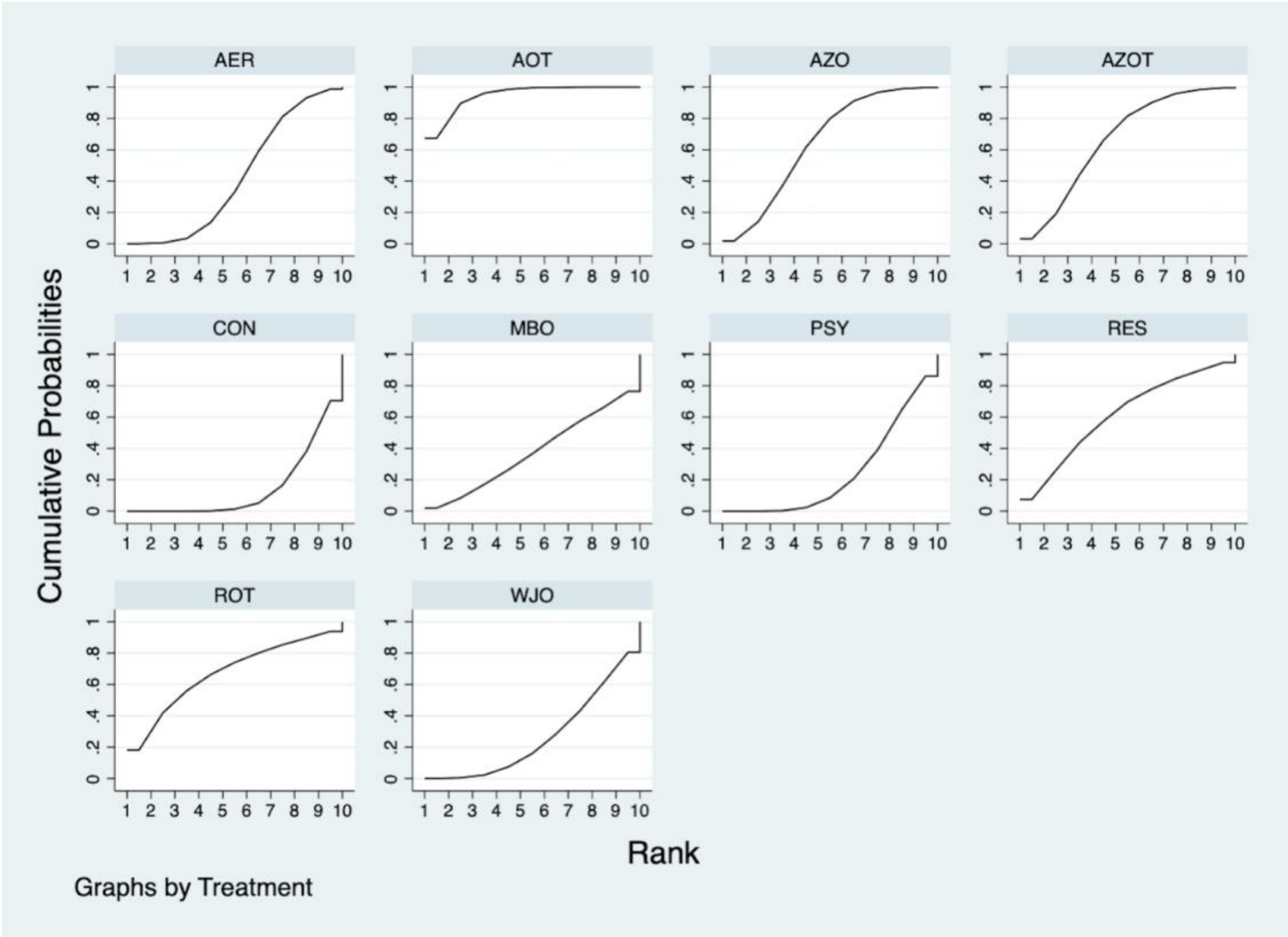

F 1 Interventions in different ways for BMI SUCRA Ranking chart

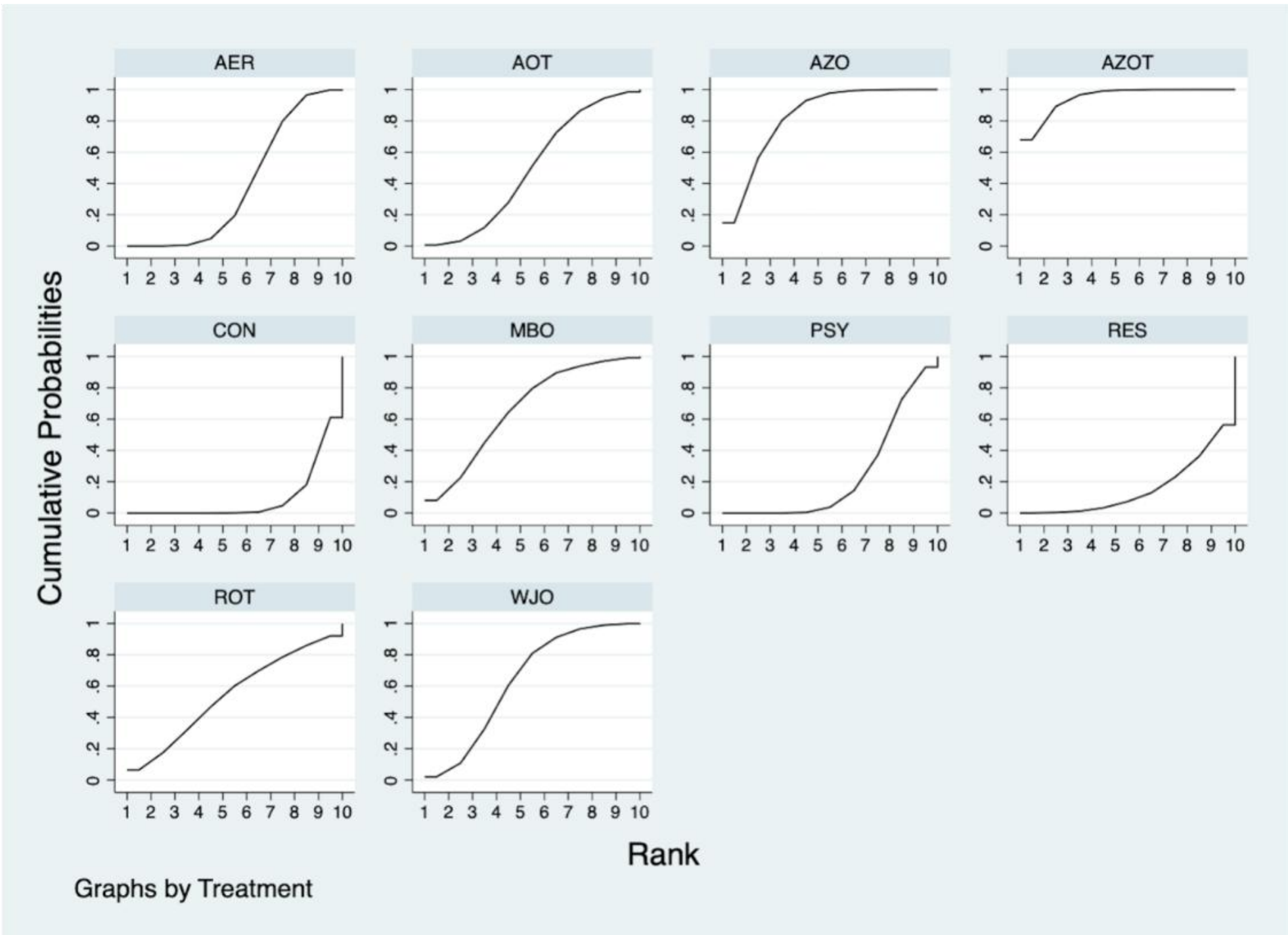

F 2 Interventions in different ways for Total cholesterol SUCRA Ranking chart

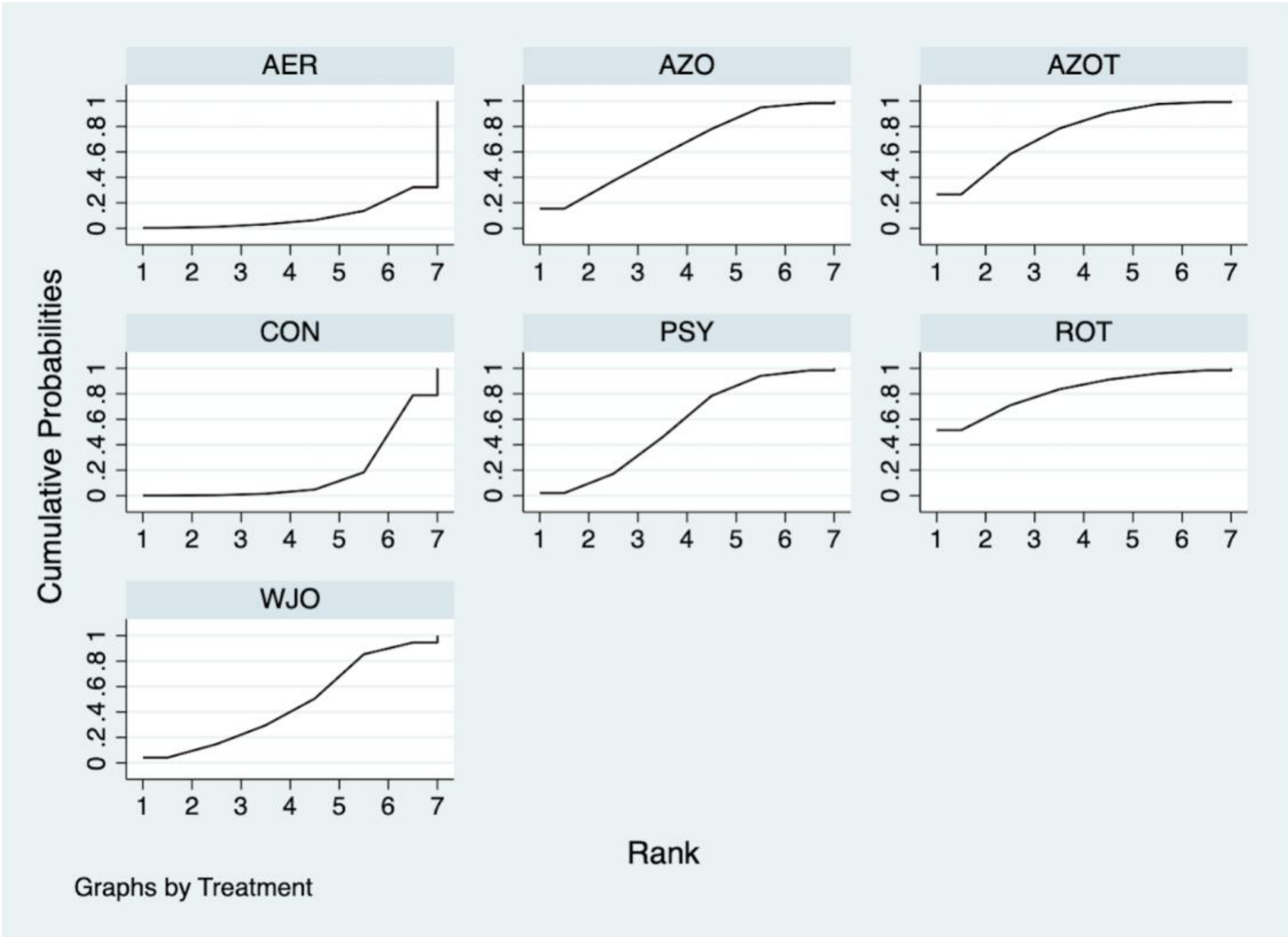

F 3 Interventions in different ways for Triglycerides SUCRA Ranking chart

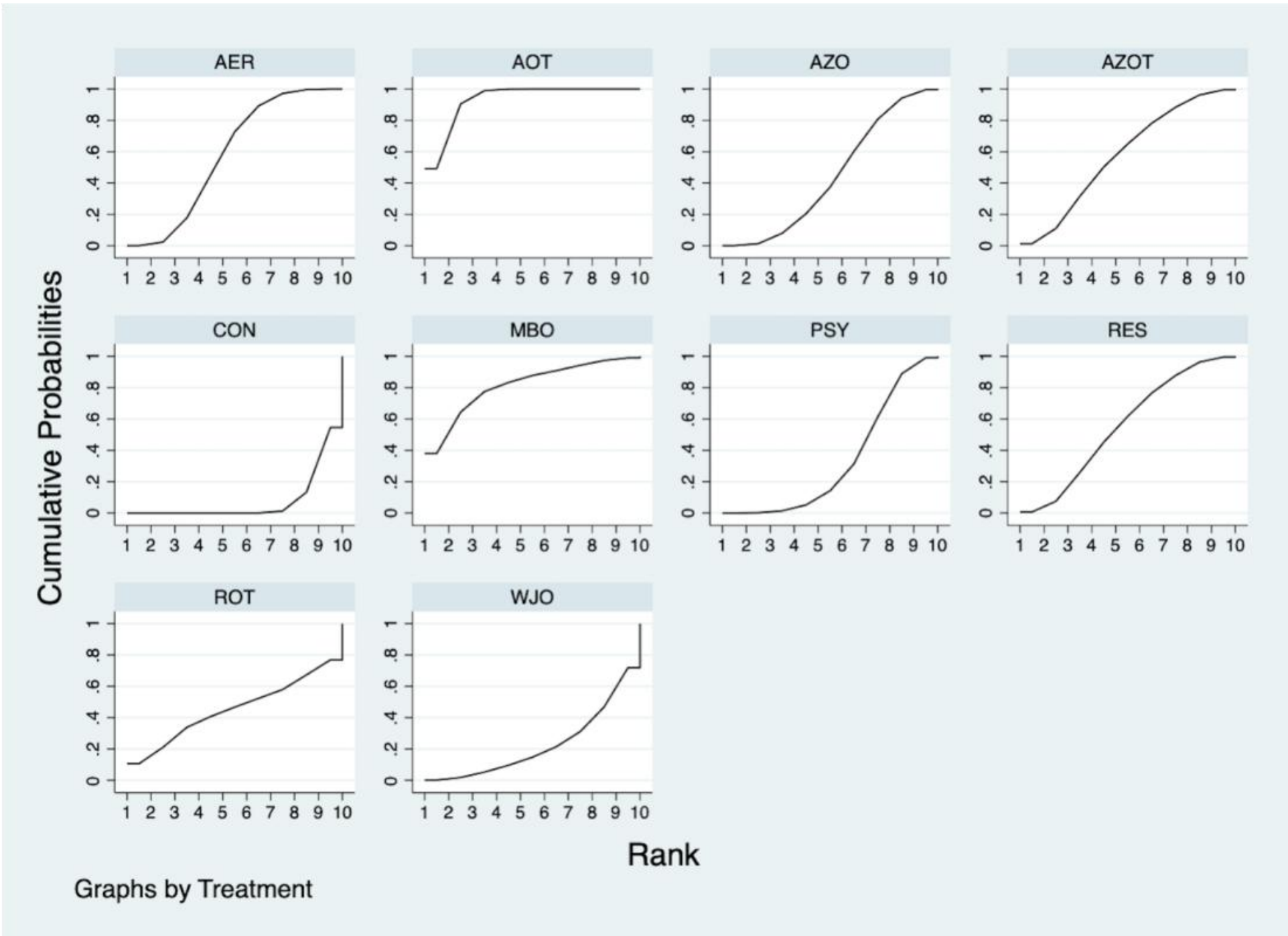

F 4 Interventions in different ways for LDL-C SUCRA Ranking chart

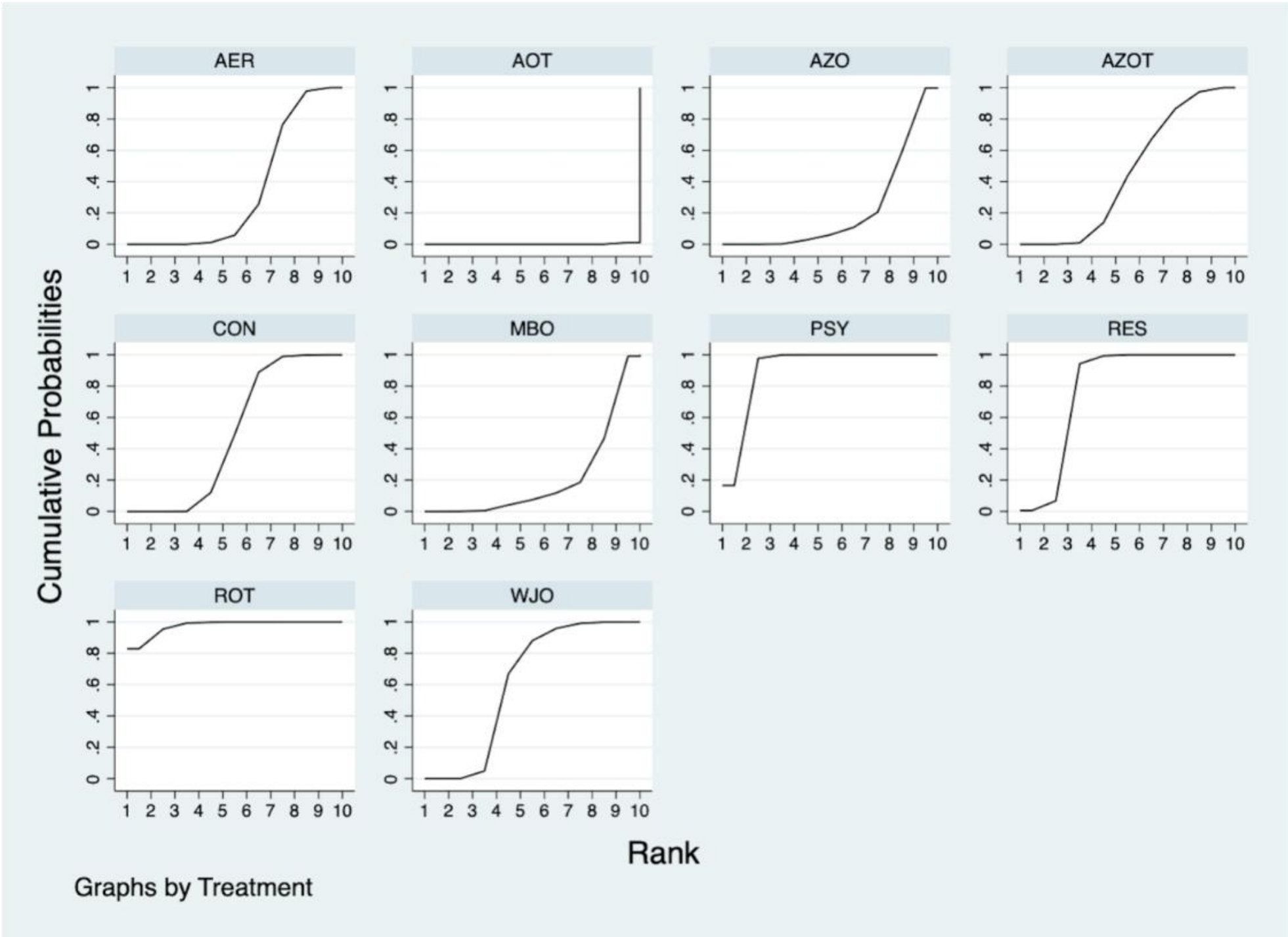

F 5 Interventions in different ways for HDL-C SUCRA Ranking chart

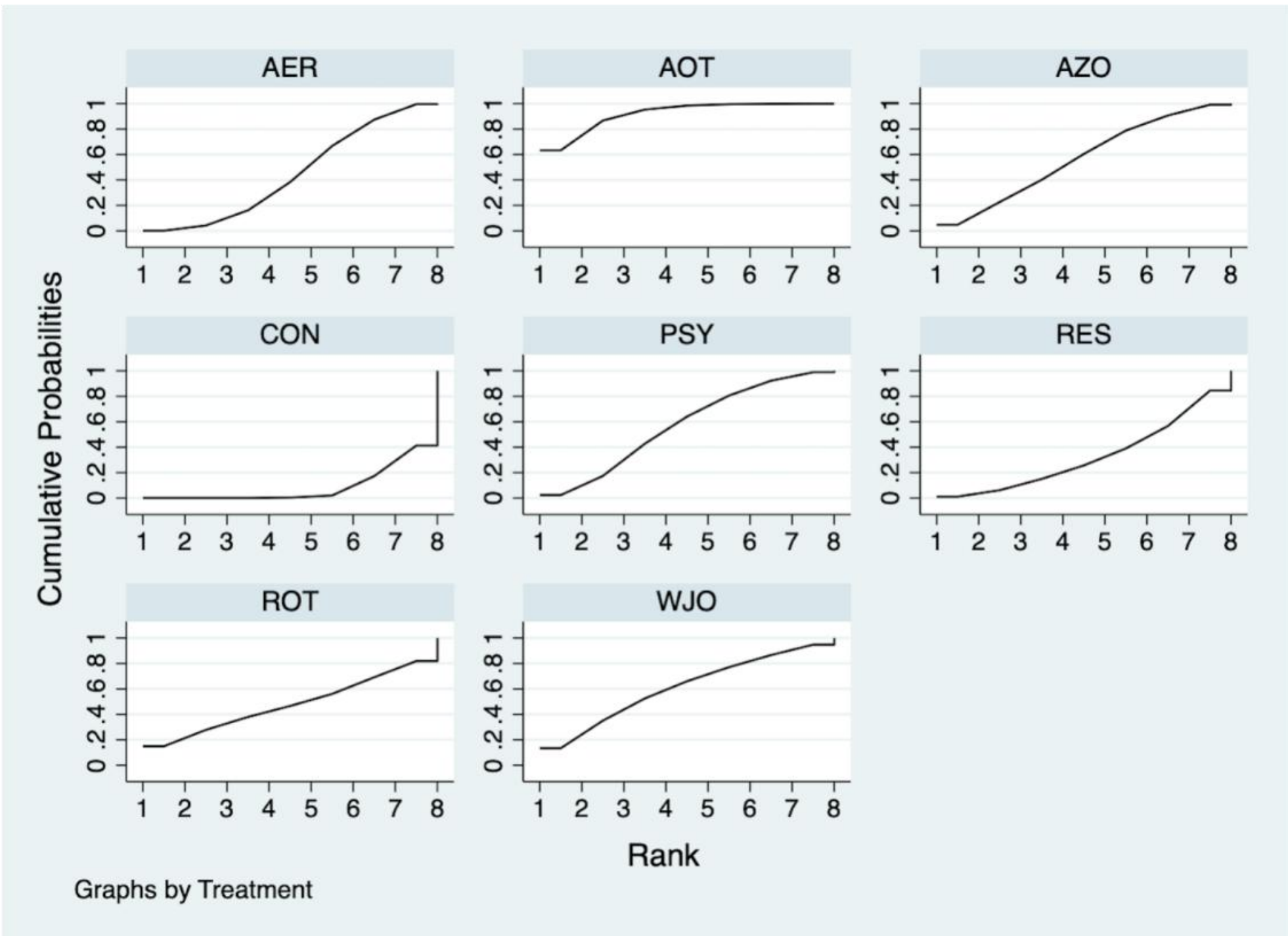

F 6 Interventions in different ways for HOMA-IR SUCRA Ranking chart

Secondary Outcome

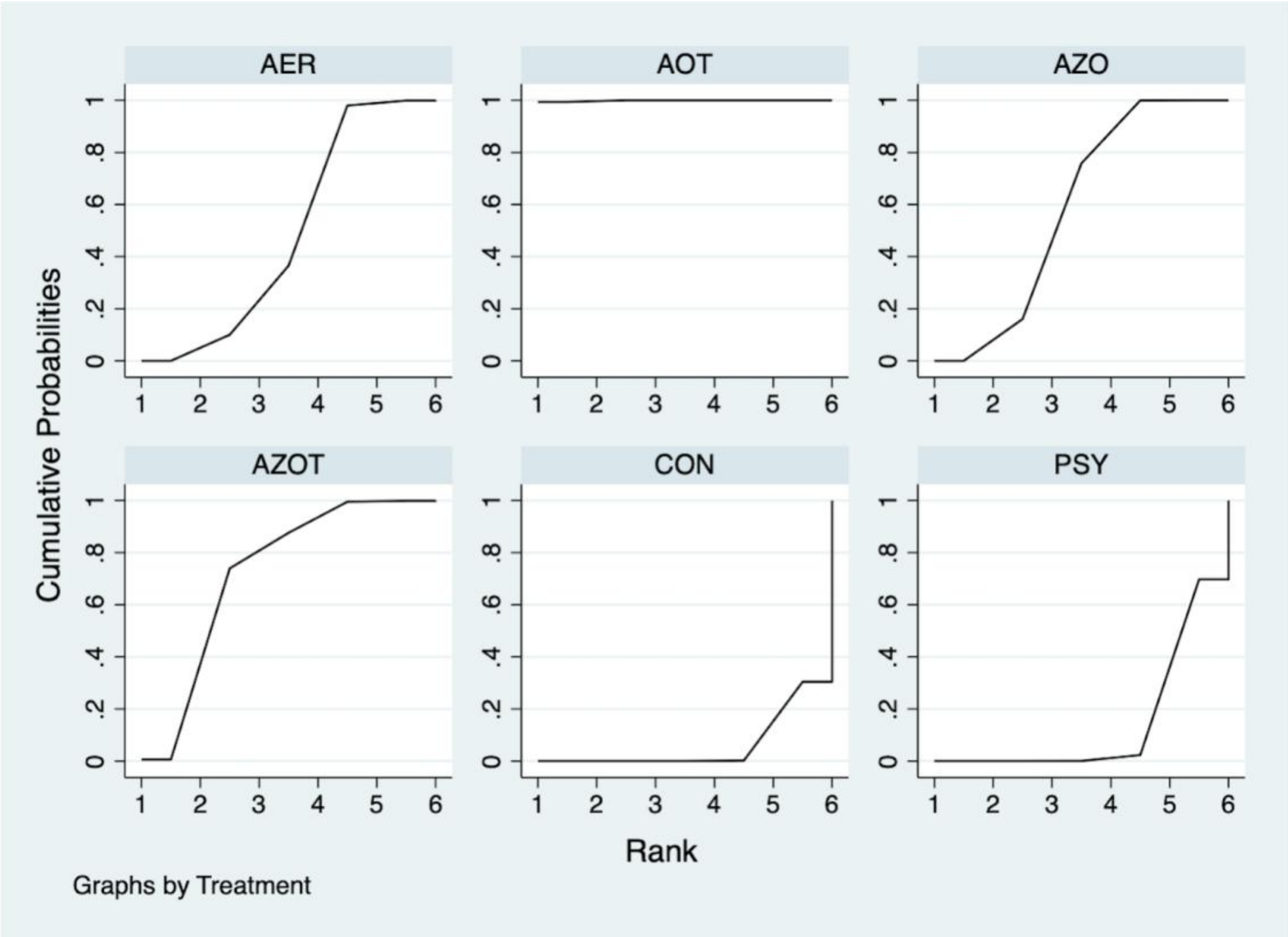

F 7 Interventions in different ways for IL-6 SUCRA Ranking chart

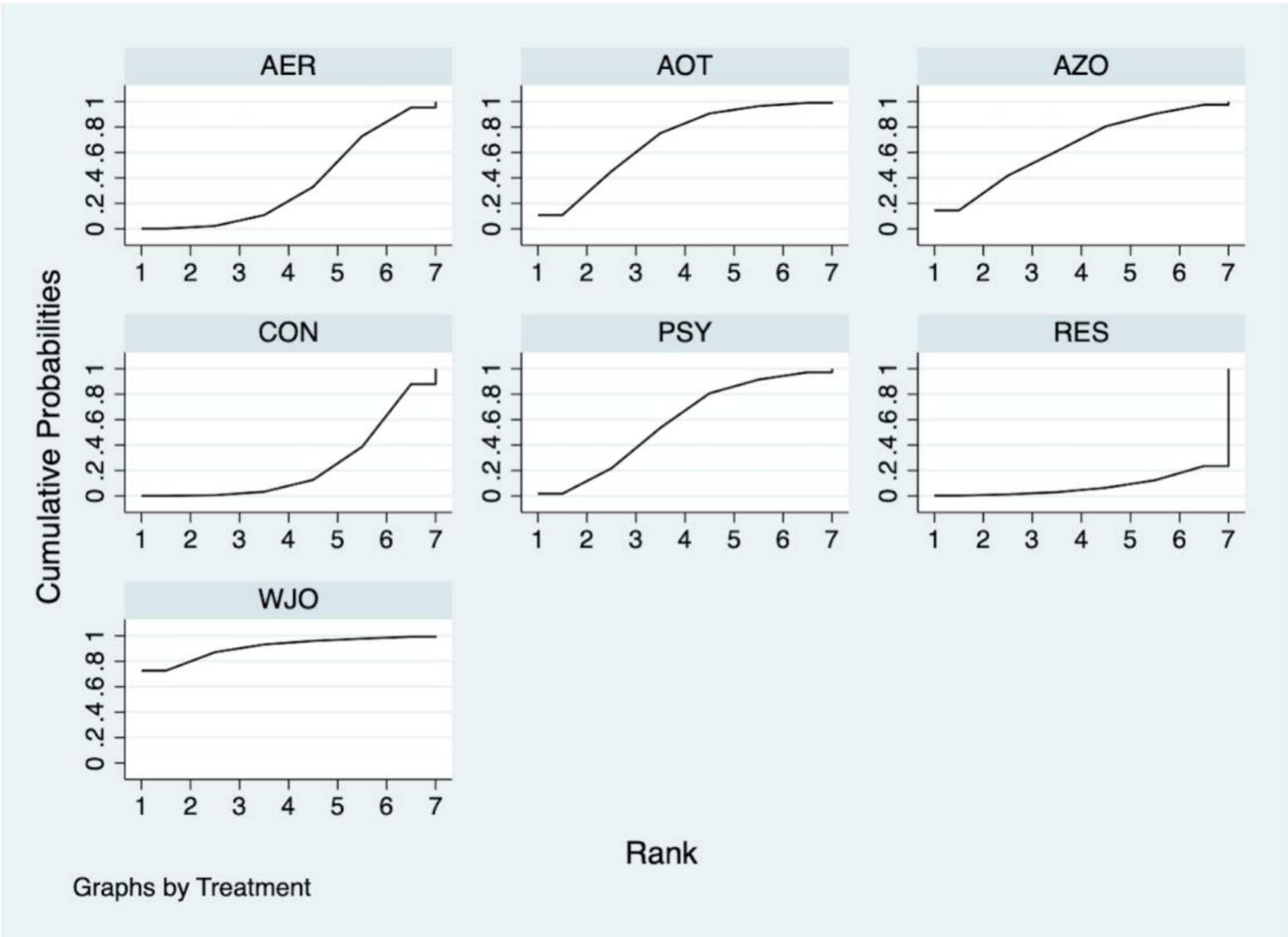

F 8 Interventions in different ways for Insulin SUCRA Ranking chart

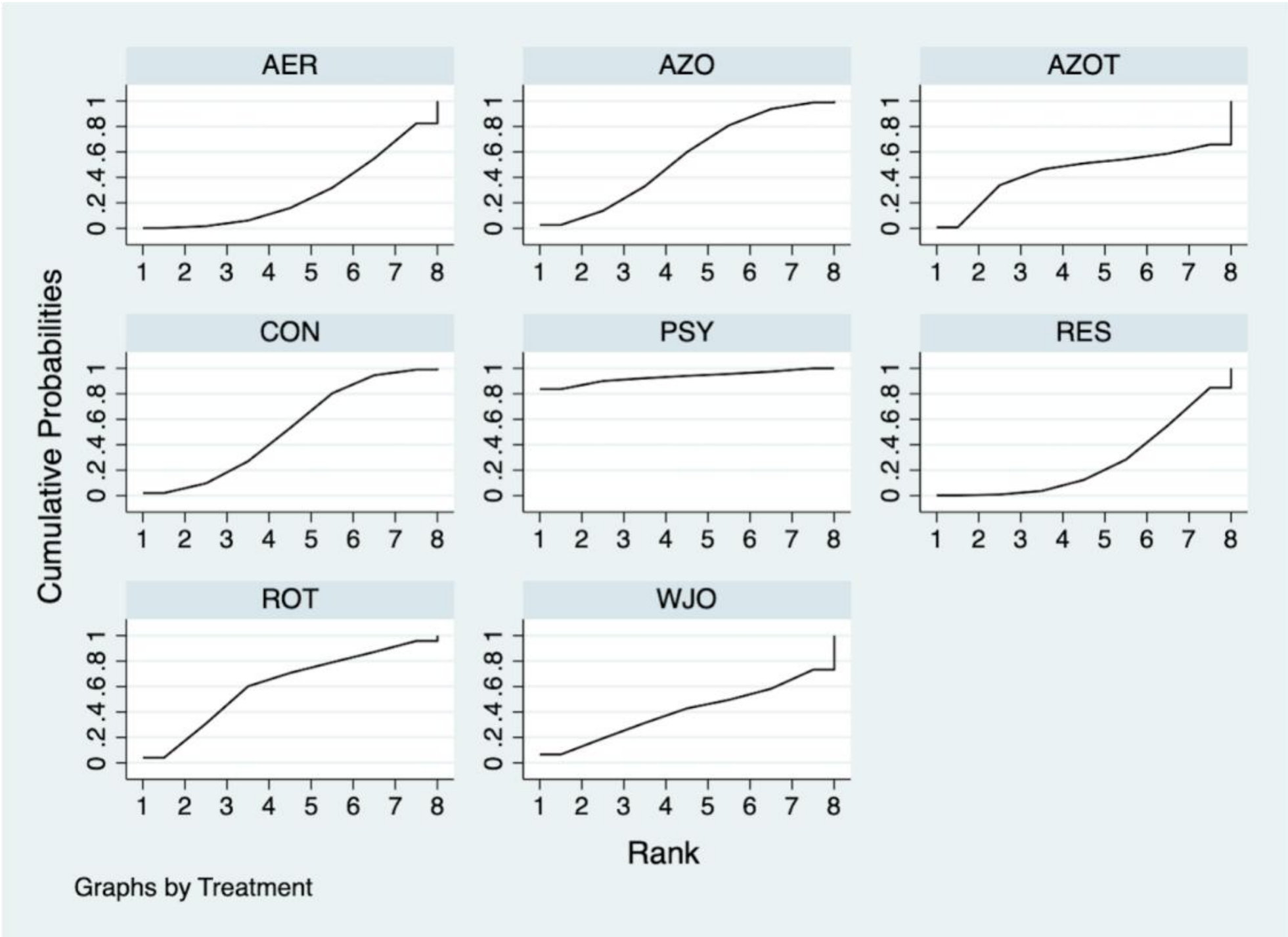

F 9 Interventions in different ways for Lean body mass SUCRA Ranking chart

Note: Blank control group (CON), Aerobic exercise only (AER), Resistance exercise only(RES), Aerobic plus resistance exercise (AZO), Aerobic exercise combined with other effective treatments (AOT), Resistance exercise combined with other effective treatments (ROT), Mind-body exercise group (MBO), Walking and jogging group (WJO), Pharmacological or psychological group(PSY), Aerobic exercise plus resistance exercise combined with other effective treatments(AZTO).

Appendix 4

Primary Outcome

exercise for Type 2 diabetes and obesity

Patient or population: patients with Type 2 diabetes and obesity
Settings:
Intervention: exercise

Outcomes	Illustrative comparative risks* (95% CI)		Relative effect (95% CI)	No of Participants (studies)	Quality of the evidence (GRADE)	Comments
	Assumed risk	Corresponding risk				
	Control	Exercise				
BMI		The mean bmi in the intervention groups was **1.78 lower** (2.7 to 0.85 lower)		937 (25 studies)	⊕⊕⊖⊖ **low**[1]	

*The basis for the **assumed risk** (e.g. the median control group risk across studies) is provided in footnotes. The **corresponding risk** (and its 95% confidence interval) is based on the assumed risk in the comparison group and the **relative effect** of the intervention (and its 95% CI).

CI: Confidence interval;

GRADE Working Group grades of evidence
High quality: Further research is very unlikely to change our confidence in the estimate of effect.
Moderate quality: Further research is likely to have an important impact on our confidence in the estimate of effect and may change the estimate.
Low quality: Further research is very likely to have an important impact on our confidence in the estimate of effect and is likely to change the estimate.
Very low quality: We are very uncertain about the estimate.

[1] High heterogeneity

F 1 GRADE Evidence Quality Assessment for BMI

TC for Type 2 diabetes and obesity

Patient or population: patients with Type 2 diabetes and obesity
Settings:
Intervention: TC

Outcomes	Illustrative comparative risks* (95% CI)		Relative effect (95% CI)	No of Participants (studies)	Quality of the evidence (GRADE)	Comments
	Assumed risk	Corresponding risk				
	Control	TC				
TC		The mean tc in the intervention groups was **0.36 lower** (0.59 to 0.14 lower)		479 (15 studies)	⊕⊕⊖⊖ **low**[1]	

*The basis for the **assumed risk** (e.g. the median control group risk across studies) is provided in footnotes. The **corresponding risk** (and its 95% confidence interval) is based on the assumed risk in the comparison group and the **relative effect** of the intervention (and its 95% CI).

CI: Confidence interval;

GRADE Working Group grades of evidence
High quality: Further research is very unlikely to change our confidence in the estimate of effect.
Moderate quality: Further research is likely to have an important impact on our confidence in the estimate of effect and may change the estimate.
Low quality: Further research is very likely to have an important impact on our confidence in the estimate of effect and is likely to change the estimate.
Very low quality: We are very uncertain about the estimate.

[1] High heterogeneity

F 2 GRADE Evidence Quality Assessment for TC

TG for Type 2 diabetes and obesity

Patient or population: patients with Type 2 diabetes and obesity
Settings:
Intervention: TG

Outcomes	Illustrative comparative risks* (95% CI)		Relative effect (95% CI)	No of Participants (studies)	Quality of the evidence (GRADE)	Comments
	Assumed risk	Corresponding risk				
	Control	TG				
TG		The mean tg in the intervention groups was **0.28 lower** (0.38 to 0.19 lower)		509 (16 studies)	⊕⊕⊕⊖ **moderate**[1]	

*The basis for the **assumed risk** (e.g. the median control group risk across studies) is provided in footnotes. The **corresponding risk** (and its 95% confidence interval) is based on the assumed risk in the comparison group and the **relative effect** of the intervention (and its 95% CI).

CI: Confidence interval;

GRADE Working Group grades of evidence
High quality: Further research is very unlikely to change our confidence in the estimate of effect.
Moderate quality: Further research is likely to have an important impact on our confidence in the estimate of effect and may change the estimate.
Low quality: Further research is very likely to have an important impact on our confidence in the estimate of effect and is likely to change the estimate.
Very low quality: We are very uncertain about the estimate.

[1] High heterogeneity

F 3 GRADE Evidence Quality Assessment for TG

LDL for Type 2 diabetes and obesity

Patient or population: patients with Type 2 diabetes and obesity
Settings:
Intervention: LDL

Outcomes	Illustrative comparative risks* (95% CI)		Relative effect (95% CI)	No of Participants (studies)	Quality of the evidence (GRADE)	Comments
	Assumed risk	Corresponding risk				
	Control	LDL				
LDL		The mean ldl in the intervention groups was **0.19 lower** (0.35 to 0.04 lower)		334 (12 studies)	⊕⊖⊖⊖ **very low**[1,2]	

*The basis for the **assumed risk** (e.g. the median control group risk across studies) is provided in footnotes. The **corresponding risk** (and its 95% confidence interval) is based on the assumed risk in the comparison group and the **relative effect** of the intervention (and its 95% CI).

CI: Confidence interval;

GRADE Working Group grades of evidence
High quality: Further research is very unlikely to change our confidence in the estimate of effect.
Moderate quality: Further research is likely to have an important impact on our confidence in the estimate of effect and may change the estimate.
Low quality: Further research is very likely to have an important impact on our confidence in the estimate of effect and is likely to change the estimate.
Very low quality: We are very uncertain about the estimate.

[1] The sample size is relatively small.
[2] High heterogeneity

F 4 GRADE Evidence Quality Assessment for LDL-C

HDL for Type 2 diabetes and obesity

Patient or population: patients with Type 2 diabetes and obesity
Settings:
Intervention: HDL

Outcomes	Illustrative comparative risks* (95% CI)		Relative effect (95% CI)	No of Participants (studies)	Quality of the evidence (GRADE)	Comments
	Assumed risk	Corresponding risk				
	Control	HDL				
HDL		The mean hdl in the intervention groups was **0.12 higher** (0.05 to 0.18 higher)		452 (14 studies)	⊕⊕⊖⊖ **low**[1]	

*The basis for the **assumed risk** (e.g. the median control group risk across studies) is provided in footnotes. The **corresponding risk** (and its 95% confidence interval) is based on the assumed risk in the comparison group and the **relative effect** of the intervention (and its 95% CI).

CI: Confidence interval;

GRADE Working Group grades of evidence
High quality: Further research is very unlikely to change our confidence in the estimate of effect.
Moderate quality: Further research is likely to have an important impact on our confidence in the estimate of effect and may change the estimate.
Low quality: Further research is very likely to have an important impact on our confidence in the estimate of effect and is likely to change the estimate.
Very low quality: We are very uncertain about the estimate.

[1] High heterogeneity

F 5 GRADE Evidence Quality Assessment for HDL-C

HOMA-IR for Type 2 diabetes and obesity

Patient or population: patients with Type 2 diabetes and obesity
Settings:
Intervention: HOMA-IR

Outcomes	Illustrative comparative risks* (95% CI)		Relative effect (95% CI)	No of Participants (studies)	Quality of the evidence (GRADE)	Comments
	Assumed risk	Corresponding risk				
	Control	HOMA-IR				
HOMA-IR		The mean homa-ir in the intervention groups was **0.92 lower** (1.73 to 0.1 lower)		723 (15 studies)	⊕⊕⊖⊖ **low**[1]	

*The basis for the **assumed risk** (e.g. the median control group risk across studies) is provided in footnotes. The **corresponding risk** (and its 95% confidence interval) is based on the assumed risk in the comparison group and the **relative effect** of the intervention (and its 95% CI).

CI: Confidence interval;

GRADE Working Group grades of evidence
High quality: Further research is very unlikely to change our confidence in the estimate of effect.
Moderate quality: Further research is likely to have an important impact on our confidence in the estimate of effect and may change the estimate.
Low quality: Further research is very likely to have an important impact on our confidence in the estimate of effect and is likely to change the estimate.
Very low quality: We are very uncertain about the estimate.

[1] High heterogeneity

F 6 GRADE Evidence Quality Assessment for HOMA-IR

Secondary Outcome

IL-6 for Type 2 diabetes and obesity

Patient or population: patients with Type 2 diabetes and obesity
Settings:
Intervention: IL-6

Outcomes	Illustrative comparative risks* (95% CI)		Relative effect (95% CI)	No of Participants (studies)	Quality of the evidence (GRADE)	Comments
	Assumed risk	Corresponding risk				
	Control	IL-6				
IL-6		The mean il-6 in the intervention groups was **3.12 lower** (6.22 to 0.01 lower)		66 (3 studies)	⊕⊖⊖⊖ **very low**[1,2]	

*The basis for the **assumed risk** (e.g. the median control group risk across studies) is provided in footnotes. The **corresponding risk** (and its 95% confidence interval) is based on the assumed risk in the comparison group and the **relative effect** of the intervention (and its 95% CI).

CI: Confidence interval;

GRADE Working Group grades of evidence
High quality: Further research is very unlikely to change our confidence in the estimate of effect.
Moderate quality: Further research is likely to have an important impact on our confidence in the estimate of effect and may change the estimate.
Low quality: Further research is very likely to have an important impact on our confidence in the estimate of effect and is likely to change the estimate.
Very low quality: We are very uncertain about the estimate.

[1] The sample size is relatively small.
[2] High heterogeneity

F 7 GRADE Evidence Quality Assessment for IL-6

exercise for Type 2 diabetes and obesity

Patient or population: patients with Type 2 diabetes and obesity
Settings:
Intervention: exercise

Outcomes	Illustrative comparative risks* (95% CI)		Relative effect (95% CI)	No of Participants (studies)	Quality of the evidence (GRADE)	Comments
	Assumed risk	Corresponding risk				
	Control	Exercise				
Total intake		The mean total intake in the intervention groups was **156.52 lower** (285.76 to 27.28 lower)		261 (10 studies)	⊕⊖⊖⊖ **very low**[1,2,3]	

*The basis for the **assumed risk** (e.g. the median control group risk across studies) is provided in footnotes. The **corresponding risk** (and its 95% confidence interval) is based on the assumed risk in the comparison group and the **relative effect** of the intervention (and its 95% CI).

CI: Confidence interval;

GRADE Working Group grades of evidence
High quality: Further research is very unlikely to change our confidence in the estimate of effect.
Moderate quality: Further research is likely to have an important impact on our confidence in the estimate of effect and may change the estimate.
Low quality: Further research is very likely to have an important impact on our confidence in the estimate of effect and is likely to change the estimate.
Very low quality: We are very uncertain about the estimate.

[1] A sample size less than 30 units
[2] The 95% confidence interval includes 0
[3] High heterogeneity

F 8 GRADE Evidence Quality Assessment for Total intake

Insulin for Type 2 diabetes and obesity

Patient or population: patients with Type 2 diabetes and obesity
Settings:
Intervention: Insulin

Outcomes	Illustrative comparative risks* (95% CI)		Relative effect (95% CI)	No of Participants (studies)	Quality of the evidence (GRADE)	Comments
	Assumed risk	Corresponding risk				
	Control	Insulin				
Insulin		The mean insulin in the intervention groups was **1.16 lower** (2.14 to 0.18 lower)		207 (8 studies)	⊕⊕⊖⊖ **low**[1,2]	

*The basis for the **assumed risk** (e.g. the median control group risk across studies) is provided in footnotes. The **corresponding risk** (and its 95% confidence interval) is based on the assumed risk in the comparison group and the **relative effect** of the intervention (and its 95% CI).

CI: Confidence interval;

GRADE Working Group grades of evidence
High quality: Further research is very unlikely to change our confidence in the estimate of effect.
Moderate quality: Further research is likely to have an important impact on our confidence in the estimate of effect and may change the estimate.
Low quality: Further research is very likely to have an important impact on our confidence in the estimate of effect and is likely to change the estimate.
Very low quality: We are very uncertain about the estimate.

[1] The sample size is relatively small.
[2] High heterogeneity

F 9 GRADE Evidence Quality Assessment for Insulin

Lean body mass for Type 2 diabetes and obesity

Patient or population: patients with Type 2 diabetes and obesity
Settings:
Intervention: Lean body mass

Outcomes	Illustrative comparative risks* (95% CI)		Relative effect (95% CI)	No of Participants (studies)	Quality of the evidence (GRADE)	Comments
	Assumed risk	Corresponding risk				
	**Control**	**Lean body mass**				
**Lean body mass**		The mean lean body mass in the intervention groups was **0.24 standard deviations higher** (0.04 to 0.44 higher)		380 (8 studies)	⊕⊕⊕⊖ **moderate**[1]	SMD 0.24 (0.04 to 0.44)

*The basis for the **assumed risk** (e.g. the median control group risk across studies) is provided in footnotes. The **corresponding risk** (and its 95% confidence interval) is based on the assumed risk in the comparison group and the **relative effect** of the intervention (and its 95% CI).

CI: Confidence interval;

GRADE Working Group grades of evidence
High quality: Further research is very unlikely to change our confidence in the estimate of effect.
Moderate quality: Further research is likely to have an important impact on our confidence in the estimate of effect and may change the estimate.
Low quality: Further research is very likely to have an important impact on our confidence in the estimate of effect and is likely to change the estimate.
Very low quality: We are very uncertain about the estimate.

[1] Nearly two-thirds are of moderate bias

F 10 GRADE Evidence Quality Assessment for Lean body mass

Appendix 5

Primary Outcome

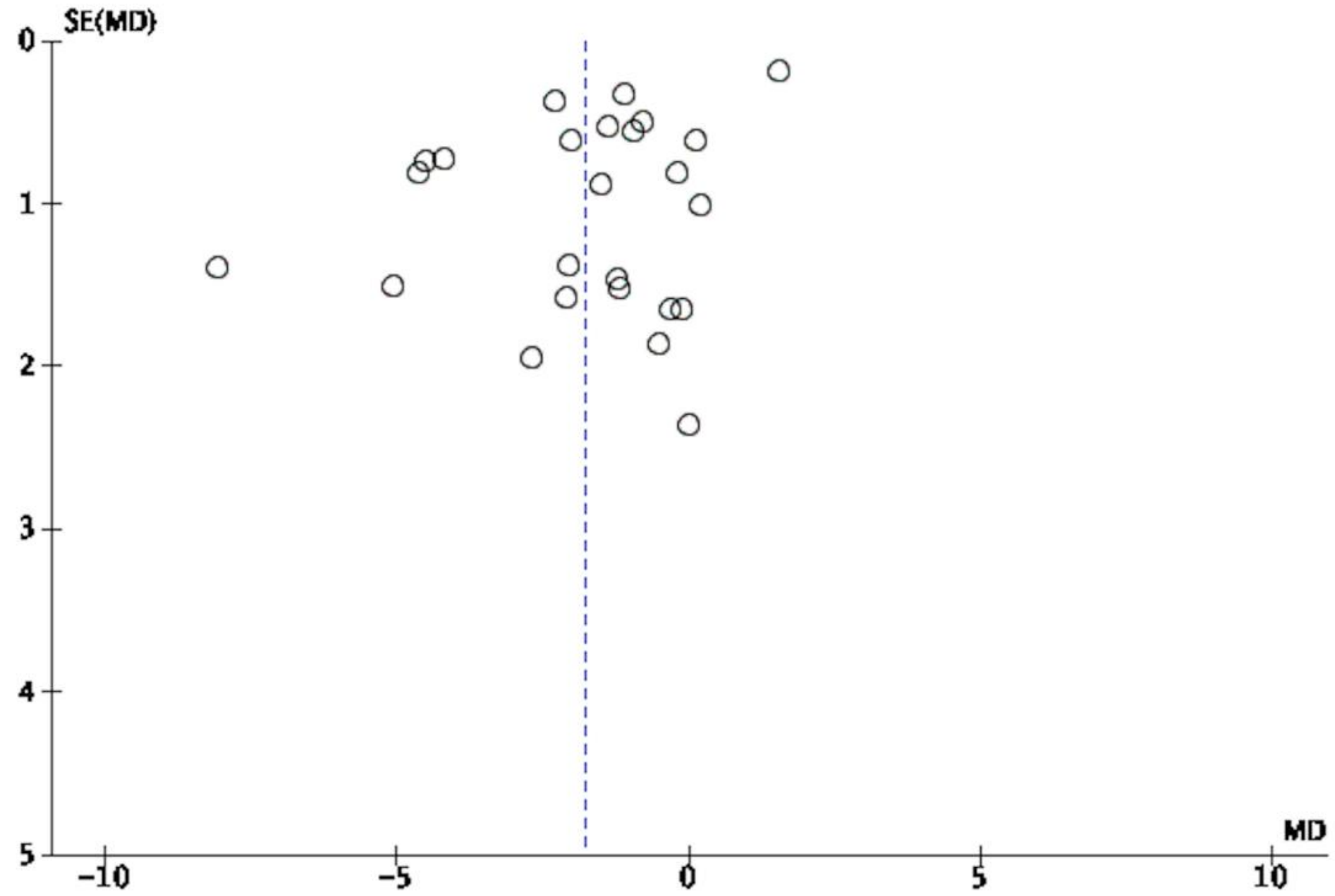

F 1 Funnel plot for the meta-analysis of BMI

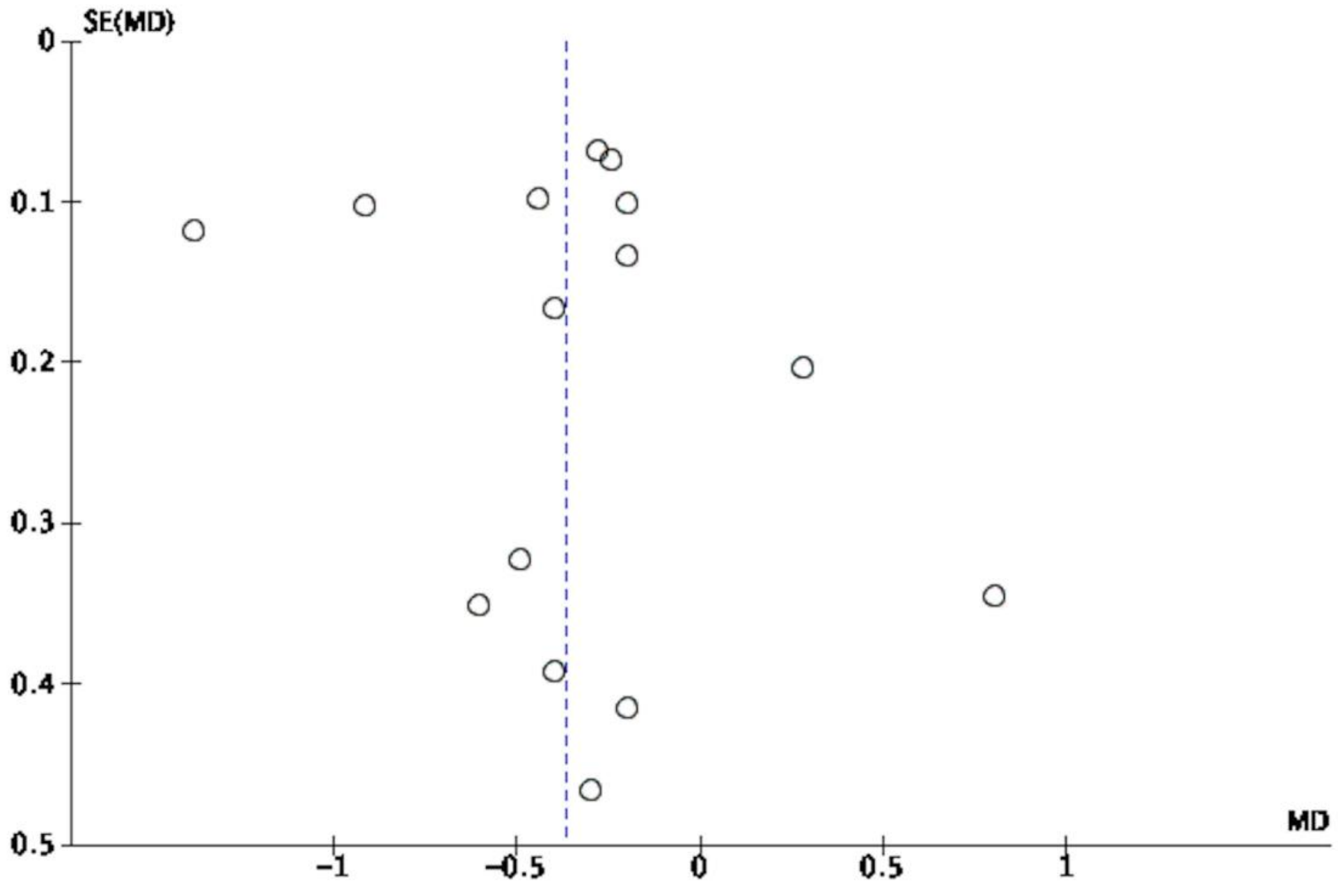

F 2 Funnel plot for the meta-analysis of TC

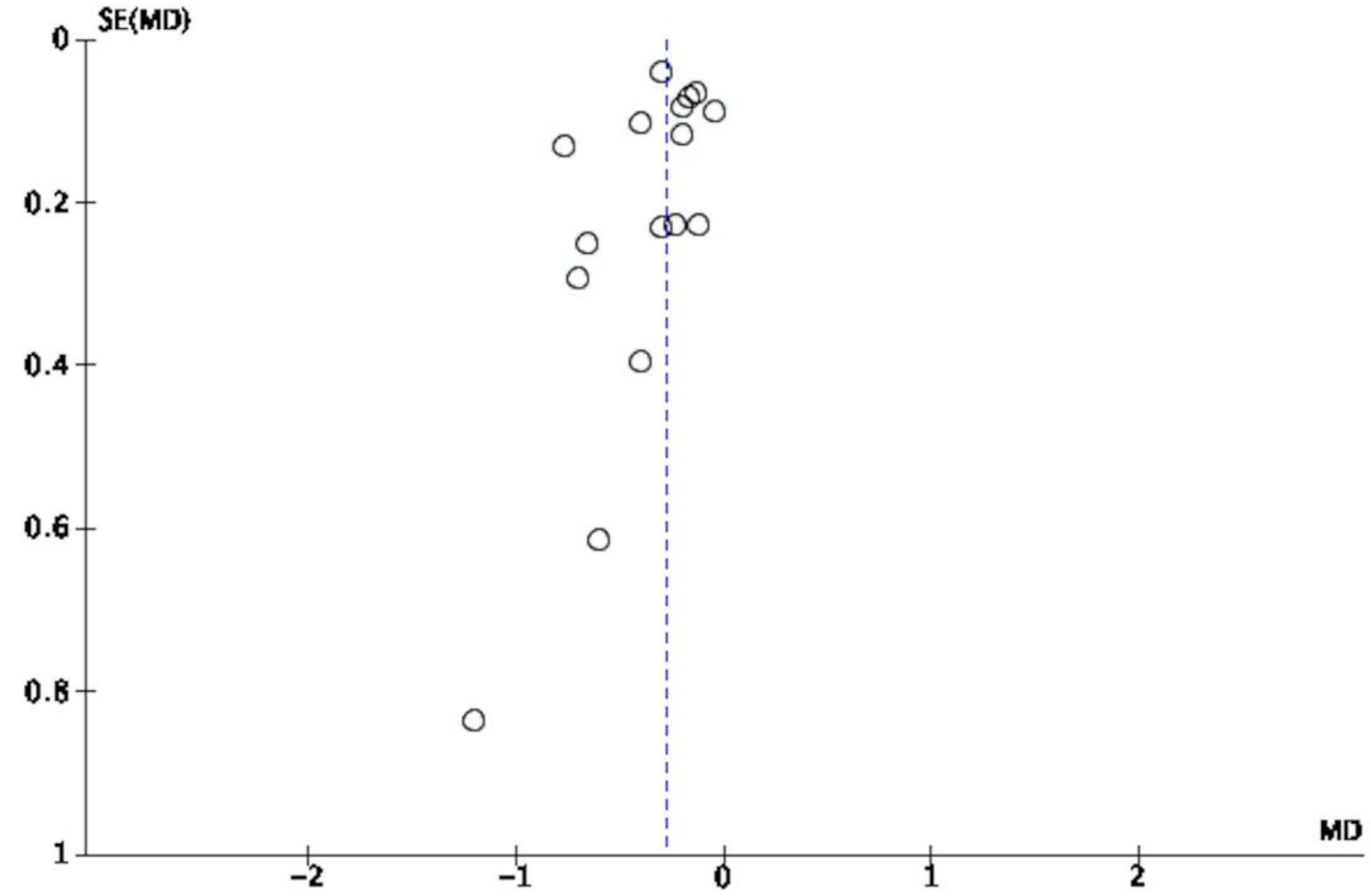

F 3 Funnel plot for the meta-analysis of TG

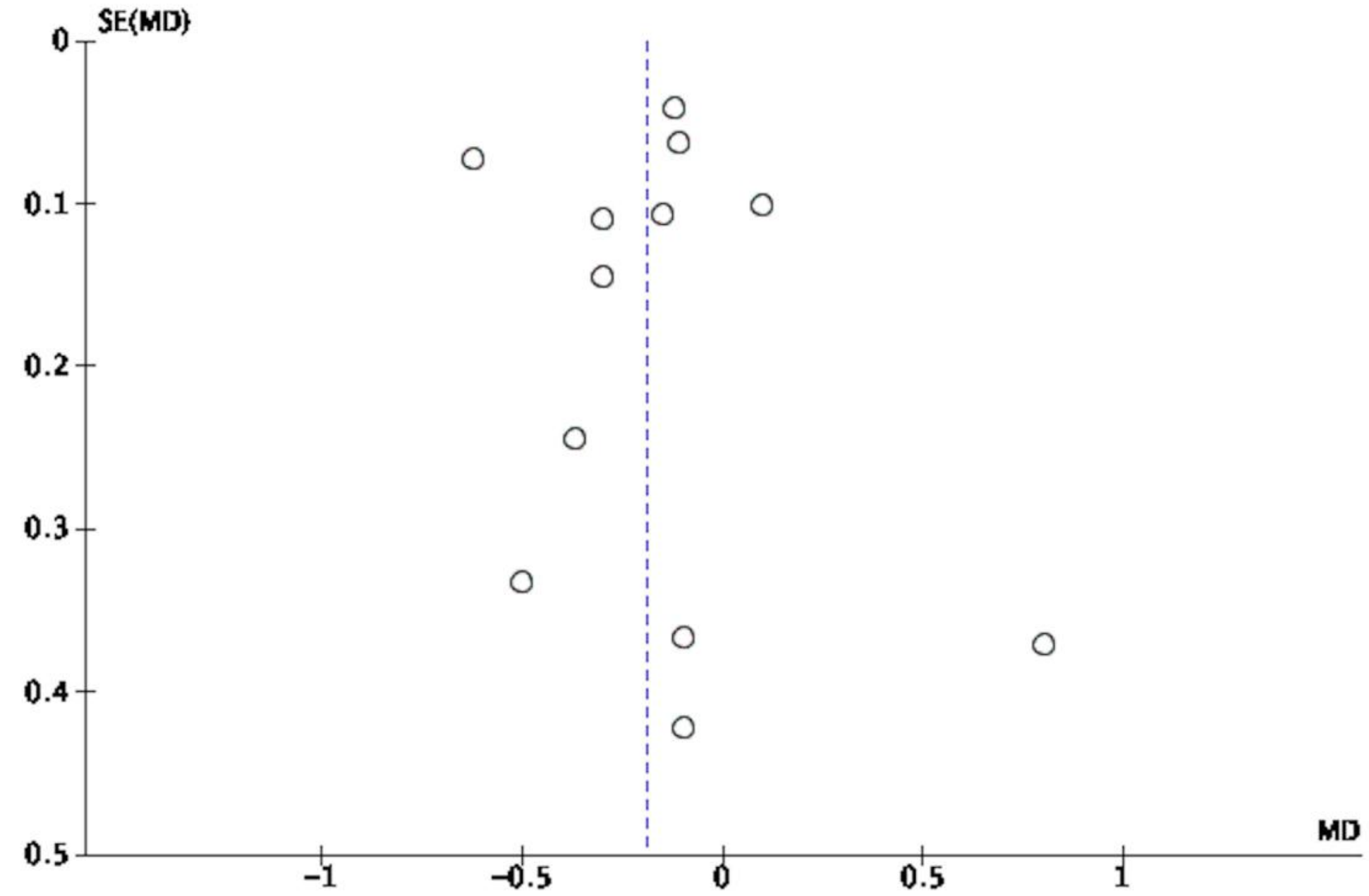

F 4 Funnel plot for the meta-analysis of LDL-C

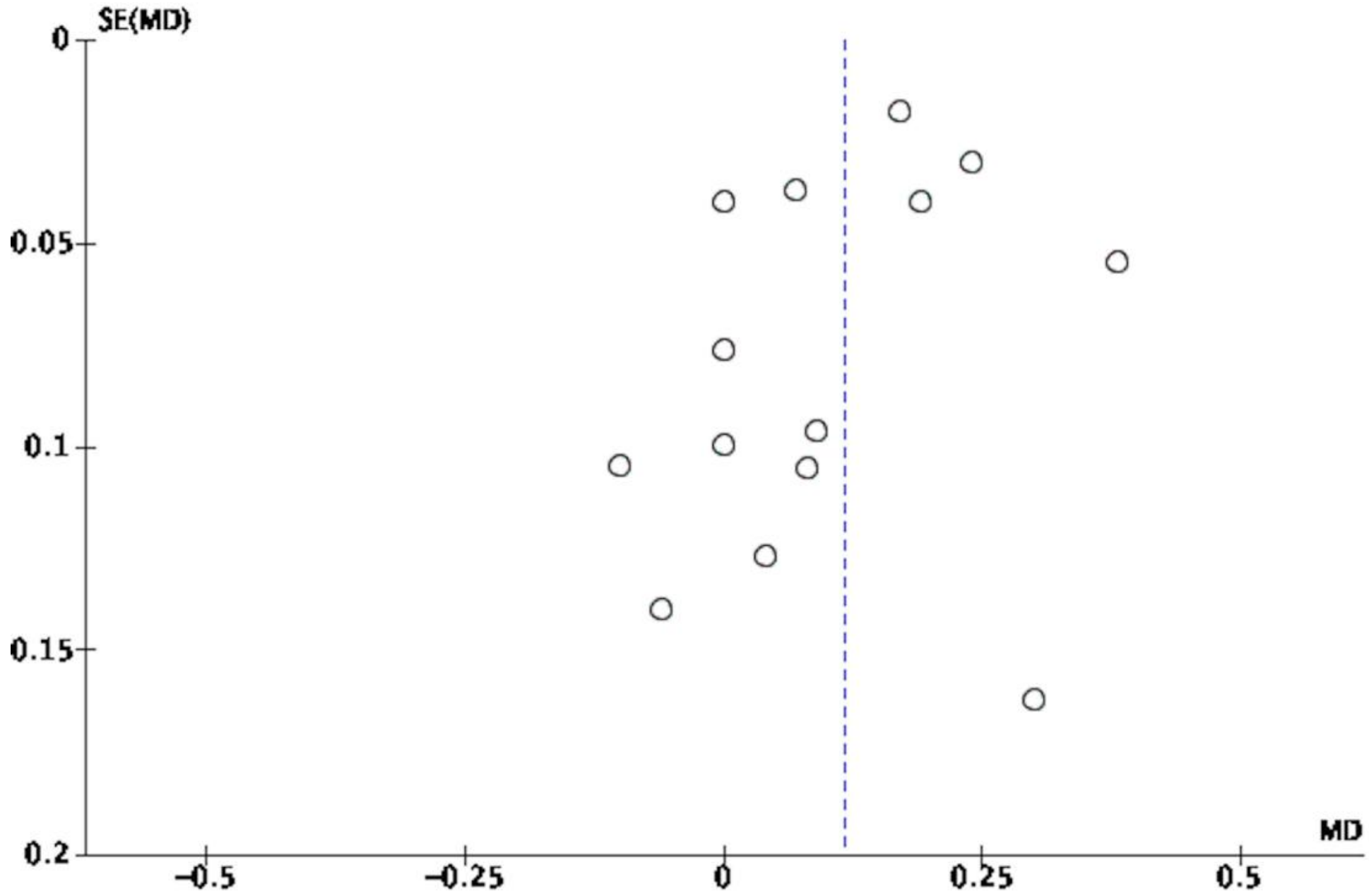

F 5 Funnel plot for the meta-analysis of HDL-C

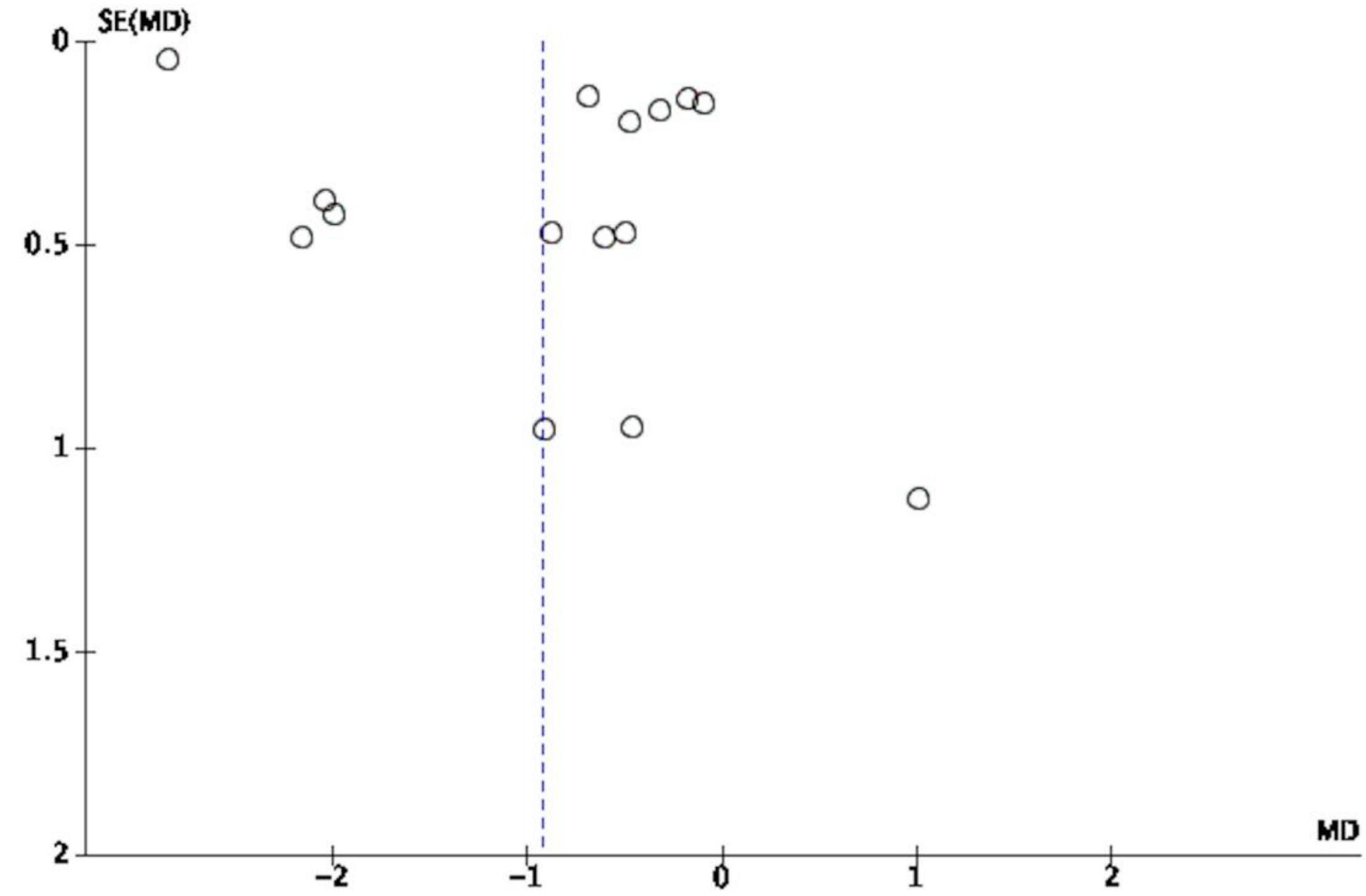

F 6 Funnel plot for the meta-analysis of HOMA-IR

Secondary Outcome

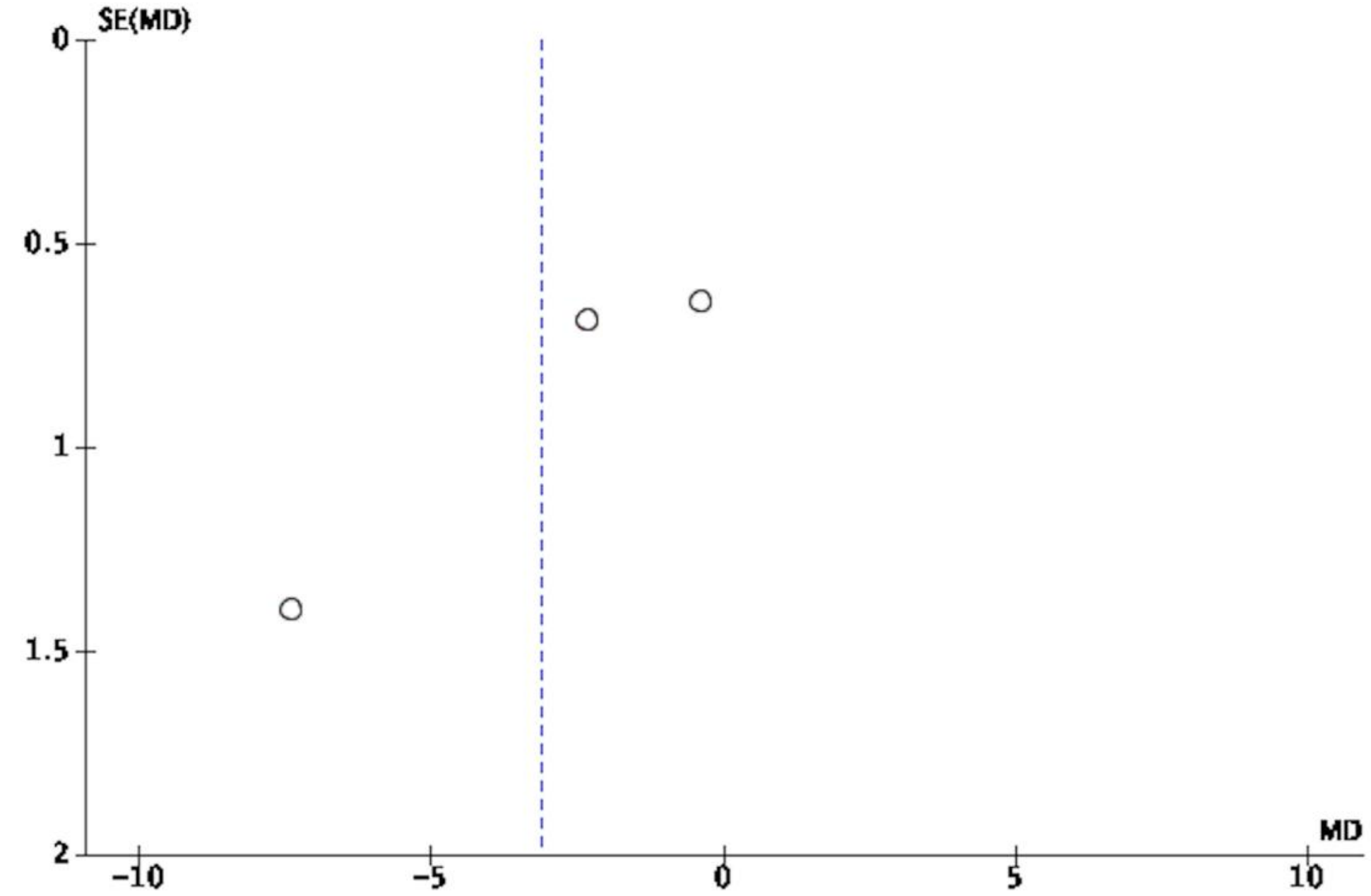

F 7 Funnel plot for the meta-analysis of IL-6

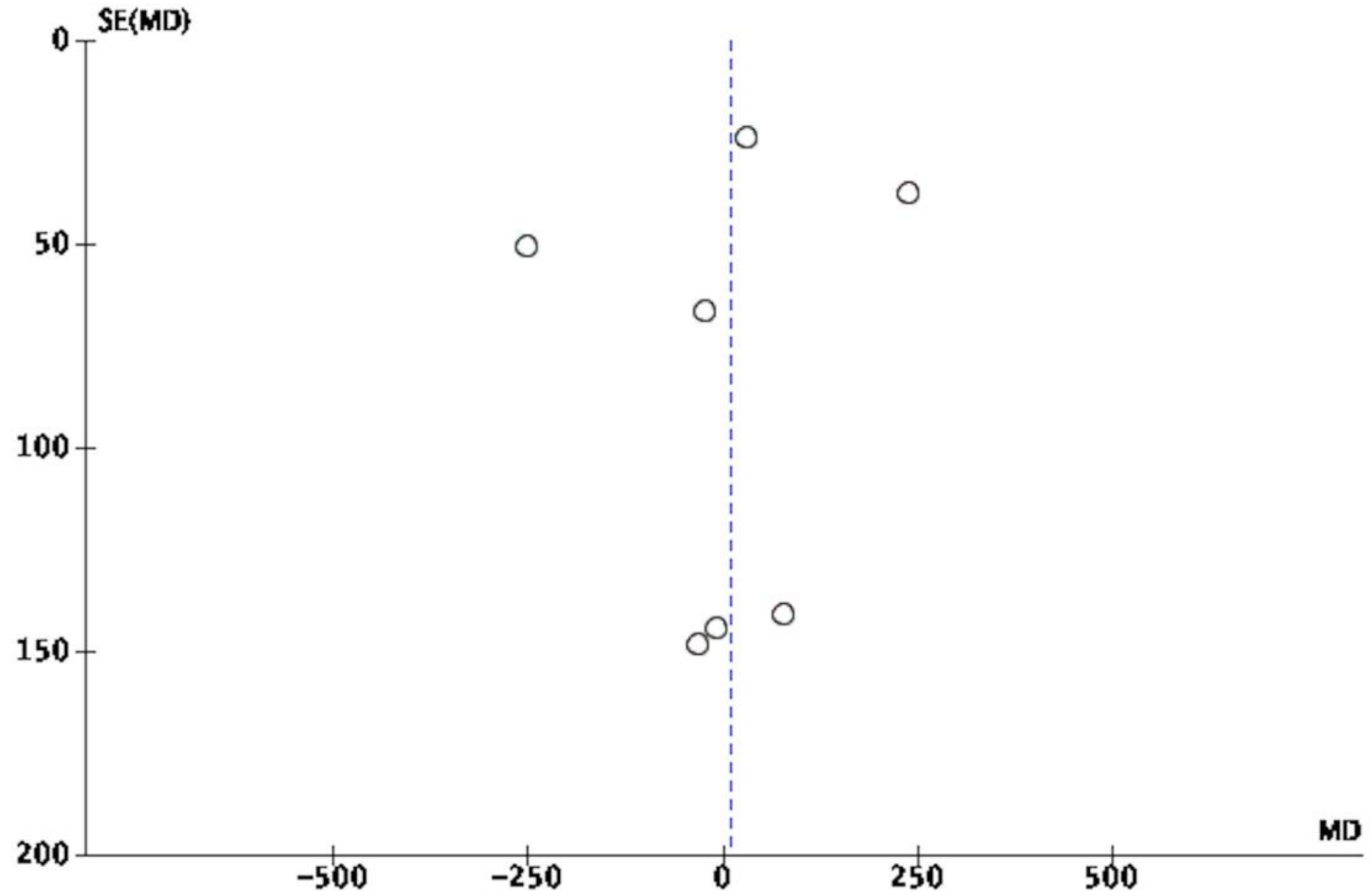

F 8 Funnel plot for the meta-analysis of Total intake

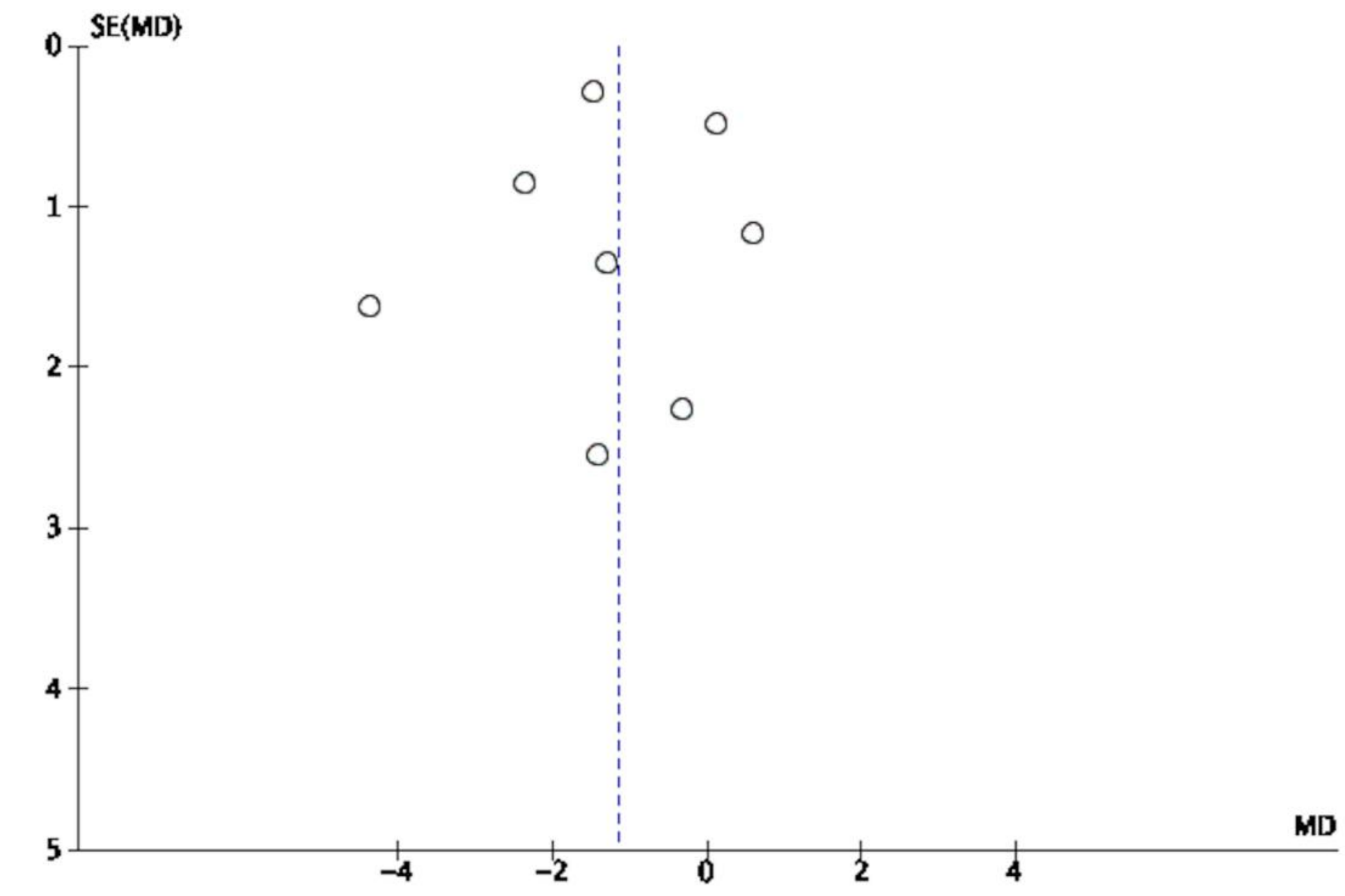

F 9 Funnel plot for the meta-analysis of Insulin

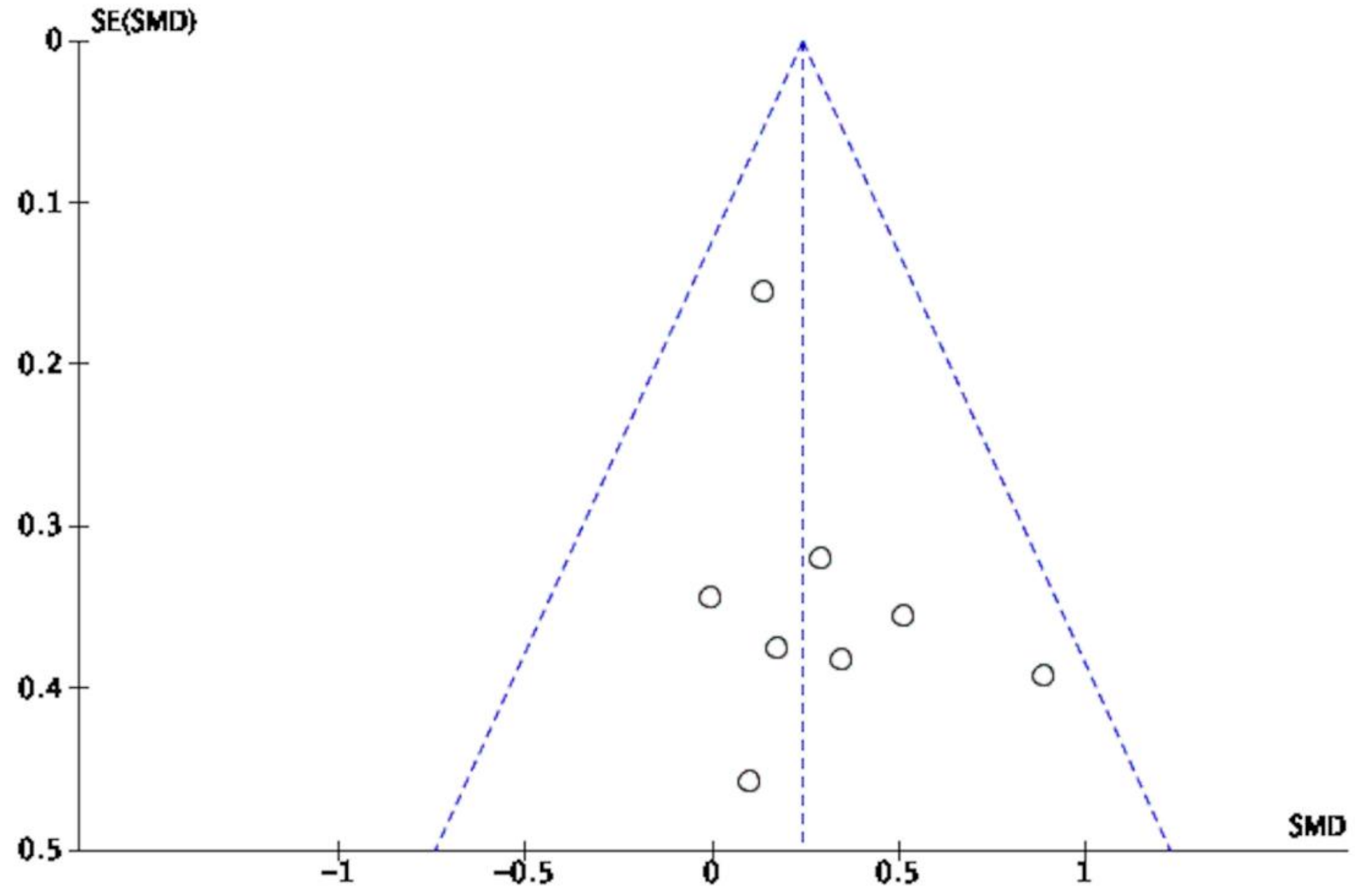

F 10 Funnel plot for the meta-analysis of Lean body mass

Appendix 6

Primary Outcome

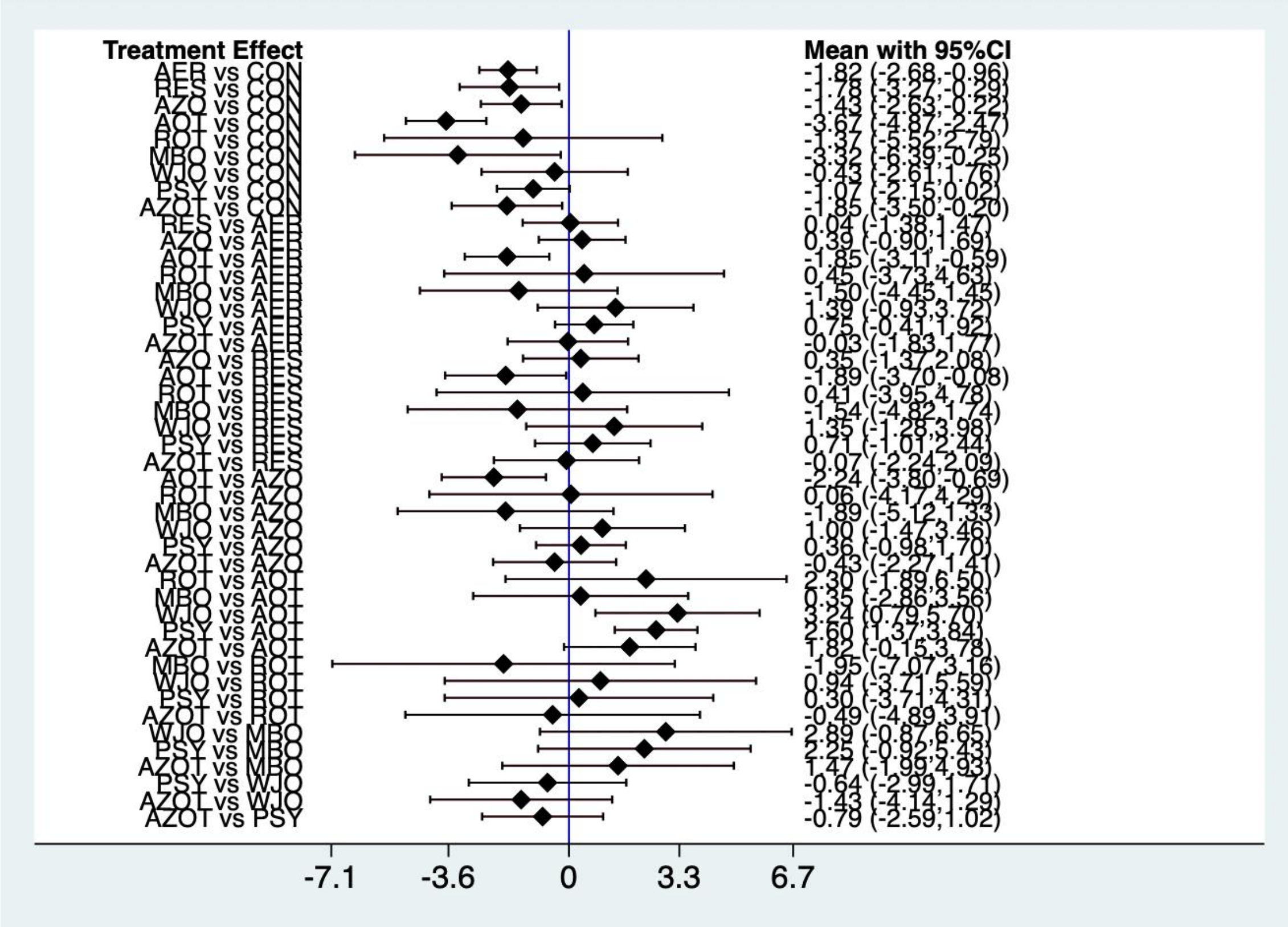

F 1 BMI's mesh Meta two-by-two comparison chart

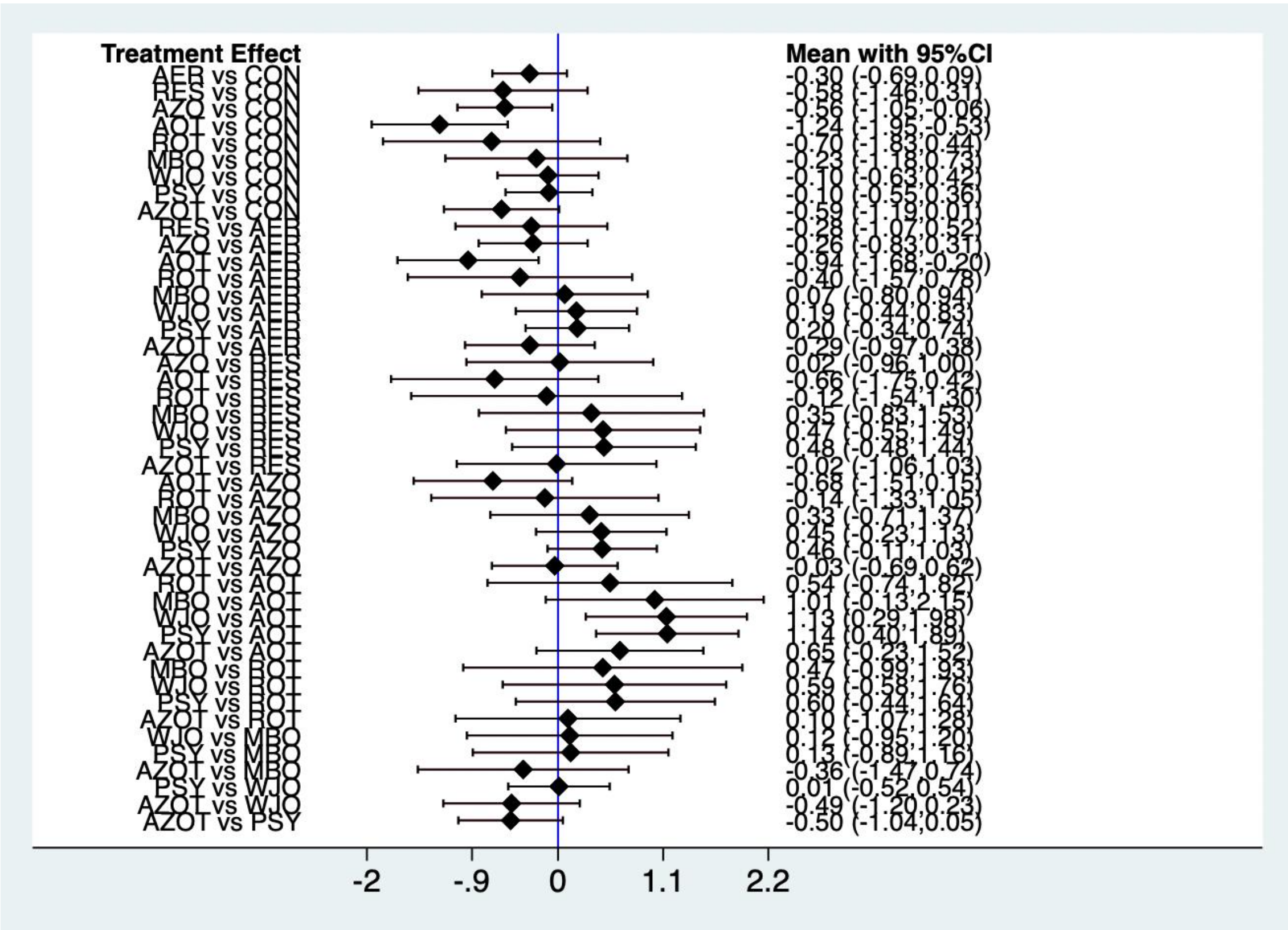

F 2 TC's mesh Meta two-by-two comparison chart

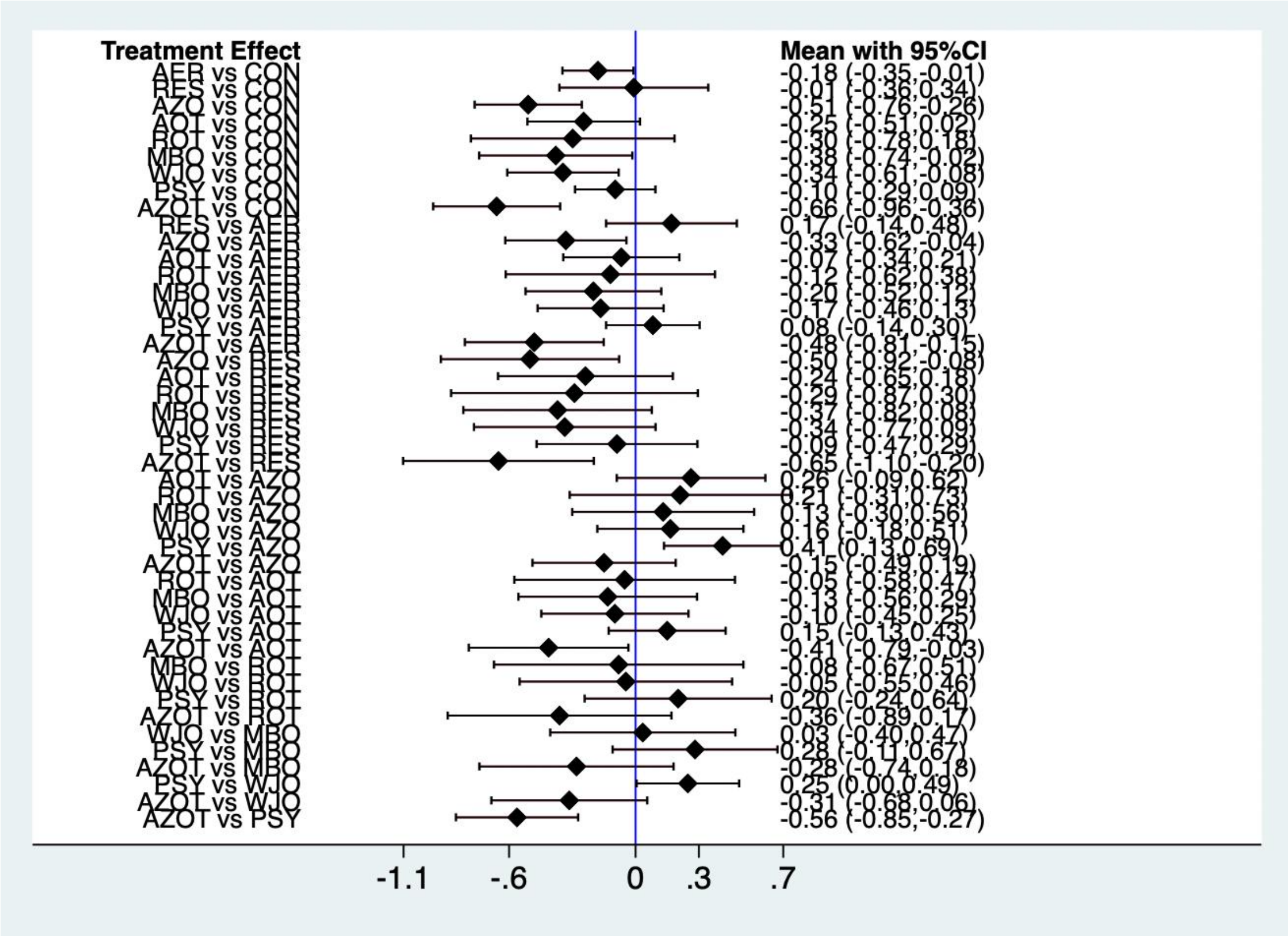

F 3 TG's mesh Meta two-by-two comparison chart

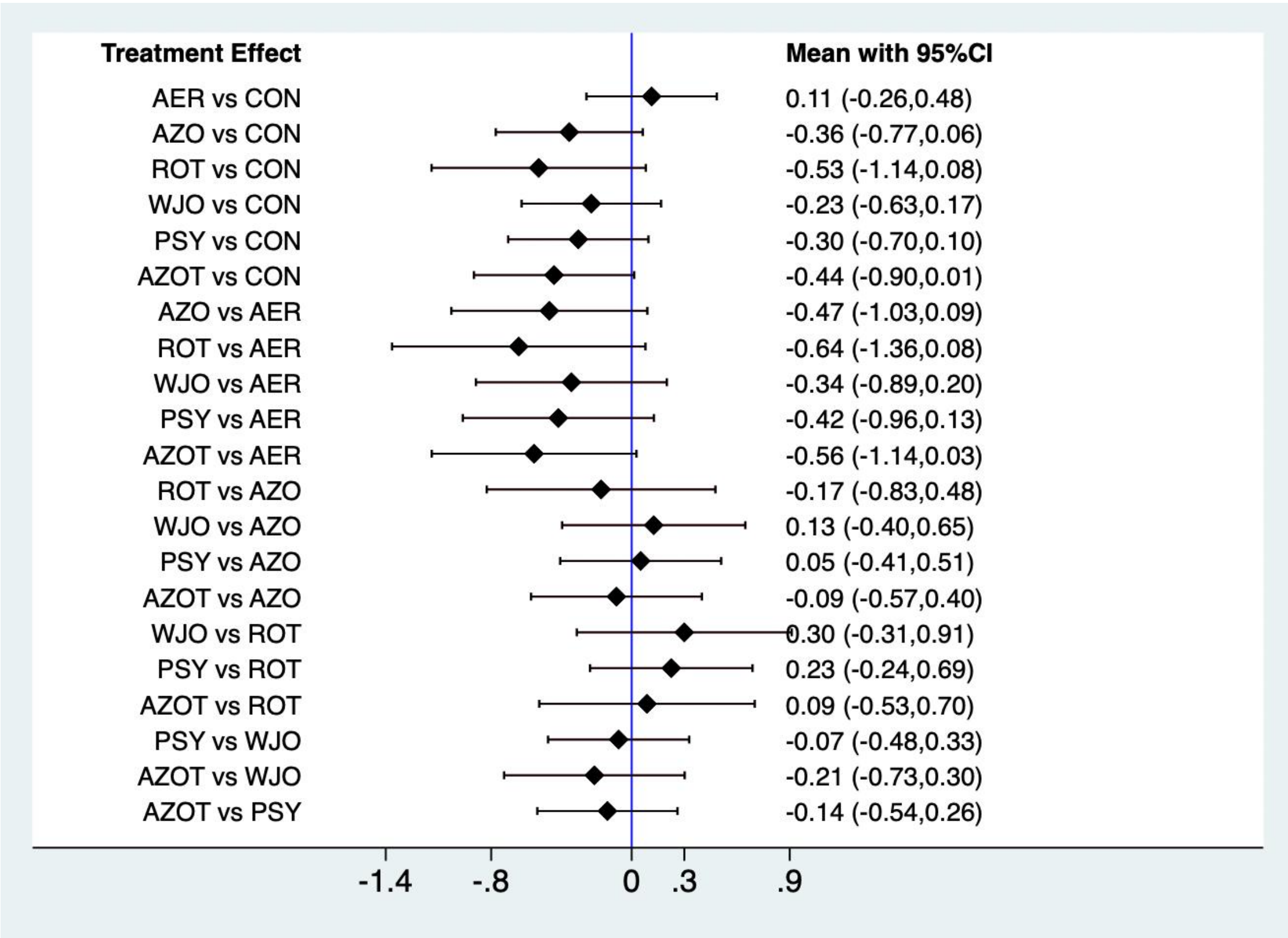

F 4 LDL-C's mesh Meta two-by-two comparison chart

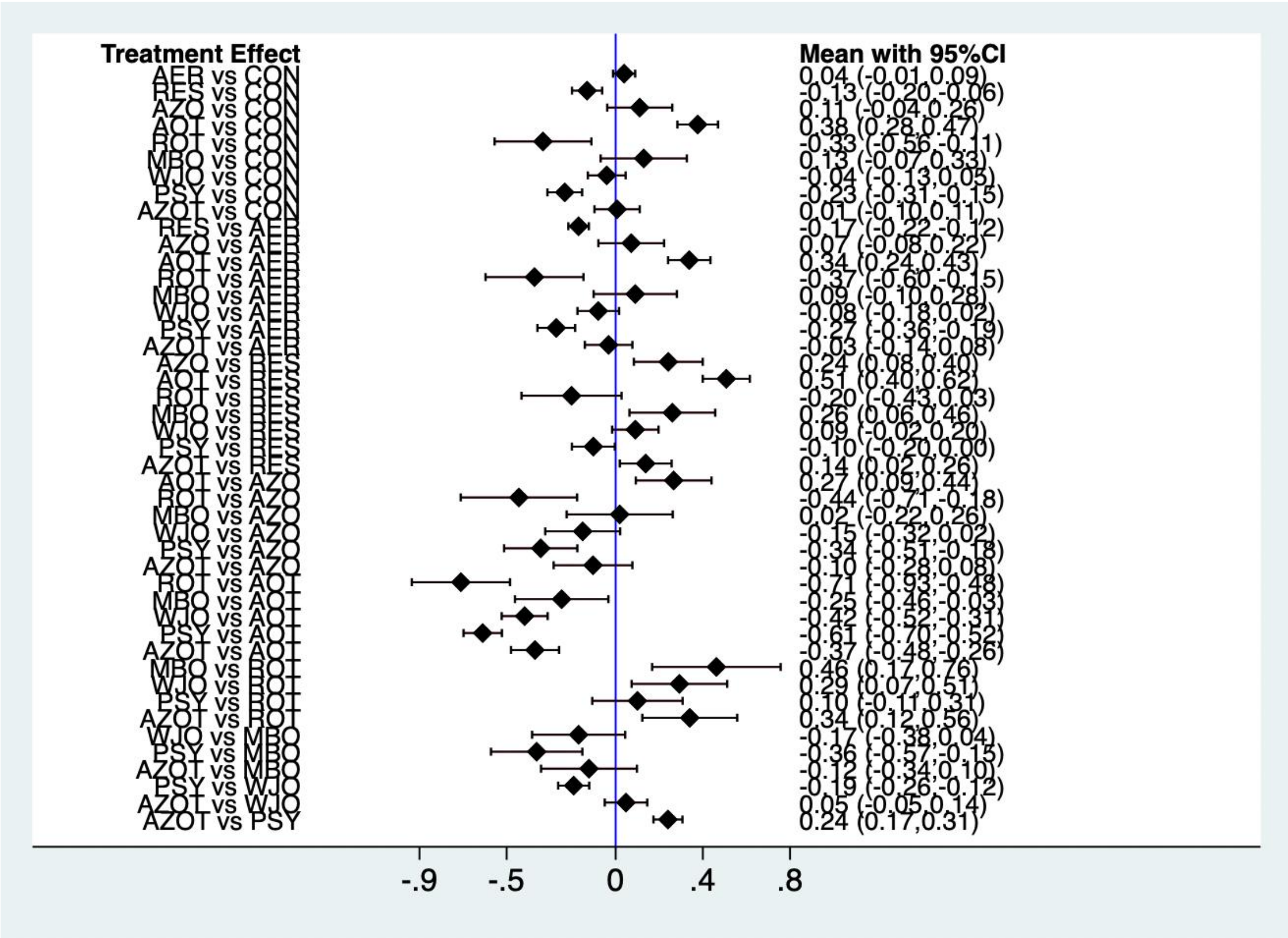

F 5 HDL-C's mesh Meta two-by-two comparison chart

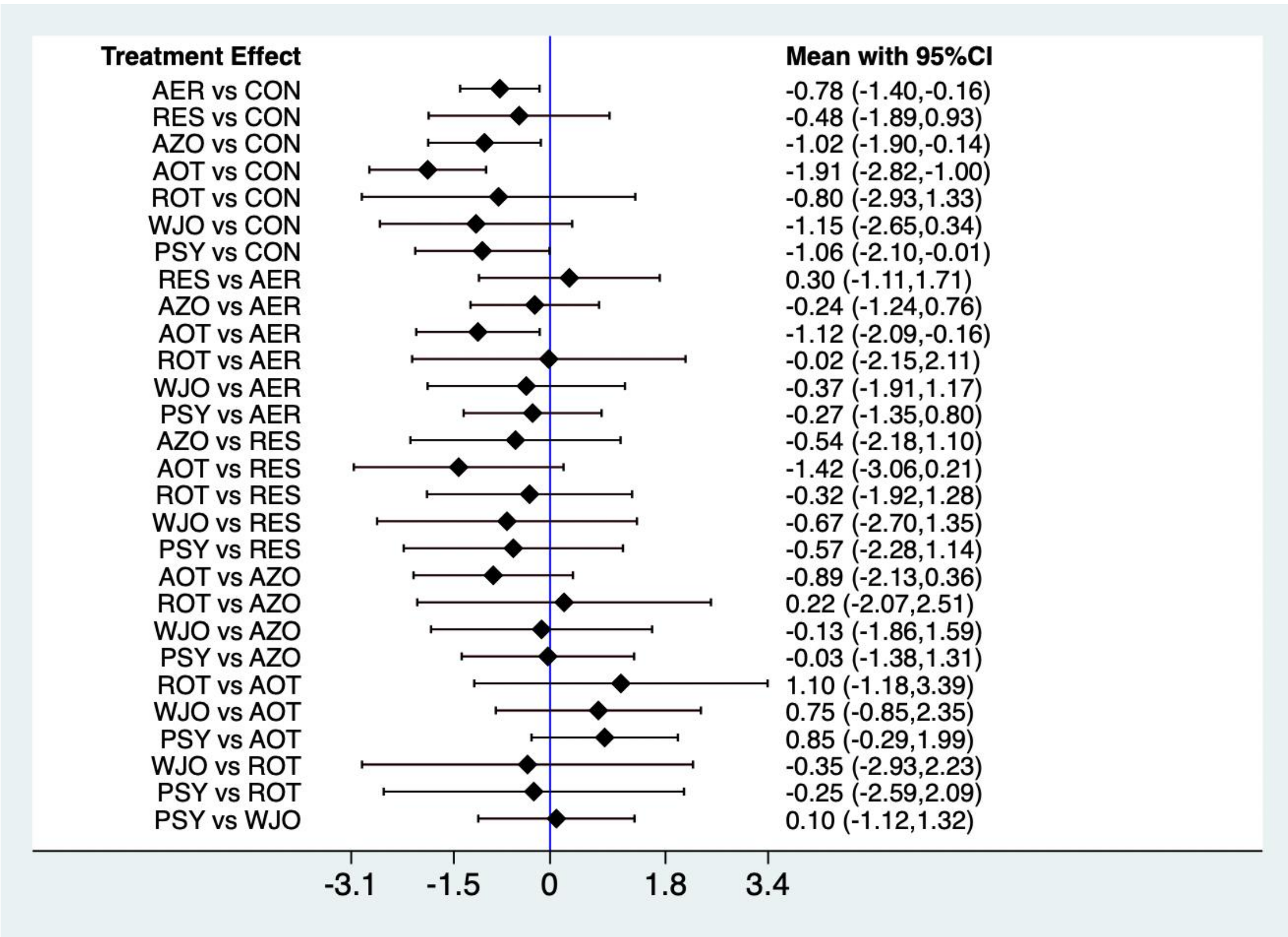

F 6 HOMA-IR's mesh Meta two-by-two comparison chart

Secondary Outcome

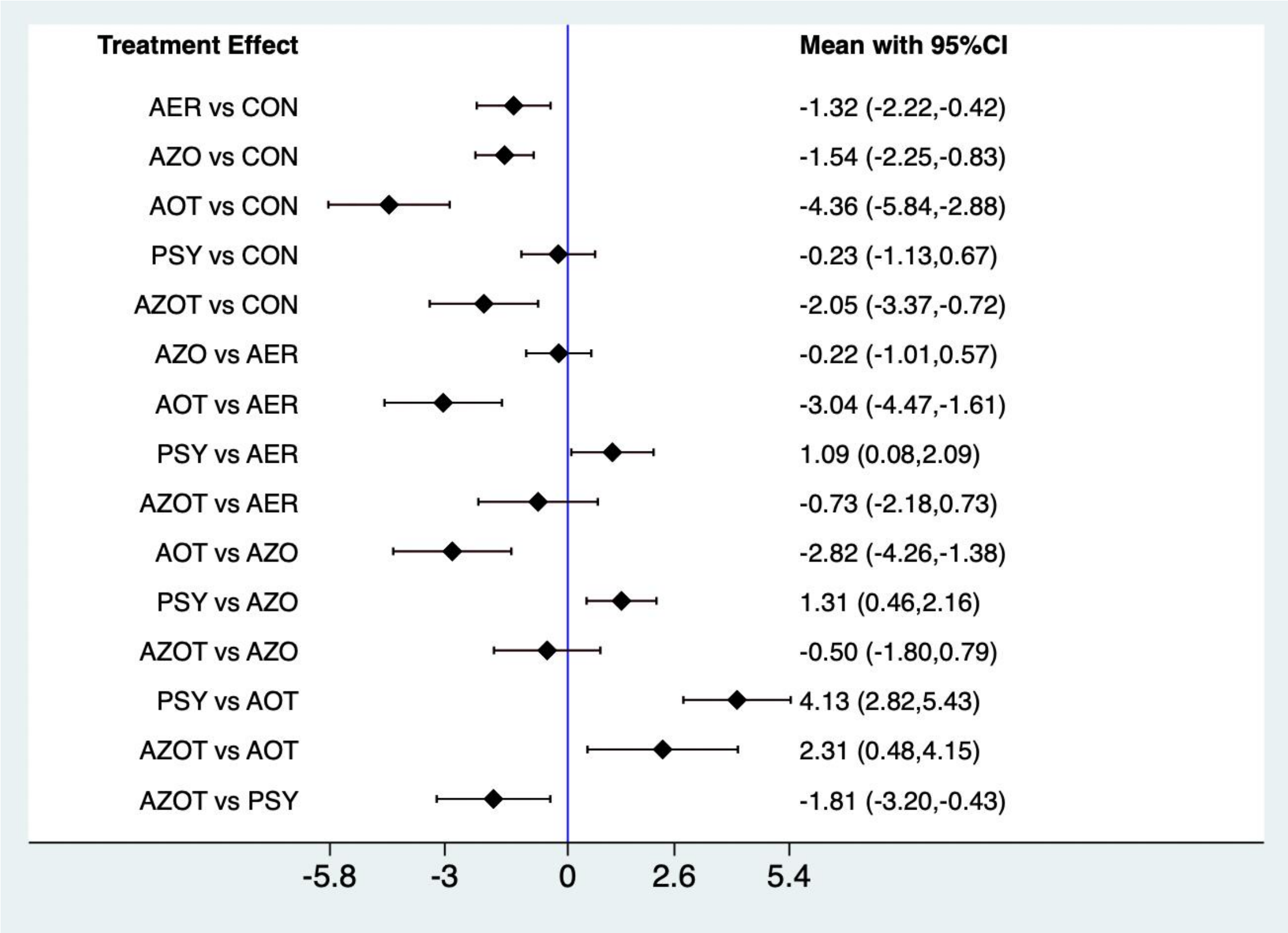

F 7 IL-6's mesh Meta two-by-two comparison chart

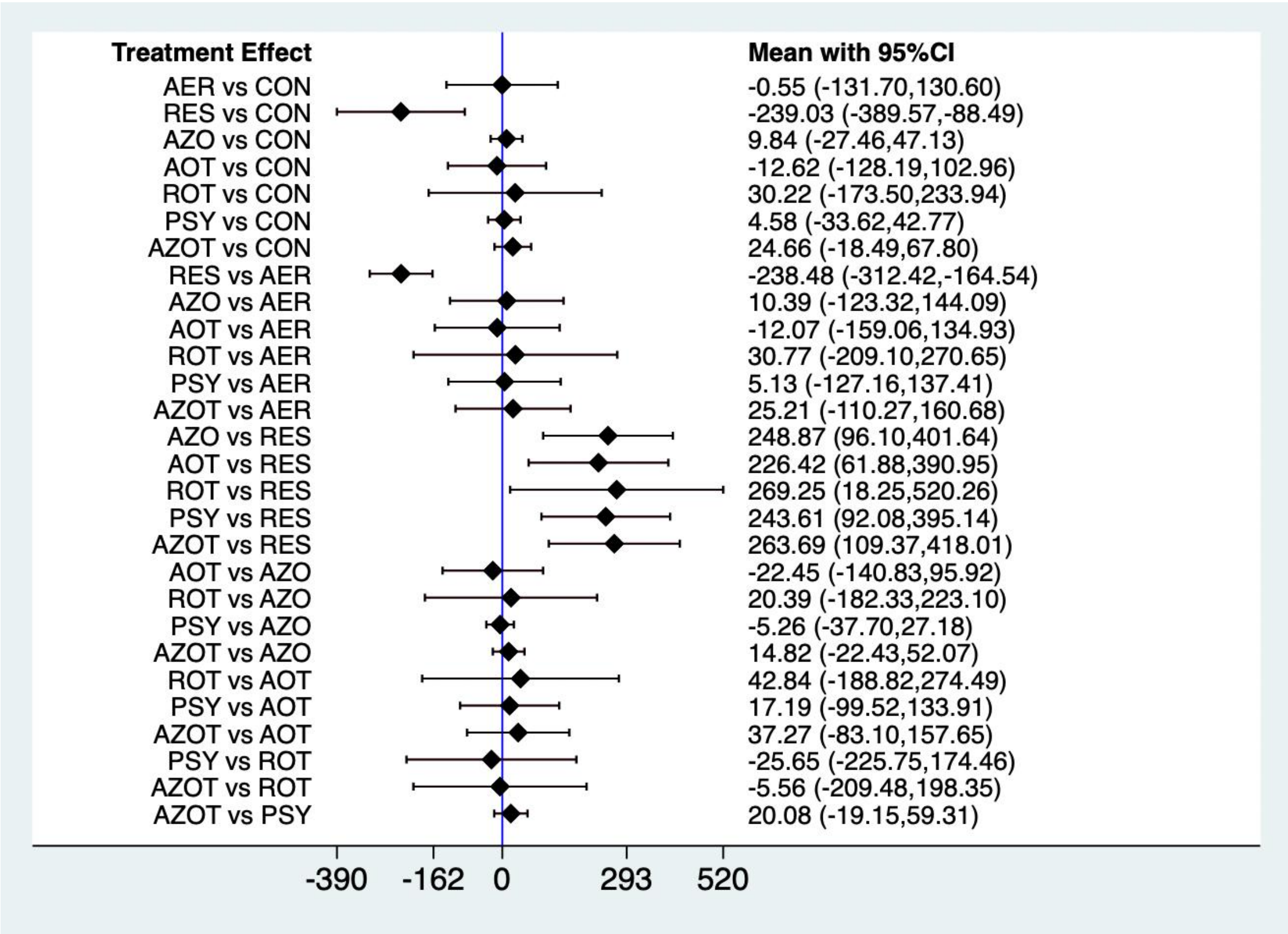

F 8 Total intake's mesh Meta two-by-two comparison chart

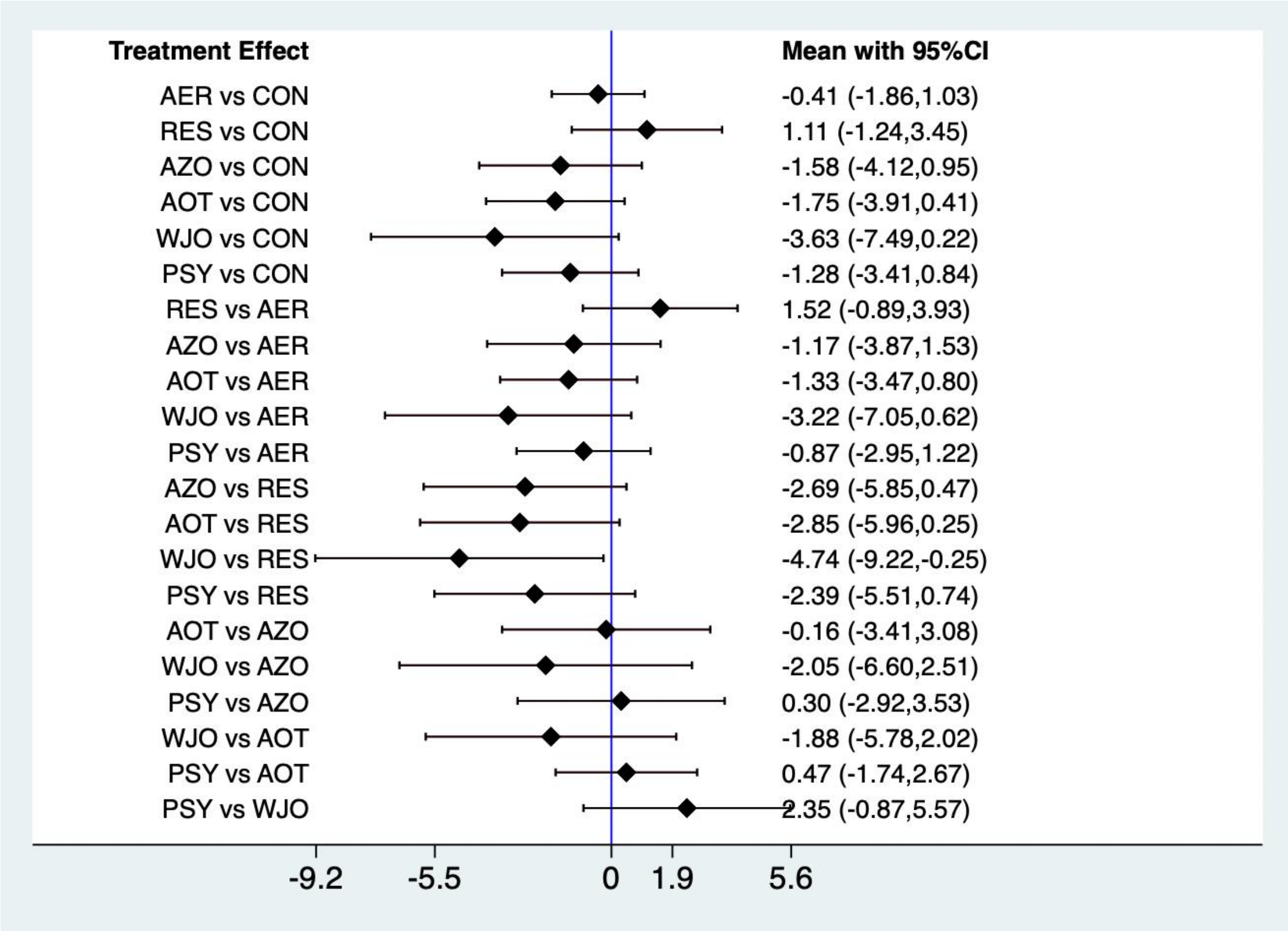

F 9 Insulin's mesh Meta two-by-two comparison chart

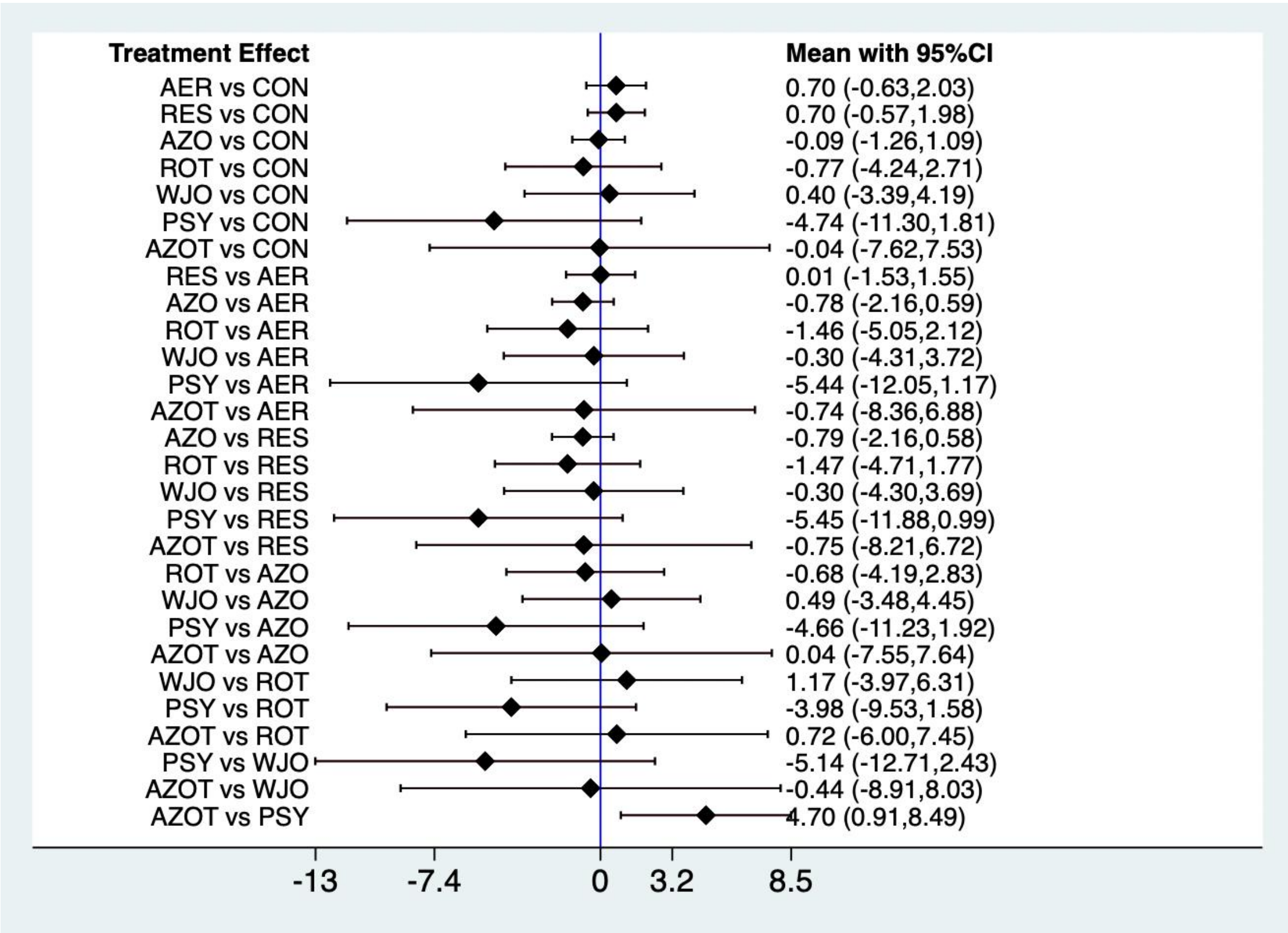

F 10 Lean body mass's mesh Meta two-by-two comparison chart

Note: Blank control group (CON), Aerobic exercise only (AER), Resistance exercise only(RES), Aerobic plus resistance exercise (AZO), Aerobic exercise combined with other effective treatments (AOT), Resistance

exercise combined with other effective treatments (ROT), Mind-body exercise group (MBO), Walking and jogging group (WJO), Pharmacological or psychological group(PSY), Aerobic exercise plus resistance exercise combined with other effective treatments(AZTO).

Appendix 7
Primary Outcome

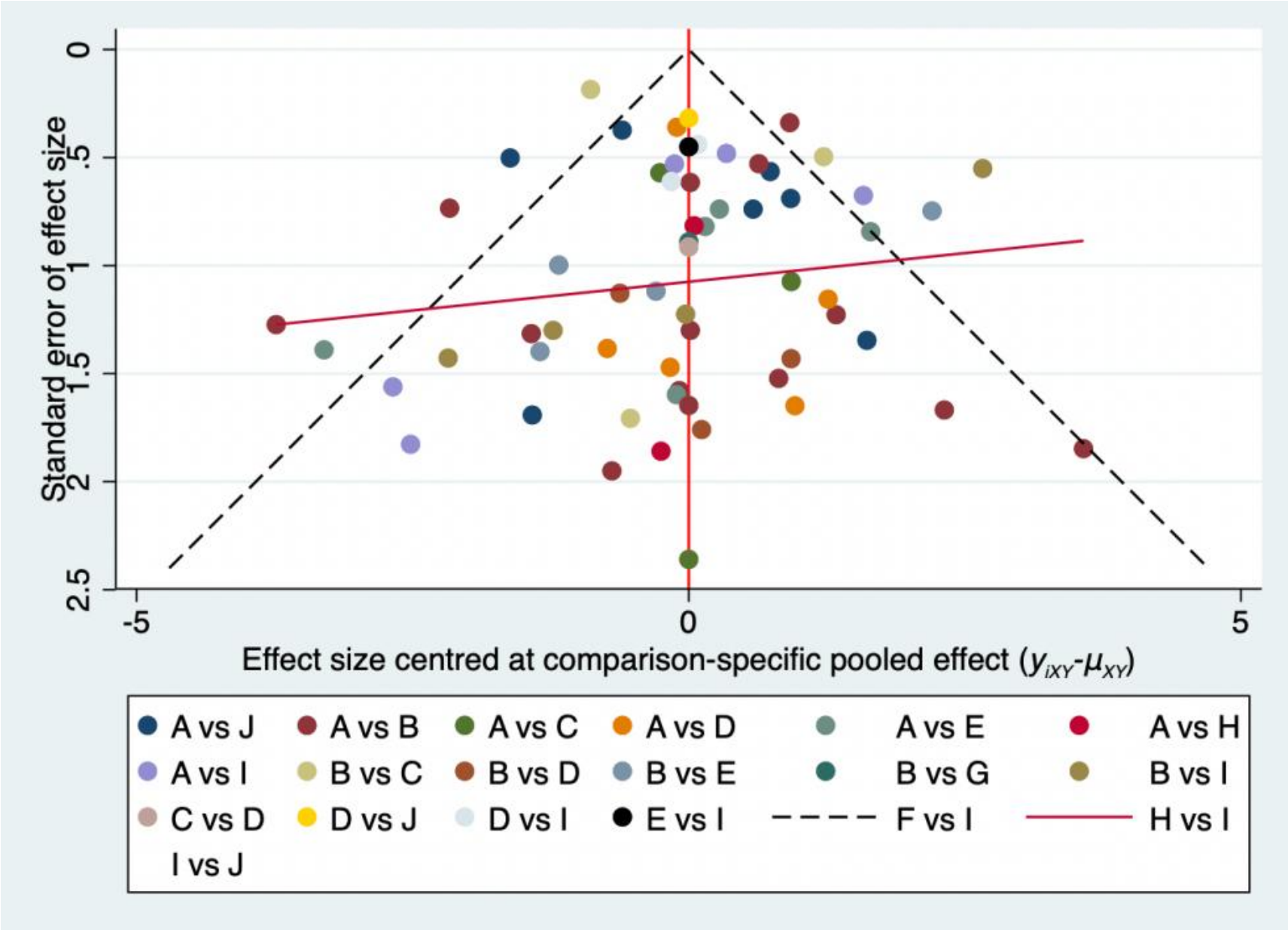

F 1 Comparison-adjusted funnel plot for the network meta-analysis of BMI

Note: Blank control group (A), Aerobic exercise only (B), Resistance exercise only(C), Aerobic plus resistance exercise (D), Aerobic exercise combined with other effective treatments (E), Resistance exercise combined with other effective treatments (F), Mind-body exercise group (G), Walking and jogging group (H), Pharmacological or psychological group(I), Aerobic exercise plus resistance exercise combined with other effective treatments(J).

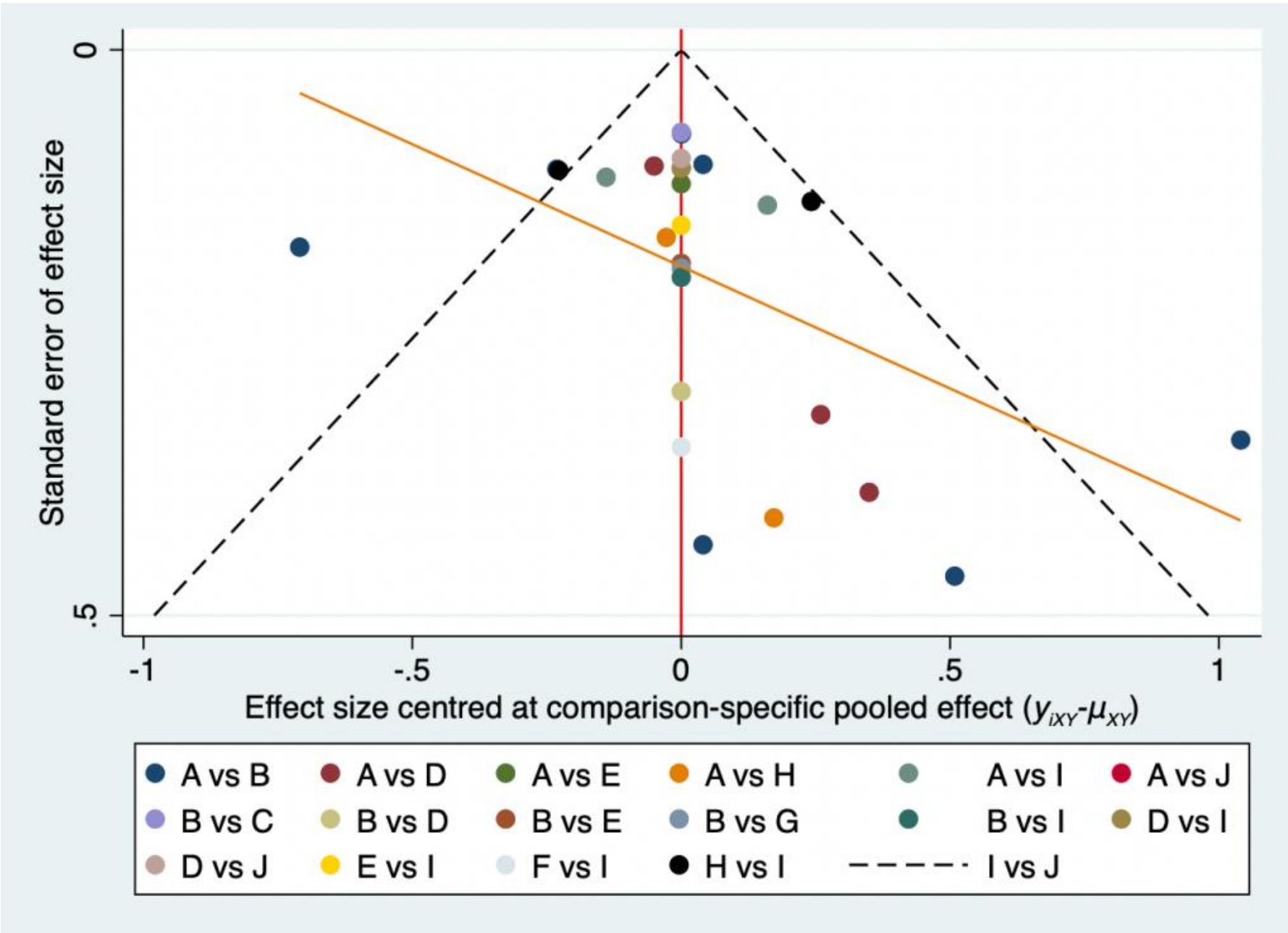

F 2 Comparison-adjusted funnel plot for the network meta-analysis of TC

Note: Blank control group (A), Aerobic exercise only (B), Resistance exercise only(C), Aerobic plus resistance exercise (D), Aerobic exercise combined with other effective treatments (E), Resistance exercise combined with other effective treatments (F), Mind-body exercise group (G), Walking and jogging group (H), Pharmacological or psychological group(I), Aerobic exercise plus resistance exercise combined with other effective treatments(J).

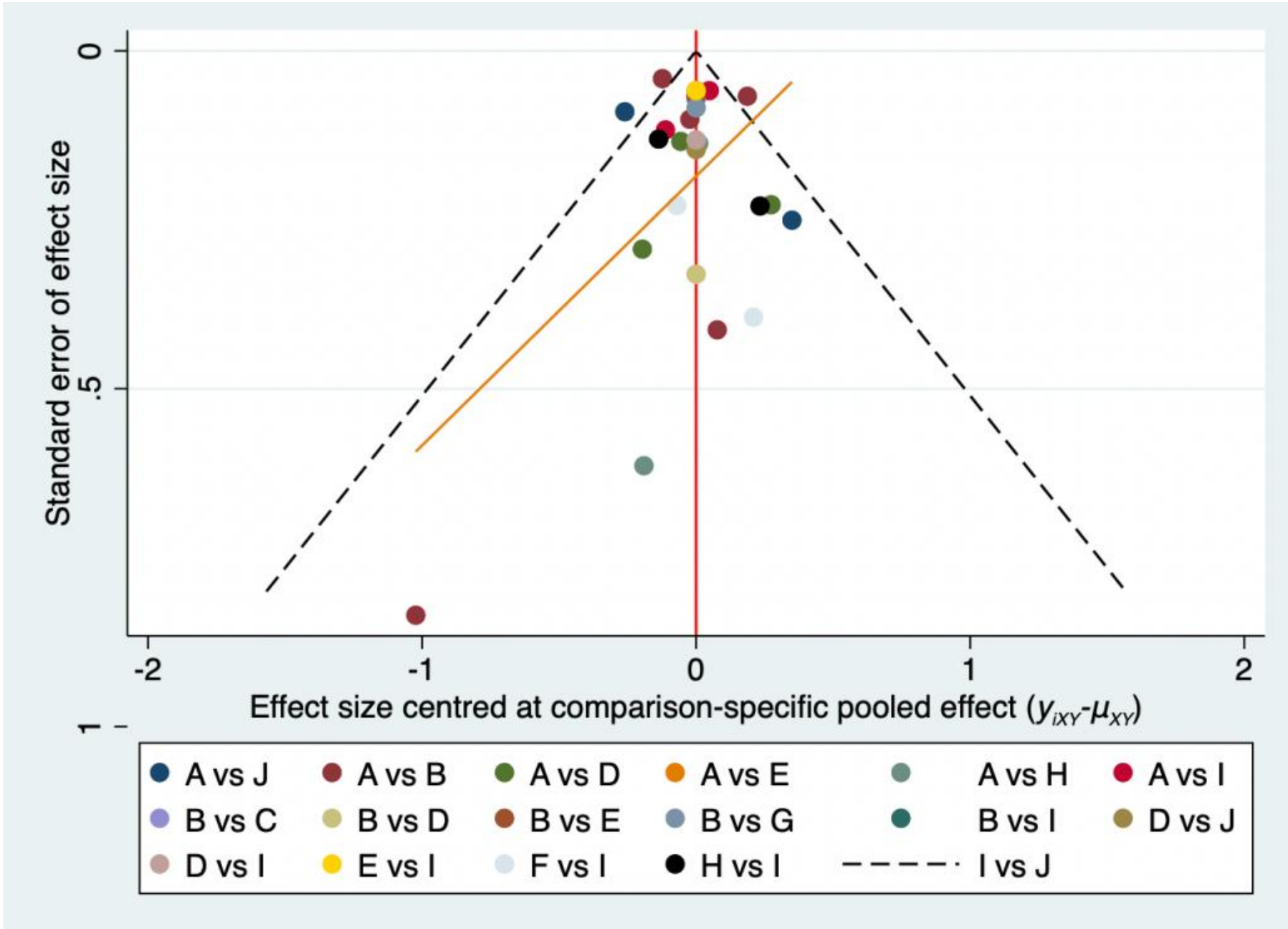

F 3 Comparison-adjusted funnel plot for the network meta-analysis of TG

Note: Blank control group (A), Aerobic exercise only (B), Resistance exercise only(C), Aerobic plus resistance exercise (D), Aerobic exercise combined with other effective treatments (E), Resistance exercise combined with other effective treatments (F), Mind-body exercise group (G), Walking and jogging group (H), Pharmacological or psychological group(I), Aerobic exercise plus resistance exercise combined with other effective treatments(J).

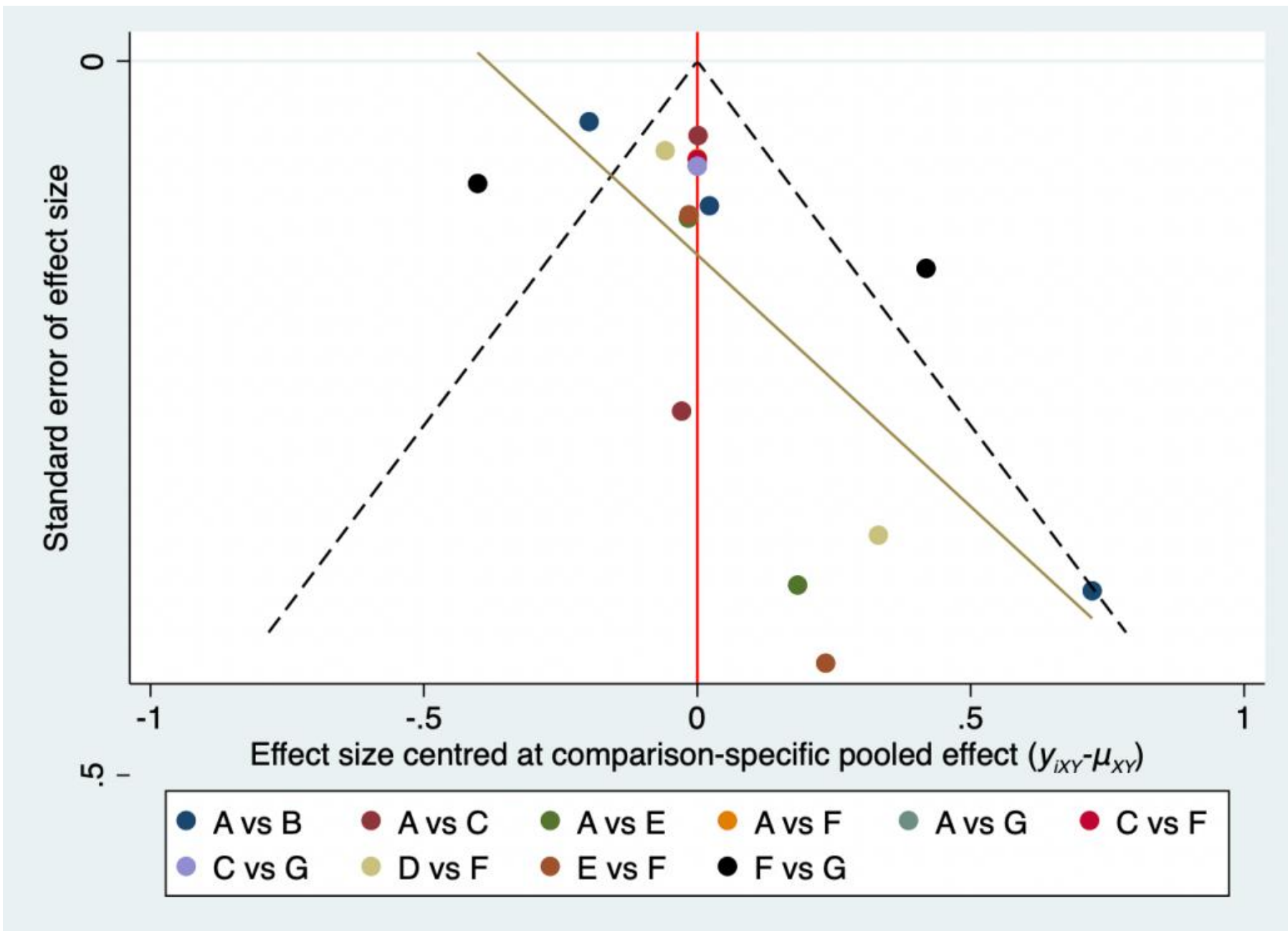

F 4 Comparison-adjusted funnel plot for the network meta-analysis of LDL-C

Note: Blank control group (A), Aerobic exercise only (B), Aerobic plus resistance exercise (C), Resistance exercise combined with other effective treatments (D), Walking and jogging group (E), Pharmacological or psychological group(F), Aerobic exercise plus resistance exercise combined with other effective treatments(G).

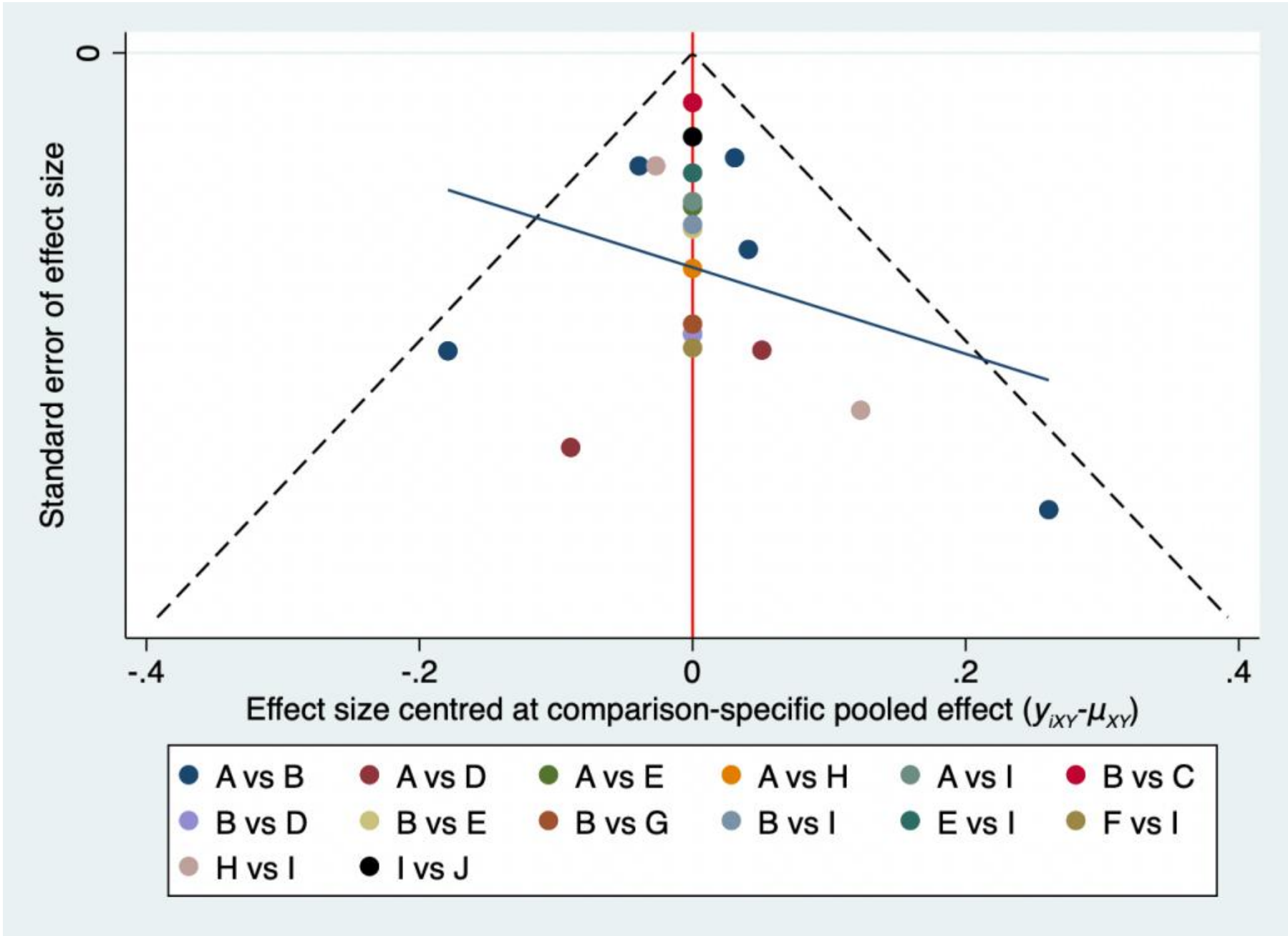

F 5 Comparison-adjusted funnel plot for the network meta-analysis of HDL-C

Note: Blank control group (A), Aerobic exercise only (B), Resistance exercise only(C), Aerobic plus resistance exercise (D), Aerobic exercise combined with other effective treatments (E), Resistance exercise combined with other effective treatments (F), Mind-body exercise group (G), Walking and jogging group (H), Pharmacological or psychological group(I), Aerobic exercise plus resistance exercise combined with other effective treatments(J).

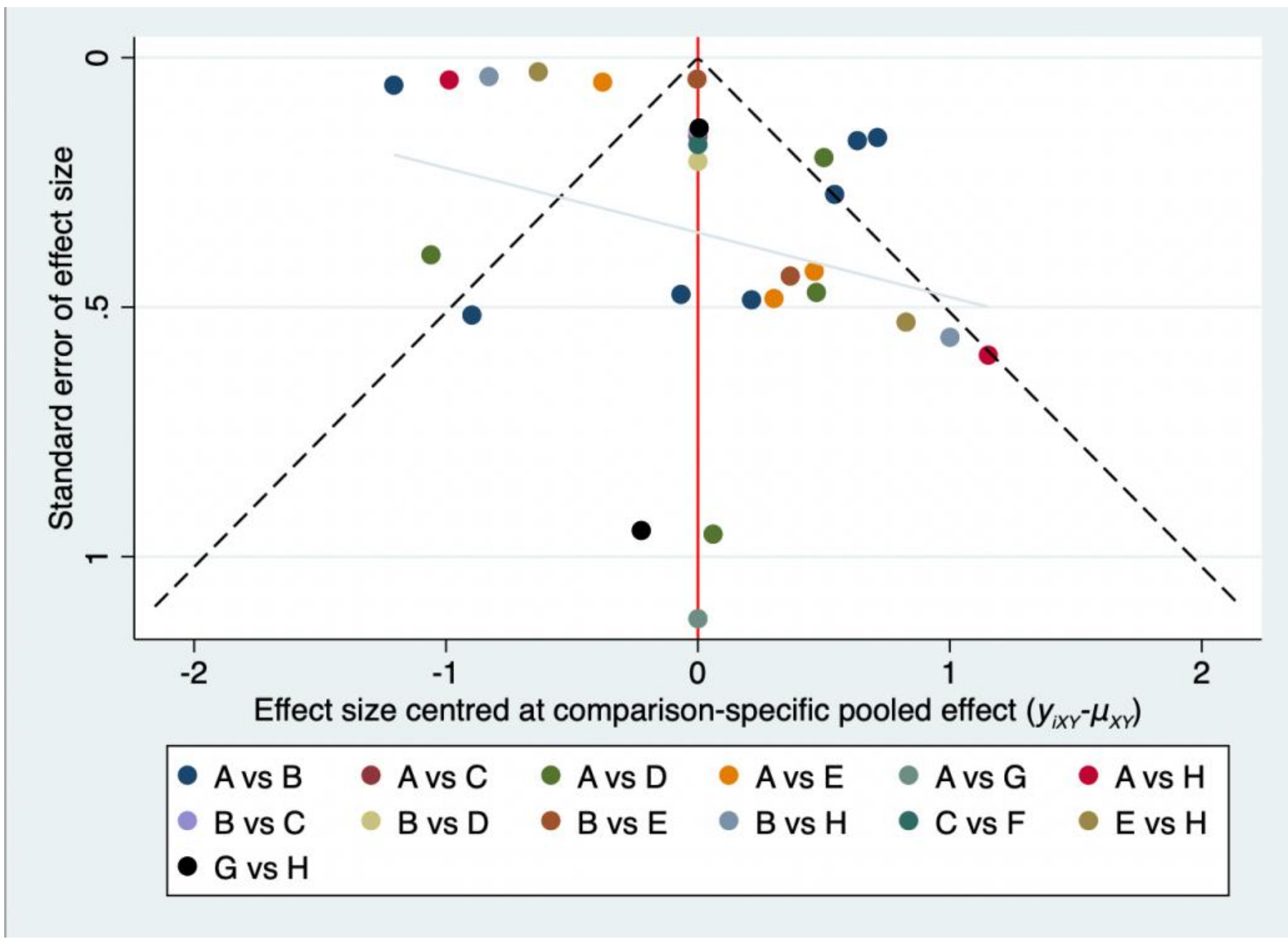

F 6 Comparison-adjusted funnel plot for the network meta-analysis of HOMA-IR

Note: Blank control group (A), Aerobic exercise only (B), Resistance exercise only(C), Aerobic plus resistance exercise (D), Aerobic exercise combined with other effective treatments (E), Resistance exercise combined with other effective treatments (F), Walking and jogging group (G), Pharmacological or psychological group(H).

Secondary Outcome

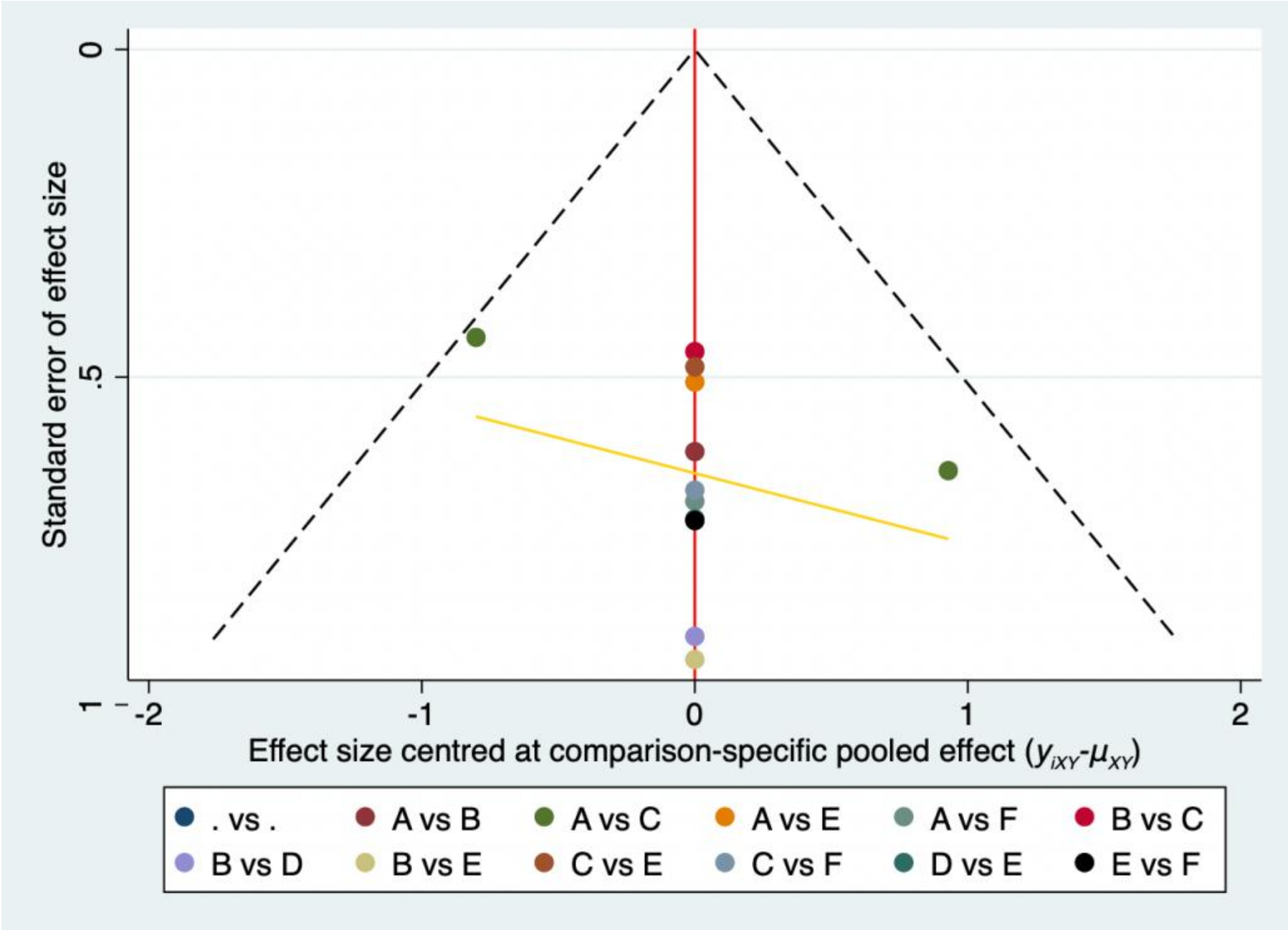

F 7 Comparison-adjusted funnel plot for the network meta-analysis of IL-6

Note: Blank control group (A), Aerobic exercise only (B), Aerobic plus resistance exercise (C), Aerobic exercise combined with other effective treatments (D), Pharmacological or psychological group(E), Aerobic exercise plus resistance exercise combined with other effective treatments(F).

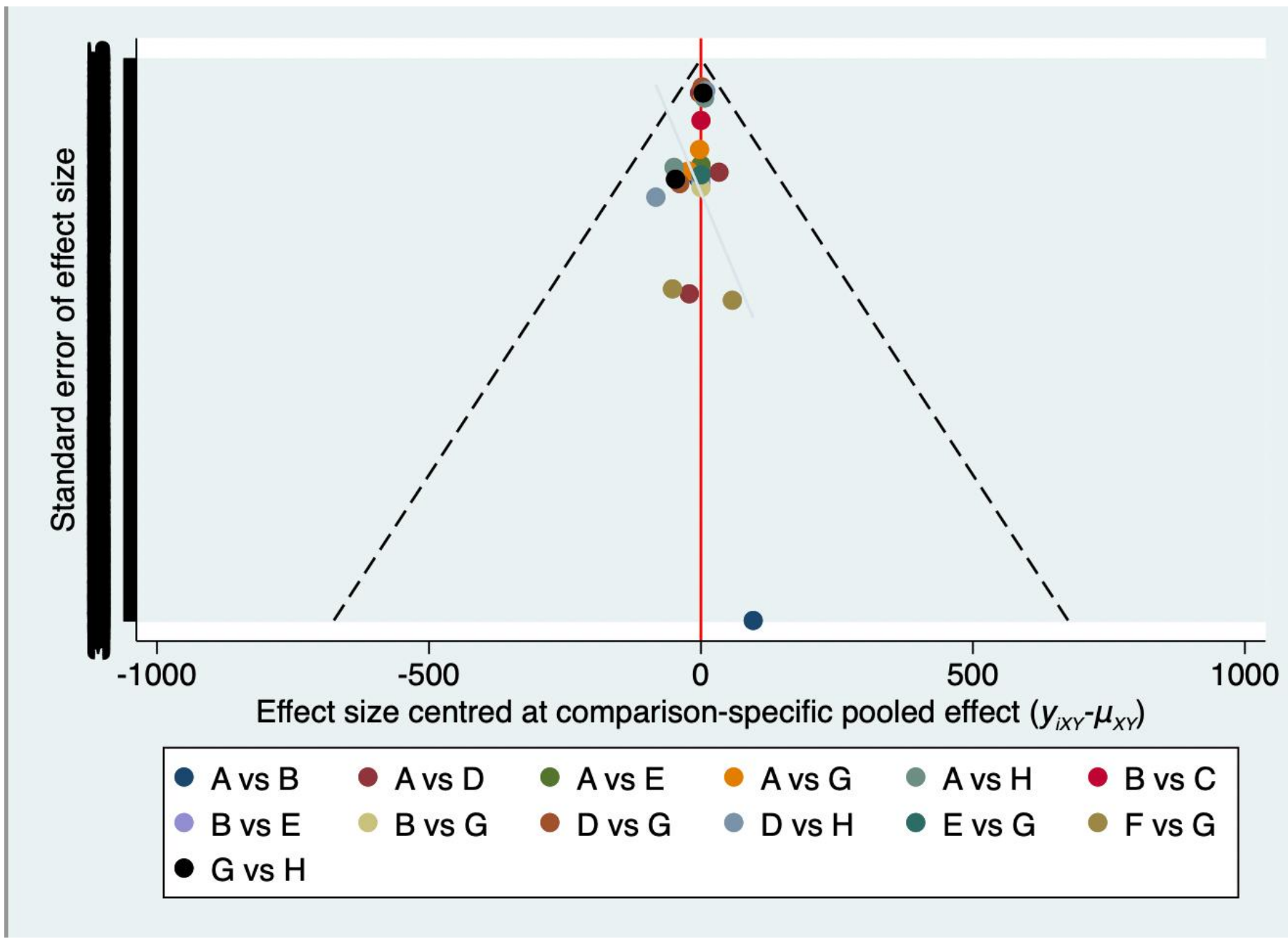

F 8 Comparison-adjusted funnel plot for the network meta-analysis of Total intake

Note: Blank control group (A), Aerobic exercise only (B), Resistance exercise only(C), Aerobic plus resistance exercise (D), Aerobic exercise combined with other effective treatments (E), Resistance exercise combined with other effective treatments (F), Pharmacological or psychological group(G), Aerobic exercise plus resistance exercise combined with other effective treatments(H).

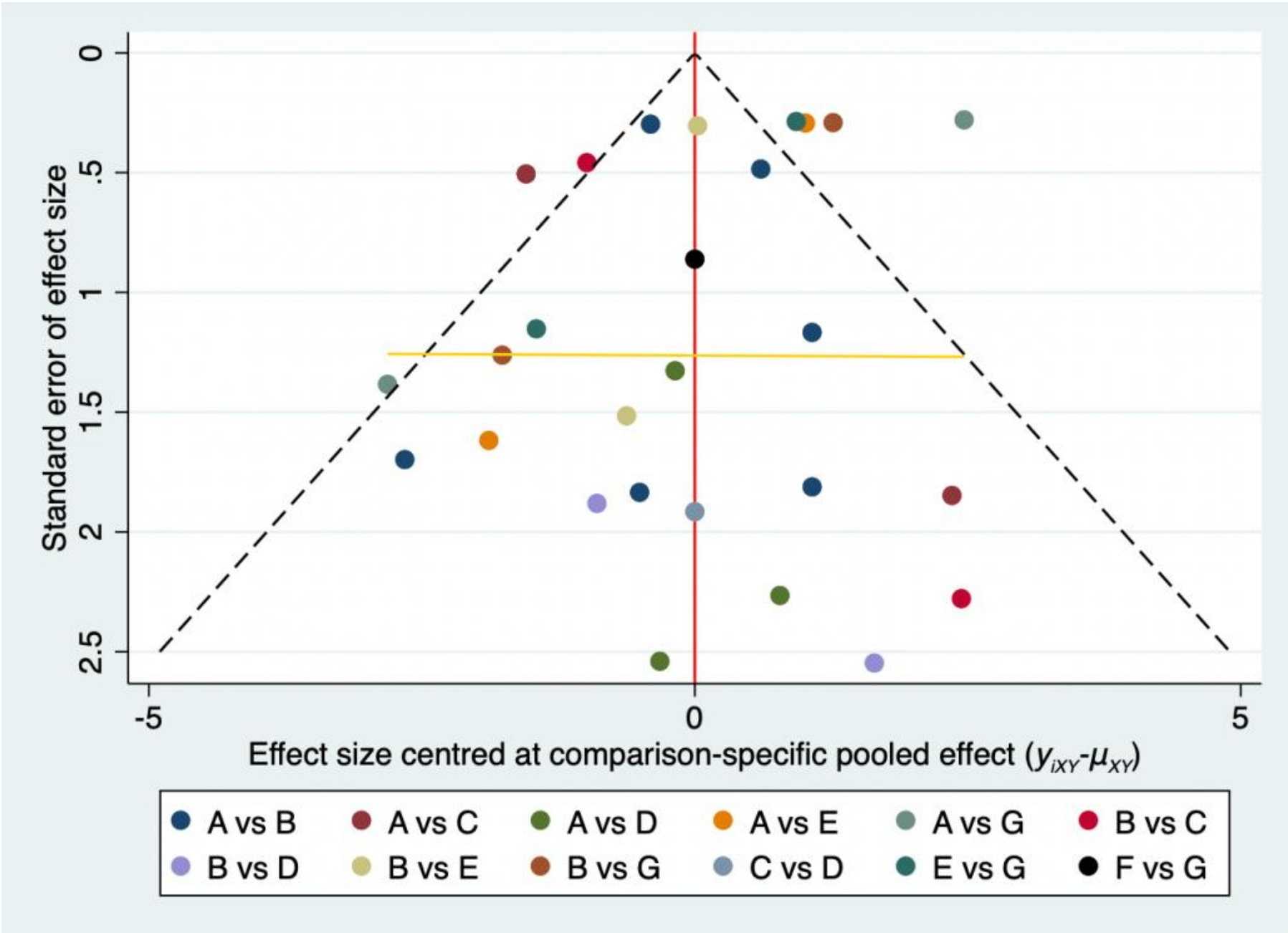

F 9 Comparison-adjusted funnel plot for the network meta-analysis of Insulin

Note: Blank control group (A), Aerobic exercise only (B), Resistance exercise only(C), Aerobic plus resistance exercise (D), Aerobic exercise combined with other effective treatments (E), Walking and jogging group (F), Pharmacological or psychological group(G).

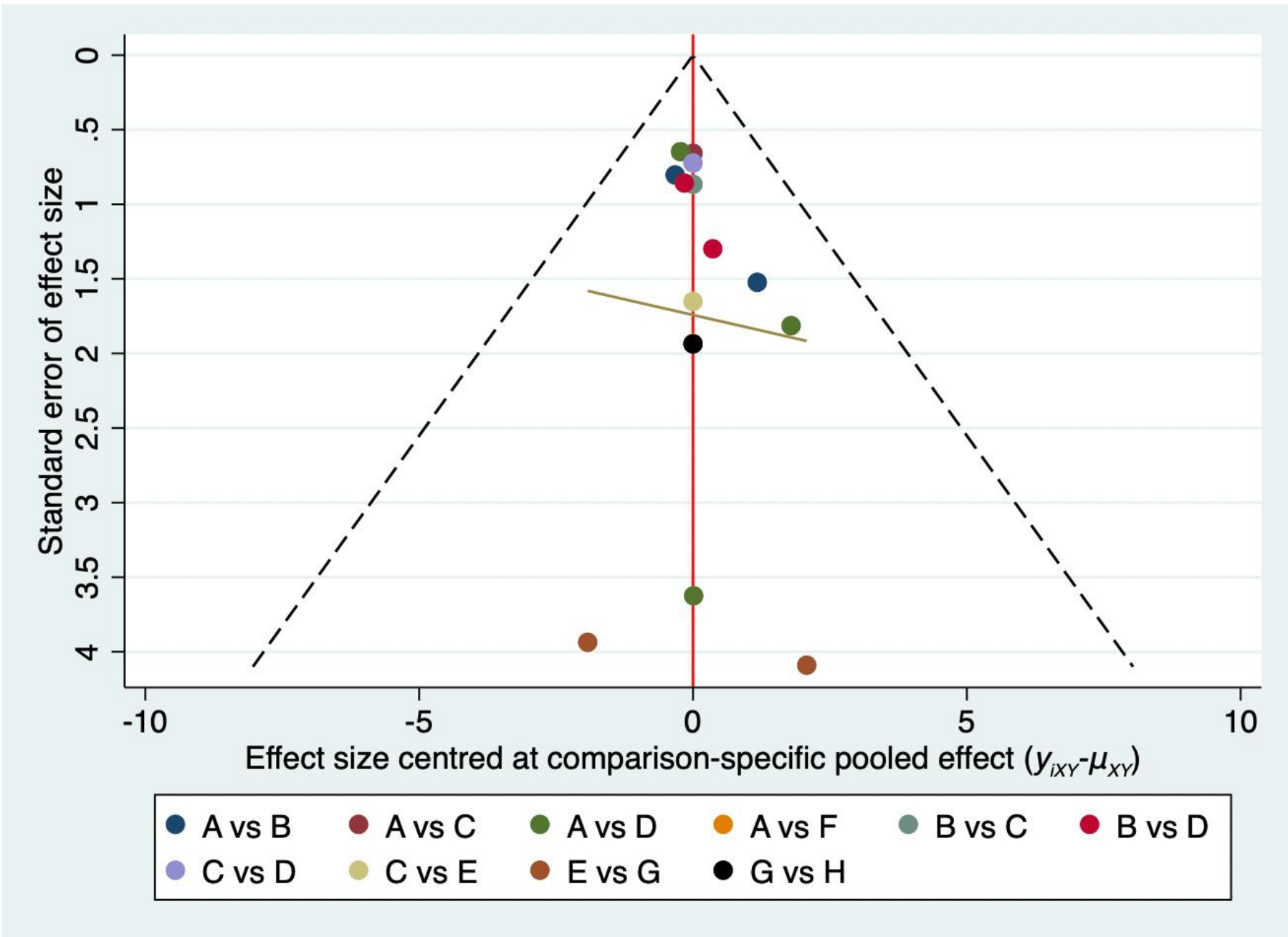

F 10 Comparison-adjusted funnel plot for the network meta-analysis of lean body mass

Note: Blank control group (A), Aerobic exercise only (B), Resistance exercise only(C), Aerobic plus resistance exercise (D), Resistance exercise combined with other effective treatments (E), Walking and jogging group (F), Pharmacological or psychological group(G), Aerobic exercise plus resistance exercise combined with other effective treatments(H).

Appendix 8

Primary Outcome

Study or Subgroup	Experimental Mean	Experimental SD	Experimental Total	Control Mean	Control SD	Control Total	Weight	Mean Difference IV, Random, 95% CI
AminiLari2017	28.37	2.57	13	28.17	2.57	13	4.2%	0.20 [−1.78, 2.18]
Bacchi2013	29.8	0.46	14	28.25	0.57	17	5.1%	1.55 [1.19, 1.91]
Banitalebi2019	28.59	4.35	14	29.82	3.37	14	3.4%	−1.23 [−4.11, 1.65]
Chen2010	31.3	4.2	50	32.8	4.4	44	4.3%	−1.50 [−3.25, 0.25]
Gholaman M2018	29.23	1.39	10	33.41	1.86	10	4.6%	−4.18 [−5.62, −2.74]
Golpasandi2025	26	1.8	8	30.5	1.06	8	4.6%	−4.50 [−5.95, −3.05]
João P. Magalhães MSc2018	30.6	5.1	16	30.7	4.9	22	3.1%	−0.10 [−3.33, 3.13]
Karstoft2013	29.6	1.6	12	29.8	1.9	8	4.4%	−0.20 [−1.80, 1.40]
Mohamed Seyam 1, Faizan Kashoo 2020	25.7	1.37	33	26.8	1.38	33	5.0%	−1.10 [−1.76, −0.44]
Otten2018	29.1	1.7	13	29	1.4	13	4.7%	0.10 [−1.10, 1.30]
Rajabi2022	28.97	2.3	8	34.03	3.57	8	3.4%	−5.06 [−8.00, −2.12]
Rajabi2024	27.07	2.68	11	35.15	3.75	11	3.5%	−8.08 [−10.80, −5.36]
Rezaeeshirazi2022	31.15	1.14	13	32.53	1.64	15	4.8%	−1.38 [−2.42, −0.34]
Sabag2020	34.2	1.1	12	36.2	1.9	13	4.7%	−2.00 [−3.21, −0.79]
Sabine Lambers2008	28.25	1.85	17	30.3	4.34	11	3.6%	−2.05 [−4.76, 0.66]
Saeidi2021	26.17	0.78	11	28.46	0.96	11	5.0%	−2.29 [−3.02, −1.56]
Sampath Kumar AmaravadilD2024	25.32	3.16	75	26.27	3.63	71	4.8%	−0.95 [−2.06, 0.16]
Sanudo2013	29.95	6.84	20	32.66	5.41	20	2.7%	−2.71 [−6.53, 1.11]
Shehab M Abd El-Kader2018	29.22	3.74	36	33.85	3.23	37	4.4%	−4.63 [−6.23, −3.03]
Sokolovska2020	31.9	5.89	26	32.4	5.45	14	2.8%	−0.50 [−4.14, 3.14]
Walid Kamal Abdelbasset2020	34.1	3.1	16	36.2	5.5	16	3.3%	−2.10 [−5.19, 0.99]
Winding2018	27.1	3.2	12	28.3	3.2	7	3.3%	−1.20 [−4.18, 1.78]
Wycherley2008	30.7	1.43	13	31.5	1.23	16	4.8%	−0.80 [−1.78, 0.18]
Wycherley2010	31.4	4.3	17	31.7	5.1	16	3.2%	−0.30 [−3.53, 2.93]
Yamaguchi2011	26.2	5.3	11	26.2	4.91	8	2.2%	0.00 [−4.62, 4.62]
**Total (95% CI)**			**481**			**456**	**100.0%**	**−1.78 [−2.70, −0.85]**

Heterogeneity: Tau^2 = 4.32; Chi^2 = 284.20, df = 24 (P < 0.00001); I^2 = 92%
Test for overall effect: Z = 3.78 (P = 0.0002)

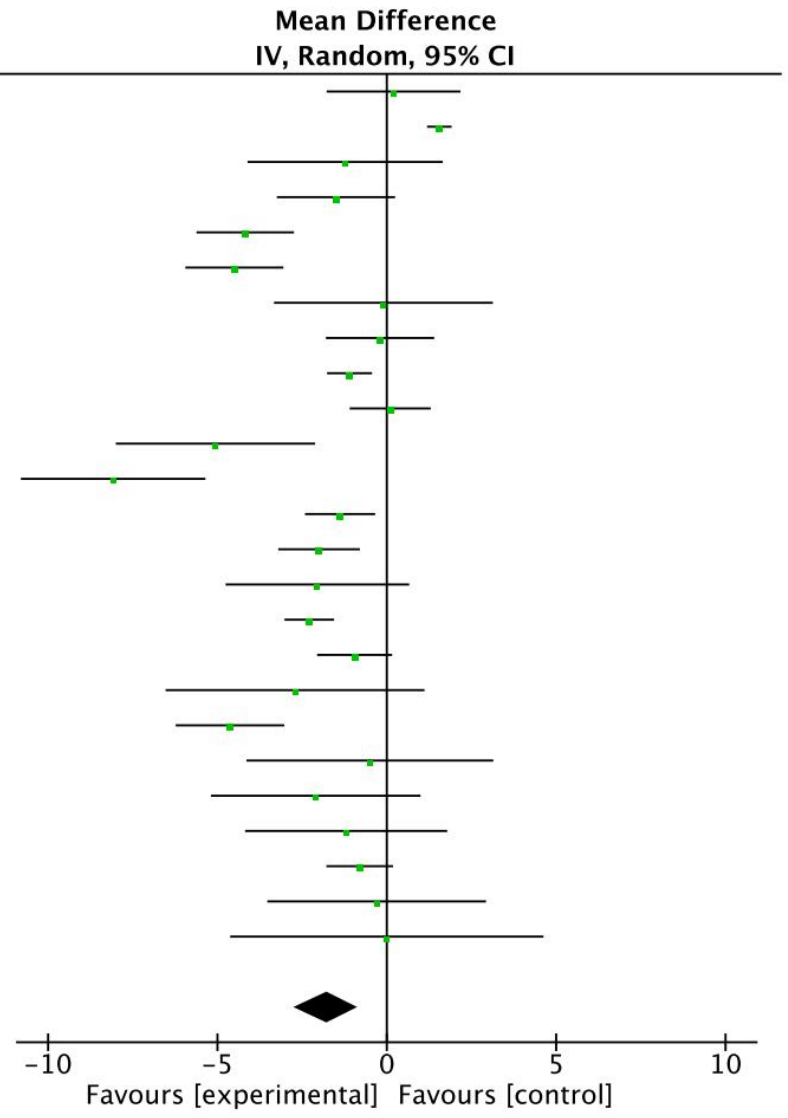

F 1 Forest plot for pairwise meta-analysis of BMI

Study or Subgroup	Experimental Mean	Experimental SD	Experimental Total	Control Mean	Control SD	Control Total	Weight	Mean Difference IV, Random, 95% CI
Bacchi2013	4.3	0.12	14	4.58	0.25	17	8.6%	−0.28 [−0.41, −0.15]
Bonfante2022	4.54	0.93	17	5.03	0.95	17	5.2%	−0.49 [−1.12, 0.14]
Chen2010	4.82	1.03	44	4.54	0.93	50	7.0%	0.28 [−0.12, 0.68]
Gholaman M2018	5.27	0.31	10	6.65	0.21	10	8.1%	−1.38 [−1.61, −1.15]
Karstoft2013	5.5	0.3	12	5.9	0.4	8	7.5%	−0.40 [−0.73, −0.07]
Otten2017	3.7	0.25	14	3.9	0.45	15	8.0%	−0.20 [−0.46, 0.06]
Sabag2020	4.4	0.2	12	4.6	0.3	13	8.3%	−0.20 [−0.40, −0.00]
Sabine Lambers2008	4.6	0.67	17	5	1.18	11	4.4%	−0.40 [−1.17, 0.37]
Saeidi2021	5.32	0.23	12	6.23	0.26	11	8.3%	−0.91 [−1.11, −0.71]
Sokolovska2020	5.1	0.9	26	5.3	1.4	14	4.1%	−0.20 [−1.01, 0.61]
Walid Kamal Abdelbasset2020	4.56	0.21	16	4.8	0.21	16	8.6%	−0.24 [−0.39, −0.09]
Winding2018	4.6	0.9	12	3.8	0.6	7	4.9%	0.80 [0.12, 1.48]
Wycherley2008	3.83	0.21	16	4.27	0.33	16	8.4%	−0.44 [−0.63, −0.25]
Wycherley2010	3.5	0.9	17	4.1	1.1	16	4.9%	−0.60 [−1.29, 0.09]
Yamaguchi2011	4.53	0.93	11	4.83	1.05	8	3.6%	−0.30 [−1.21, 0.61]
**Total (95% CI)**			**250**			**229**	**100.0%**	**−0.36 [−0.59, −0.14]**

Heterogeneity: Tau^2 = 0.15; Chi^2 = 130.96, df = 14 (P < 0.00001); I^2 = 89%
Test for overall effect: Z = 3.18 (P = 0.001)

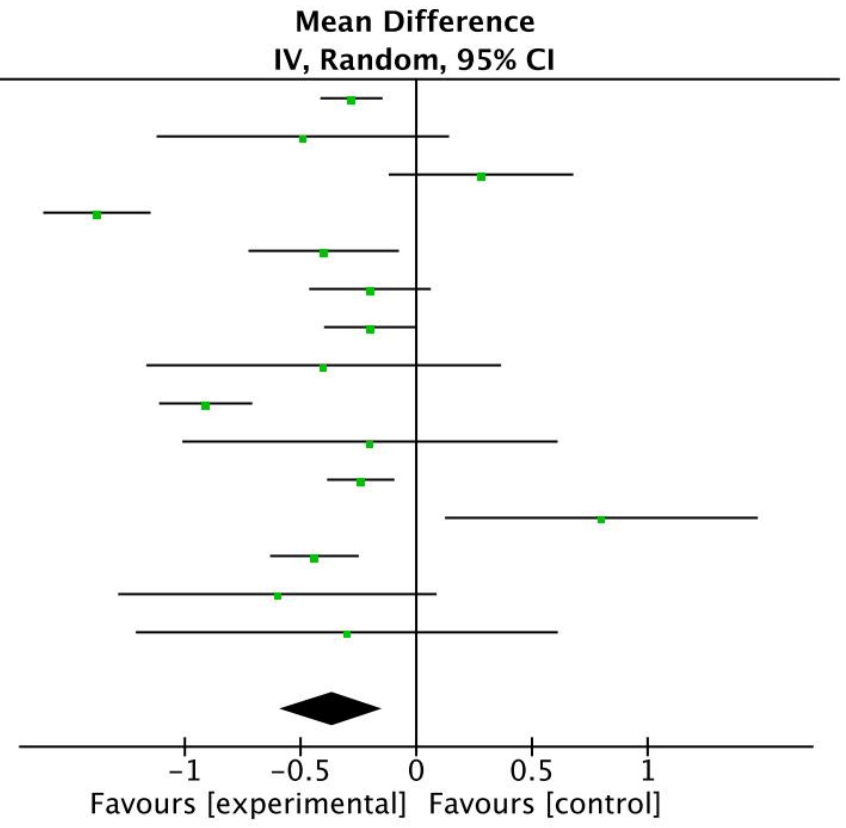

F 2 Forest plot for pairwise meta-analysis of TC

Study or Subgroup	Experimental Mean	Experimental SD	Experimental Total	Control Mean	Control SD	Control Total	Weight	Mean Difference IV, Random, 95% CI
Bacchi2013	1.27	0.22	14	1.44	0.17	17	11.4%	−0.17 [−0.31, −0.03]
Bonfante2022	1.4	0.59	17	1.63	0.73	17	3.6%	−0.23 [−0.68, 0.22]
Chen2010	1.93	0.38	50	2.13	0.43	44	10.4%	−0.20 [−0.37, −0.03]
Dunstan, D. W.Daly2002	1.6	0.5	16	1.72	0.69	13	3.6%	−0.12 [−0.57, 0.33]
Gholaman M2018	1.63	0.15	10	1.76	0.15	10	11.7%	−0.13 [−0.26, 0.00]
Karstoft2013	1.5	0.3	12	1.9	0.2	12	9.0%	−0.40 [−0.60, −0.20]
Otten2017	1.2	0.53	14	1.5	0.7	15	3.5%	−0.30 [−0.75, 0.15]
Sabag2020	1.9	0.3	13	2.1	0.3	13	8.1%	−0.20 [−0.43, 0.03]
Sabine Lambers2008	1.2	0.46	17	1.9	0.9	11	2.4%	−0.70 [−1.28, −0.12]
Saeidi2021	5.43	0.34	11	6.2	0.28	11	7.2%	−0.77 [−1.03, −0.51]
Sokolovska2020	1.8	0.9	26	2.4	2.2	14	0.6%	−0.60 [−1.80, 0.60]
Walid Kamal Abdelbasset2020	1.96	0.1	16	2.26	0.13	16	13.6%	−0.30 [−0.38, −0.22]
Winding2018	1.6	0.9	12	2.8	2.1	7	0.3%	−1.20 [−2.84, 0.44]
Wycherley2008	1.32	0.25	13	1.37	0.23	16	10.0%	−0.05 [−0.23, 0.13]
Wycherley2010	1.3	0.5	17	1.7	1.5	16	1.4%	−0.40 [−1.17, 0.37]
Yamaguchi2011	1.06	0.46	11	1.72	0.59	8	3.1%	−0.66 [−1.15, −0.17]
**Total (95% CI)**			**269**			**240**	**100.0%**	**−0.28 [−0.38, −0.19]**

Heterogeneity: Tau^2 = 0.02; Chi^2 = 35.93, df = 15 (P = 0.002); I^2 = 58%
Test for overall effect: Z = 5.72 (P < 0.00001)

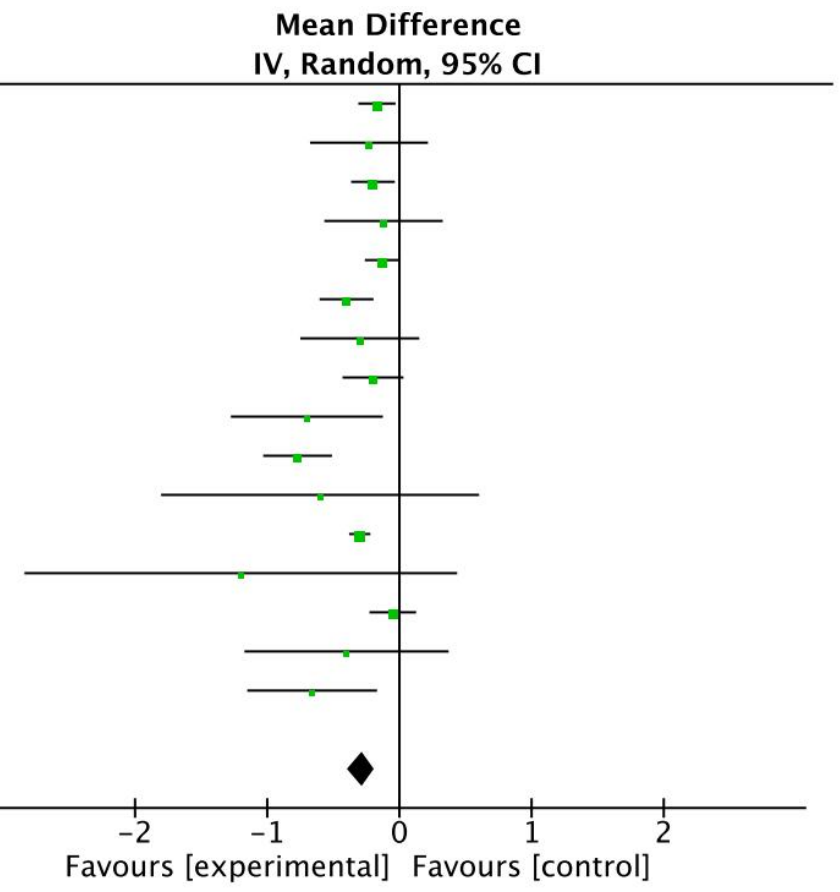

F 3 Forest plot for pairwise meta-analysis of TG

Study or Subgroup	Experimental Mean	Experimental SD	Experimental Total	Control Mean	Control SD	Control Total	Weight	Mean Difference IV, Random, 95% CI
Bonfante2022	2.72	0.53	17	3.09	0.86	17	5.9%	−0.37 [−0.85, 0.11]
Dunstan, D. W.Daly2002	1.26	0.1	16	1.37	0.23	16	12.6%	−0.11 [−0.23, 0.01]
Karstoft2013	3.4	0.1	12	3.7	0.3	8	10.8%	−0.30 [−0.52, −0.08]
Otten2017	2	0.4	15	2.3	0.38	14	9.4%	−0.30 [−0.58, −0.02]
Sabag2020	2.4	0.2	12	2.3	0.3	13	11.2%	0.10 [−0.10, 0.30]
Saeidi2021	3.46	0.21	11	4.08	0.12	11	12.3%	−0.62 [−0.76, −0.48]
Sokolovska2020	3.1	0.6	26	3.2	1.3	14	3.4%	−0.10 [−0.82, 0.62]
Walid Kamal Abdelbasset2020	2.34	0.12	16	2.46	0.12	16	13.2%	−0.12 [−0.20, −0.04]
Winding2018	2.7	0.9	12	1.9	0.7	7	3.4%	0.80 [0.07, 1.53]
Wycherley2008	1.98	0.16	16	2.13	0.36	13	10.9%	−0.15 [−0.36, 0.06]
Wycherley2010	1.9	0.9	17	2.4	1	16	4.0%	−0.50 [−1.15, 0.15]
Yamaguchi2011	2.88	0.98	11	2.98	0.85	8	2.8%	−0.10 [−0.93, 0.73]
**Total (95% CI)**			**181**			**153**	**100.0%**	**−0.19 [−0.35, −0.04]**

Heterogeneity: $Tau^2 = 0.04$; $Chi^2 = 57.68$, df = 11 (P < 0.00001); $I^2 = 81\%$
Test for overall effect: Z = 2.45 (P = 0.01)

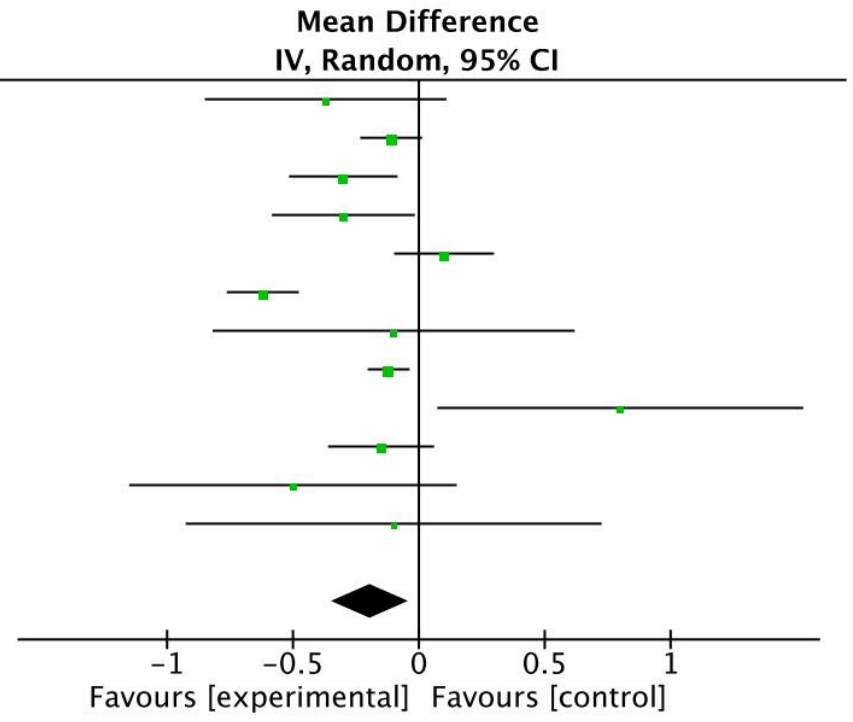

F 4 Forest plot for pairwise meta-analysis of LDL-C

Study or Subgroup	Experimental Mean	Experimental SD	Experimental Total	Control Mean	Control SD	Control Total	Weight	Mean Difference IV, Random, 95% CI
Bacchi2013	1.41	0.06	14	1.24	0.03	17	11.2%	0.17 [0.14, 0.20]
Bonfante2022	1.11	0.45	17	1.17	0.36	17	3.7%	−0.06 [−0.33, 0.21]
Chen2010	1.16	0.47	50	1.07	0.46	44	5.8%	0.09 [−0.10, 0.28]
Gholaman M2018	1.63	0.1	10	1.25	0.14	10	8.7%	0.38 [0.27, 0.49]
Karstoft2013	1.4	0.1	12	1.4	0.2	8	7.1%	0.00 [−0.15, 0.15]
Otten2017	1.08	0.08	14	0.84	0.08	14	10.5%	0.24 [0.18, 0.30]
Sabag2020	1.2	0.1	12	1.2	0.1	13	9.8%	0.00 [−0.08, 0.08]
Sabine Lambers2008	1.33	0.29	17	1.25	0.26	11	5.2%	0.08 [−0.13, 0.29]
Sokolovska2020	1.3	0.3	26	1.3	0.3	14	5.6%	0.00 [−0.19, 0.19]
Walid Kamal Abdelbasset2020	1.03	0.1	16	0.96	0.11	16	10.0%	0.07 [−0.00, 0.14]
Winding2018	1.3	0.4	12	1	0.3	7	3.0%	0.30 [−0.02, 0.62]
Wycherley2008	1.28	0.07	13	1.09	0.14	16	9.8%	0.19 [0.11, 0.27]
Wycherley2010	1	0.3	17	1.1	0.3	16	5.3%	−0.10 [−0.30, 0.10]
Yamaguchi2011	1.17	0.21	11	1.13	0.31	8	4.2%	0.04 [−0.21, 0.29]
**Total (95% CI)**			**241**			**211**	**100.0%**	**0.12 [0.05, 0.18]**

Heterogeneity: $Tau^2 = 0.01$; $Chi^2 = 64.16$, df = 13 (P < 0.00001); $I^2 = 80\%$
Test for overall effect: Z = 3.55 (P = 0.0004)

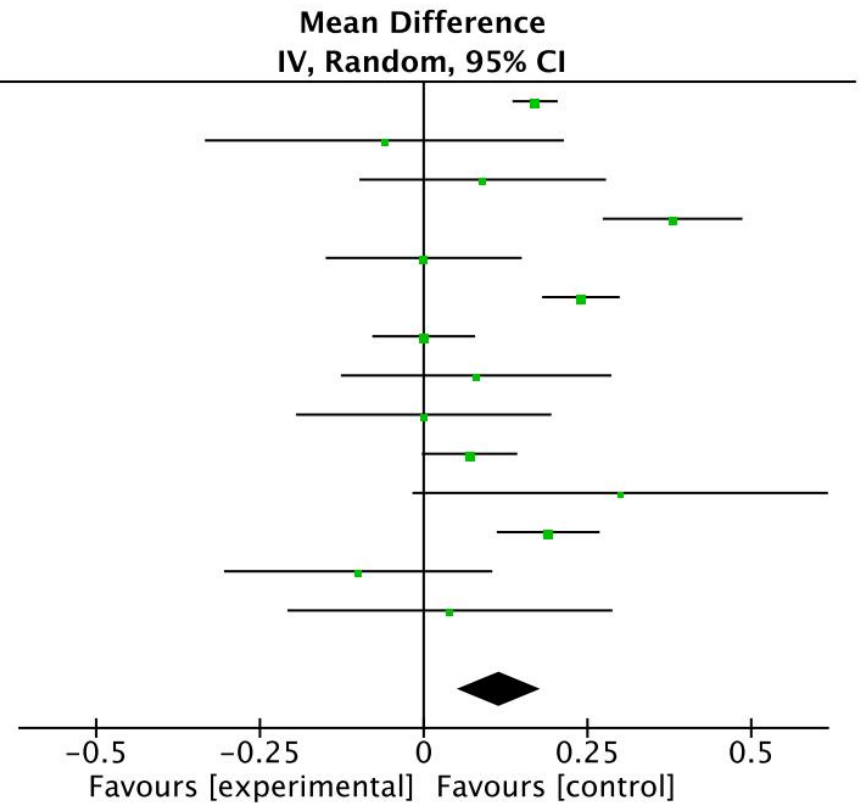

F 5 Forest plot for pairwise meta-analysis of HDL-C

Study or Subgroup	Experimental Mean	Experimental SD	Experimental Total	Control Mean	Control SD	Control Total	Weight	Mean Difference IV, Random, 95% CI
Banitalebi2019	0.95	0.24	14	1.42	0.71	14	7.3%	−0.47 [−0.86, −0.08]
Bonfante2022	4.05	2.25	17	4.96	3.23	17	5.3%	−0.91 [−2.78, 0.96]
Gholaman M2018	2.78	0.89	10	4.93	1.24	10	6.7%	−2.15 [−3.10, −1.20]
Golpasandi2025	4.3	0.07	8	7.13	0.12	8	7.4%	−2.83 [−2.93, −2.73]
João P. Magalhães MSc2018	1.9	1.2	16	2.4	1.7	22	6.7%	−0.50 [−1.42, 0.42]
Miller2021	1.64	1.17	87	1.96	1.06	77	7.3%	−0.32 [−0.66, 0.02]
Rezaeeshirazi2022	3.82	0.4	13	4	0.48	26	7.3%	−0.18 [−0.47, 0.11]
Sabag2020	2	0.4	13	2.1	0.4	13	7.3%	−0.10 [−0.41, 0.21]
Sampath Kumar AmaravadiID2024	3.35	1.82	75	5.38	2.82	71	6.9%	−2.03 [−2.80, −1.26]
Shehab M Abd El-Kader2018	3.98	1.31	36	5.97	2.24	37	6.8%	−1.99 [−2.83, −1.15]
Sokolovska2020	5.1	4.8	26	4.1	2.3	14	4.8%	1.00 [−1.20, 3.20]
Walid Kamal Abdelbasset2020	4.1	0.6	16	4.98	1.8	16	6.7%	−0.88 [−1.81, 0.05]
Winding2018	1.58	0.72	12	2.18	1.16	7	6.7%	−0.60 [−1.55, 0.35]
Wycherley2008	0.74	0.34	13	1.43	0.42	16	7.3%	−0.69 [−0.97, −0.41]
Yamaguchi2011	2.31	0.76	11	2.77	2.6	8	5.3%	−0.46 [−2.32, 1.40]
**Total (95% CI)**			**367**			**356**	**100.0%**	**−0.92 [−1.73, −0.10]**

Heterogeneity: $Tau^2 = 2.34$; $Chi^2 = 861.49$, df = 14 (P < 0.00001); $I^2 = 98\%$
Test for overall effect: Z = 2.21 (P = 0.03)

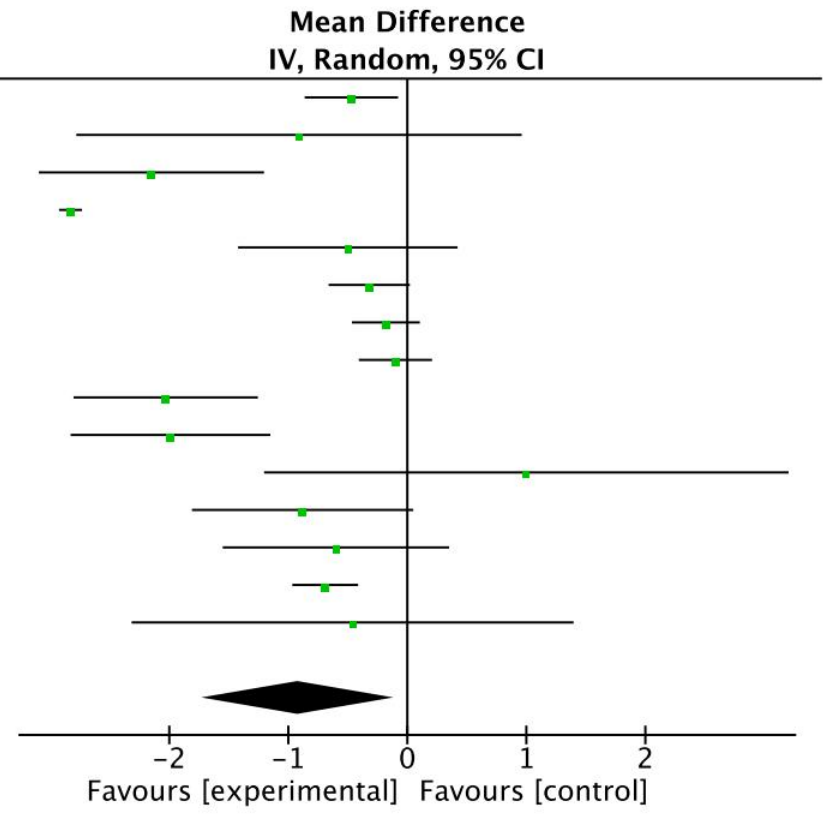

F 6 Forest plot for pairwise meta-analysis of HOMA-IR

Secondary Outcome

Study or Subgroup	Experimental Mean	Experimental SD	Experimental Total	Control Mean	Control SD	Control Total	Weight	Mean Difference IV, Random, 95% CI
Banitalebi2019	1.5	1.32	14	1.88	2.01	14	35.5%	−0.38 [−1.64, 0.88]
Rajabi2022	5.15	1.34	8	12.56	3.71	8	29.2%	−7.41 [−10.14, −4.68]
Saeidi2021	2.64	2.01	11	4.96	1.09	11	35.2%	−2.32 [−3.67, −0.97]
**Total (95% CI)**			**33**			**33**	**100.0%**	**−3.12 [−6.22, −0.01]**

Heterogeneity: $Tau^2 = 6.65$; $Chi^2 = 21.57$, df = 2 (P < 0.0001); $I^2 = 91\%$
Test for overall effect: Z = 1.97 (P = 0.05)

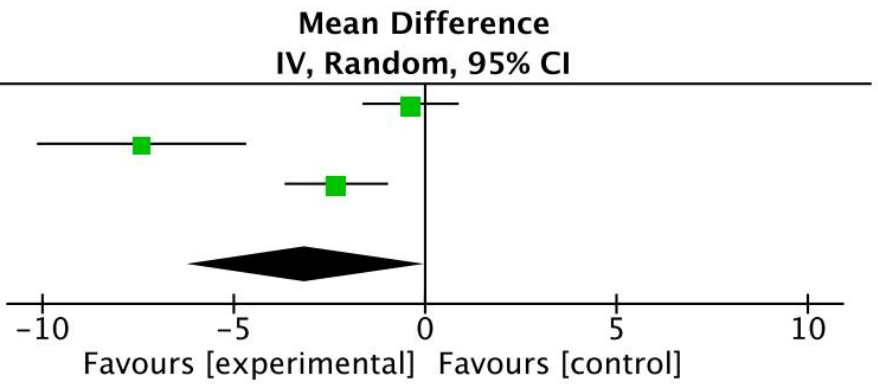

F 7 Forest plot for pairwise meta-analysis of IL-6

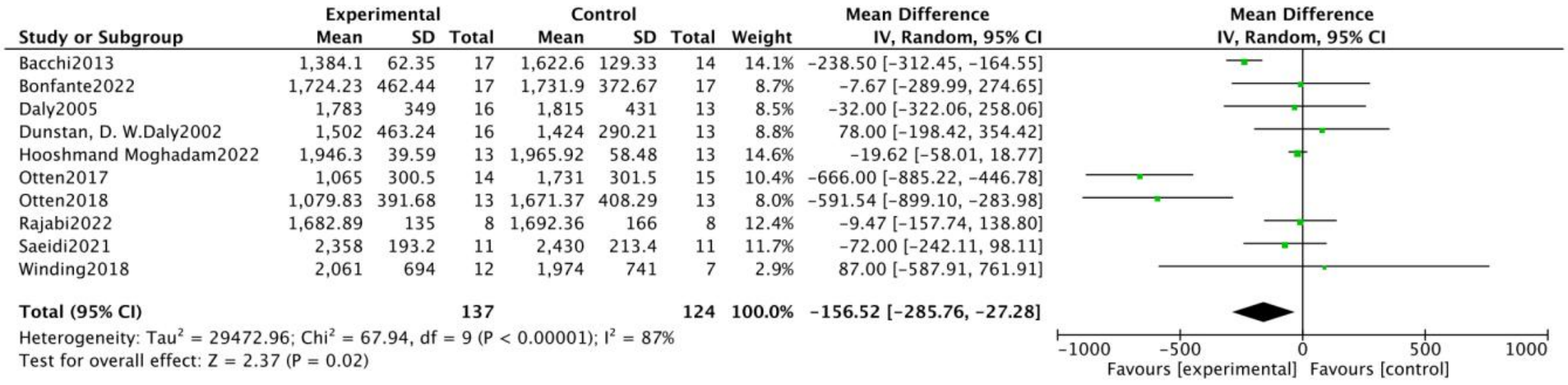

Study or Subgroup	Experimental Mean	Experimental SD	Experimental Total	Control Mean	Control SD	Control Total	Weight	Mean Difference IV, Random, 95% CI
Bacchi2013	1,384.1	62.35	17	1,622.6	129.33	14	14.1%	−238.50 [−312.45, −164.55]
Bonfante2022	1,724.23	462.44	17	1,731.9	372.67	17	8.7%	−7.67 [−289.99, 274.65]
Daly2005	1,783	349	16	1,815	431	13	8.5%	−32.00 [−322.06, 258.06]
Dunstan, D. W.Daly2002	1,502	463.24	16	1,424	290.21	13	8.8%	78.00 [−198.42, 354.42]
Hooshmand Moghadam2022	1,946.3	39.59	13	1,965.92	58.48	13	14.6%	−19.62 [−58.01, 18.77]
Otten2017	1,065	300.5	14	1,731	301.5	15	10.4%	−666.00 [−885.22, −446.78]
Otten2018	1,079.83	391.68	13	1,671.37	408.29	13	8.0%	−591.54 [−899.10, −283.98]
Rajabi2022	1,682.89	135	8	1,692.36	166	8	12.4%	−9.47 [−157.74, 138.80]
Saeidi2021	2,358	193.2	11	2,430	213.4	11	11.7%	−72.00 [−242.11, 98.11]
Winding2018	2,061	694	12	1,974	741	7	2.9%	87.00 [−587.91, 761.91]
**Total (95% CI)**			**137**			**124**	**100.0%**	**−156.52 [−285.76, −27.28]**

Heterogeneity: $Tau^2 = 29472.96$; $Chi^2 = 67.94$, df = 9 ($P < 0.00001$); $I^2 = 87\%$
Test for overall effect: Z = 2.37 (P = 0.02)

F 8 Forest plot for pairwise meta-analysis of Total intake

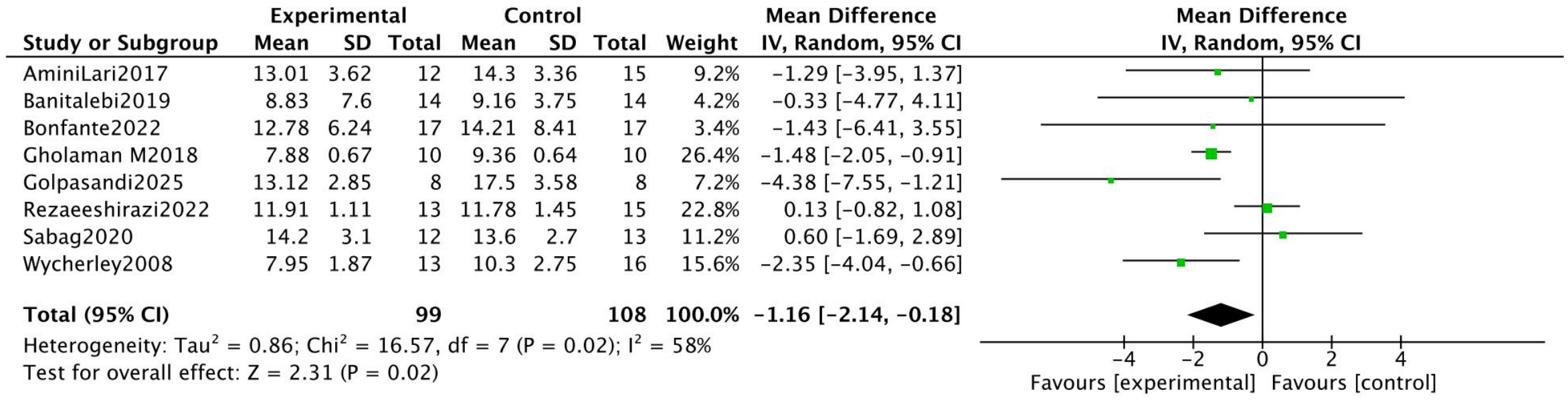

Study or Subgroup	Experimental Mean	Experimental SD	Experimental Total	Control Mean	Control SD	Control Total	Weight	Mean Difference IV, Random, 95% CI
AminiLari2017	13.01	3.62	12	14.3	3.36	15	9.2%	−1.29 [−3.95, 1.37]
Banitalebi2019	8.83	7.6	14	9.16	3.75	14	4.2%	−0.33 [−4.77, 4.11]
Bonfante2022	12.78	6.24	17	14.21	8.41	17	3.4%	−1.43 [−6.41, 3.55]
Gholaman M2018	7.88	0.67	10	9.36	0.64	10	26.4%	−1.48 [−2.05, −0.91]
Golpasandi2025	13.12	2.85	8	17.5	3.58	8	7.2%	−4.38 [−7.55, −1.21]
Rezaeeshirazi2022	11.91	1.11	13	11.78	1.45	15	22.8%	0.13 [−0.82, 1.08]
Sabag2020	14.2	3.1	12	13.6	2.7	13	11.2%	0.60 [−1.69, 2.89]
Wycherley2008	7.95	1.87	13	10.3	2.75	16	15.6%	−2.35 [−4.04, −0.66]
**Total (95% CI)**			**99**			**108**	**100.0%**	**−1.16 [−2.14, −0.18]**

Heterogeneity: $Tau^2 = 0.86$; $Chi^2 = 16.57$, df = 7 (P = 0.02); $I^2 = 58\%$
Test for overall effect: Z = 2.31 (P = 0.02)

F 9 Forest plot for pairwise meta-analysis of Iinsulin

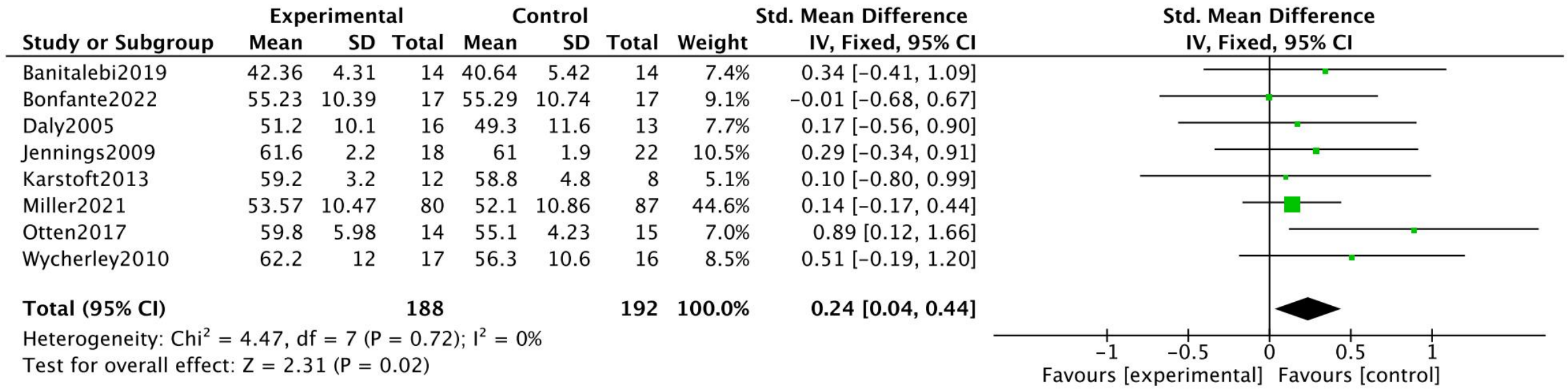

Study or Subgroup	Experimental Mean	Experimental SD	Experimental Total	Control Mean	Control SD	Control Total	Weight	Std. Mean Difference IV, Fixed, 95% CI
Banitalebi2019	42.36	4.31	14	40.64	5.42	14	7.4%	0.34 [−0.41, 1.09]
Bonfante2022	55.23	10.39	17	55.29	10.74	17	9.1%	−0.01 [−0.68, 0.67]
Daly2005	51.2	10.1	16	49.3	11.6	13	7.7%	0.17 [−0.56, 0.90]
Jennings2009	61.6	2.2	18	61	1.9	22	10.5%	0.29 [−0.34, 0.91]
Karstoft2013	59.2	3.2	12	58.8	4.8	8	5.1%	0.10 [−0.80, 0.99]
Miller2021	53.57	10.47	80	52.1	10.86	87	44.6%	0.14 [−0.17, 0.44]
Otten2017	59.8	5.98	14	55.1	4.23	15	7.0%	0.89 [0.12, 1.66]
Wycherley2010	62.2	12	17	56.3	10.6	16	8.5%	0.51 [−0.19, 1.20]
**Total (95% CI)**			**188**			**192**	**100.0%**	**0.24 [0.04, 0.44]**

Heterogeneity: $Chi^2 = 4.47$, df = 7 (P = 0.72); $I^2 = 0\%$
Test for overall effect: Z = 2.31 (P = 0.02)

F 10 Forest plot for pairwise meta-analysis of lean body mass
